# Supplementary material for: Pioneer activity distinguishes activating from non‐activating SOX2 binding sites
Source: EMBO J. 2023 Sep 11;42(20):e113150. doi: 10.15252/embj.2022113150 (PMC10577566; doi:10.15252/embj.2022113150)
Supplement: Supplementary file 1 — Appendix [file EMBJ-42-e113150-s004.pdf]

APPENDIX

Pioneer activity distinguishes activating from non-activating SOX2 binding sites

Michela Maresca[1,3], Teun van den Brand[1,3], Hangpeng Li[2], Hans Teunissen[1], James Davies[2], Elzo de Wit[1,*]

Table of contents:

Appendix Figure S1: Appendix Figure S1: Granular motif analysis for SOX2 and OCT4 depletion.

Appendix Figure S1

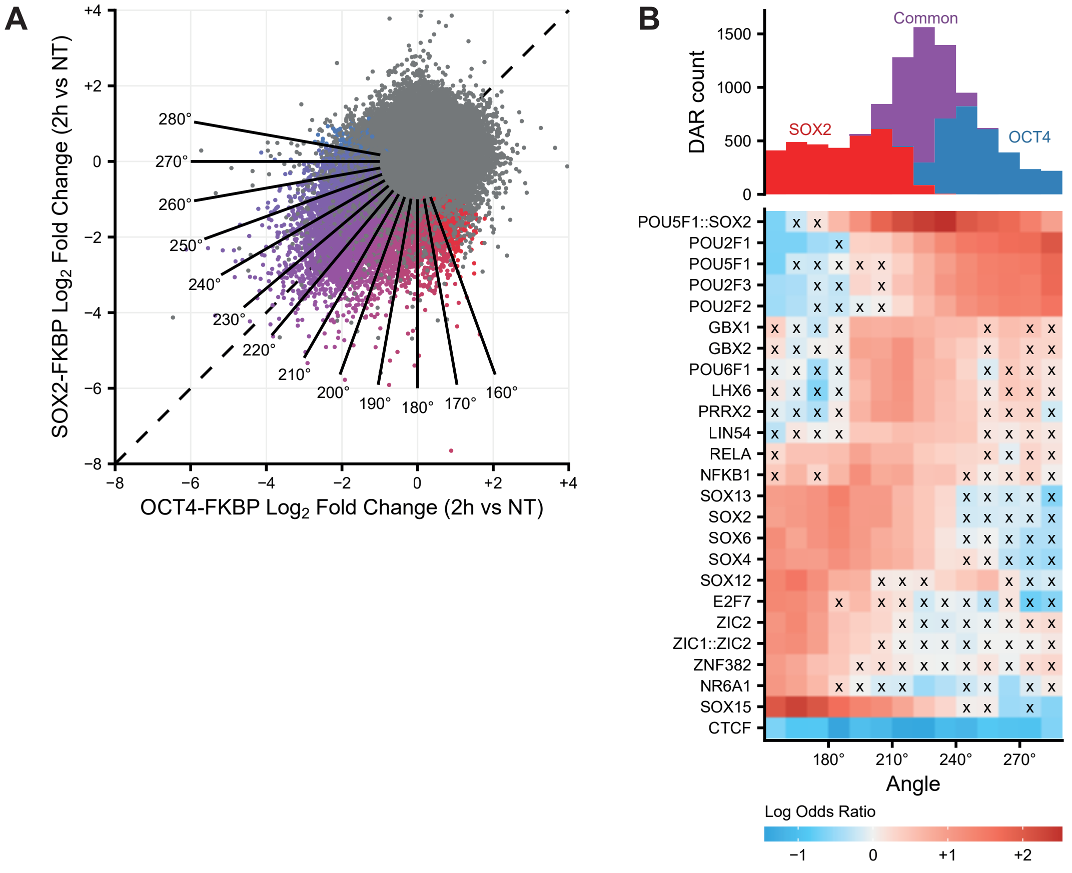

Appendix Figure S1: Granular motif analysis for SOX2 and OCT4 depletion.
A) Scatterplot showing ATACseq log_2 fold changes upon 2h of depletion of OCT4 (x-axis) and SOX2 (y-axis). Peaks that are downregulated differentially accessible regions (DARs) in either depletion are coloured according to angular bins, representing bins where the relative direction of change between the two depletions are similar. Breaks between angular bins are indicated with segments and their angles. B) Top: histogram of DAR categories from figure 2 by angular bin. Bottom: heatmap of motif enrichment analysis by angular bin, wherein effect size is indicated by log odds ratios of finding the motif in the bin relative to all other OCRs. Crosses denote non-significant depletions or enrichments.
