# Supplementary material for: Pioneer activity distinguishes activating from non‐activating SOX2 binding sites
Source: EMBO J. 2023 Sep 11;42(20):e113150. doi: 10.15252/embj.2022113150 (PMC10577566; doi:10.15252/embj.2022113150)
Supplement: Supplementary file 2 — Expanded View Figures pdf [file EMBJ-42-e113150-s003.pdf]

Expanded View Figures

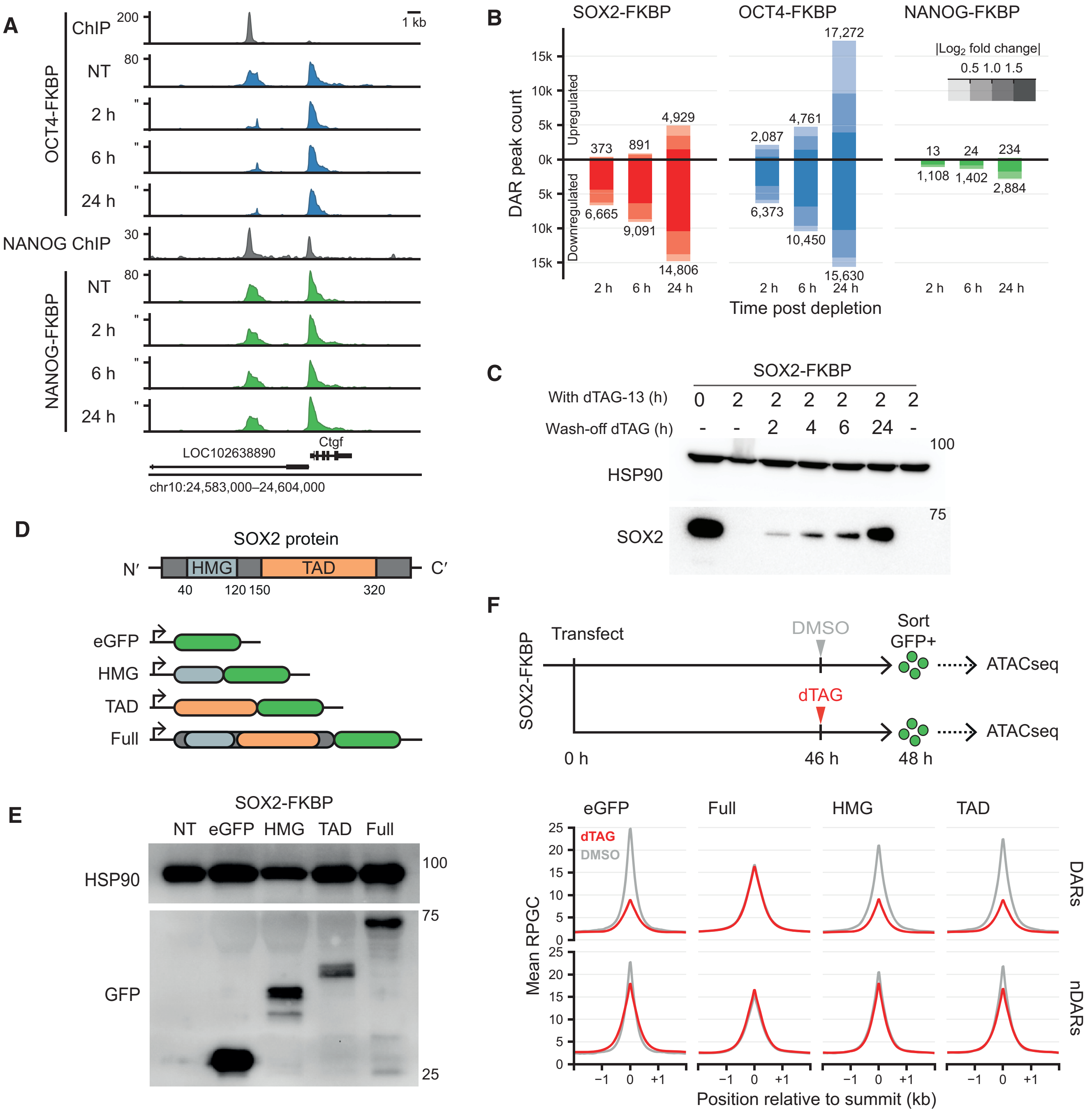

Figure EV1.

© 2023 The Authors

Figure EV1. Loss of accessibility after pioneer factor loss.

A Genomic tracks of accessibility changes by ATACseq in OCT4 and NANOG FKBP tagged lines measured in untreated condition (NT) and after OCT4 and NANOG depletion at the indicated timepoints. ChIPseq tracks for OCT4 in the OCT4-FKBP line and NANOG from publicly available data are shown on top of ATACseq. *Y*-axes refers to reads per genomic content (RPGC).

B Bar plot showing the number of differential accessible regions (DARs) after a time course of SOX2, OCT4 and NANOG depletion, in FKBP tagged lines.

C Western blot analysis of SOX2 expression level at 0 and 2 h of dTAG-13 and during a time course of dTAG-13 washoff. HSP90 was used as loading control.

D Schematic representation of SOX2 protein showing the DNA binding domain (HMG) and the trans-activation domain (TAD). To generate versions of the protein for ectopic expression, the HMG, TAD or full length SOX2 was cloned in frame with the eGFP sequence in an episomal expression vector.

E Western blot using an antibody against eGFP shows the fusion proteins running at the expected size. HSP90 was used as a loading control.

F Top; Representation of the experimental procedure for ATACseq after ectopic expression of the truncated SOX2-EGFP constructs. SOX2-FKBP cells were transfected with the plasmids and seeded for DMSO or dTAG-13 treatment. eGFP positive cells were sorted and ATACseq was performed on the eGFP expressing cells. Bottom; Line plot showing the average signal of ATACseq after overexpression of the plasmids after DMSO (gray) or dTAG13 treatment (red) for the endogenous SOX2-FKBP degradation. The average ATACseq signal is plotted for the DARs and nDARs as identified in Fig 1F.

Source data are available online for this figure.

Figure EV2. Quantitative and qualitative analysis of ATACseq and ChIPseq and Random forest classification reveals proteins and histone modifications that can predict differential accessibility.

A Euler diagram showing the overlap between differentially accessible regions (DARs) after 2 h of SOX2 depletion, all ATACseq peaks (OCR, open chromatin regions) and SOX2 DNA binding (SOX2 ChIP). Bottom panel shows the number of peaks in each overlap category. CCR: closed chromatin region, i.e., SOX2 ChIPseq peaks that do not overlap OCRs.

B Fraction of peaks containing 1 or more OCT4::SOX2 or SOX2 DNA binding motifs, stratified by whether OCRs, DARs and/or SOX2 binding sites as measured by ChIPseq, or combinations thereof.

C Vertical histogram of SOX2 ChIPseq peaks ranked by signal intensity, stratified by their overlap with downregulated DARs or lack of such overlap (nDAR), displayed in 200 peak bins.

D Left, top 25 (chromatin binding) factors in the Cistrome factors datasets whose overlap with all ATACseq peaks is predictive in random forest classification to discriminate the DARs from non-DARs (nDAR) peaks partially matched for SOX2 binding levels. ATACseq peaks were extended by 300 bp in both directions. Variable importance was calculated with subsampling inference, wherein the 95% confidence interval (CI) is indicated with a light color, the 50% CI with a darker color and the median with a point. Enrichment and depletion indicate higher and lower average overlap in the DAR than nDAR categories respectively. Right, top 25 histone modifications using the Cistrome histone datasets of 100 re-sampling.

E Tornado plots showing example differences between DARs and nDARs for SS18, CTCF, H3K79me2 and H3K64ac from publicly available ChIPseq datasets. Coverage indicates values in pre-processed data.

F Heatmap of ChromHMM defining chromatin states of different set of ATACseq peaks: other open chromatin regions (OCRs), DARs and partially SOX2-binding matched nDARs. The expected value was calculated under independence of proportions assumption, as they are calculated for a chi-squared test.

© 2023 The Authors

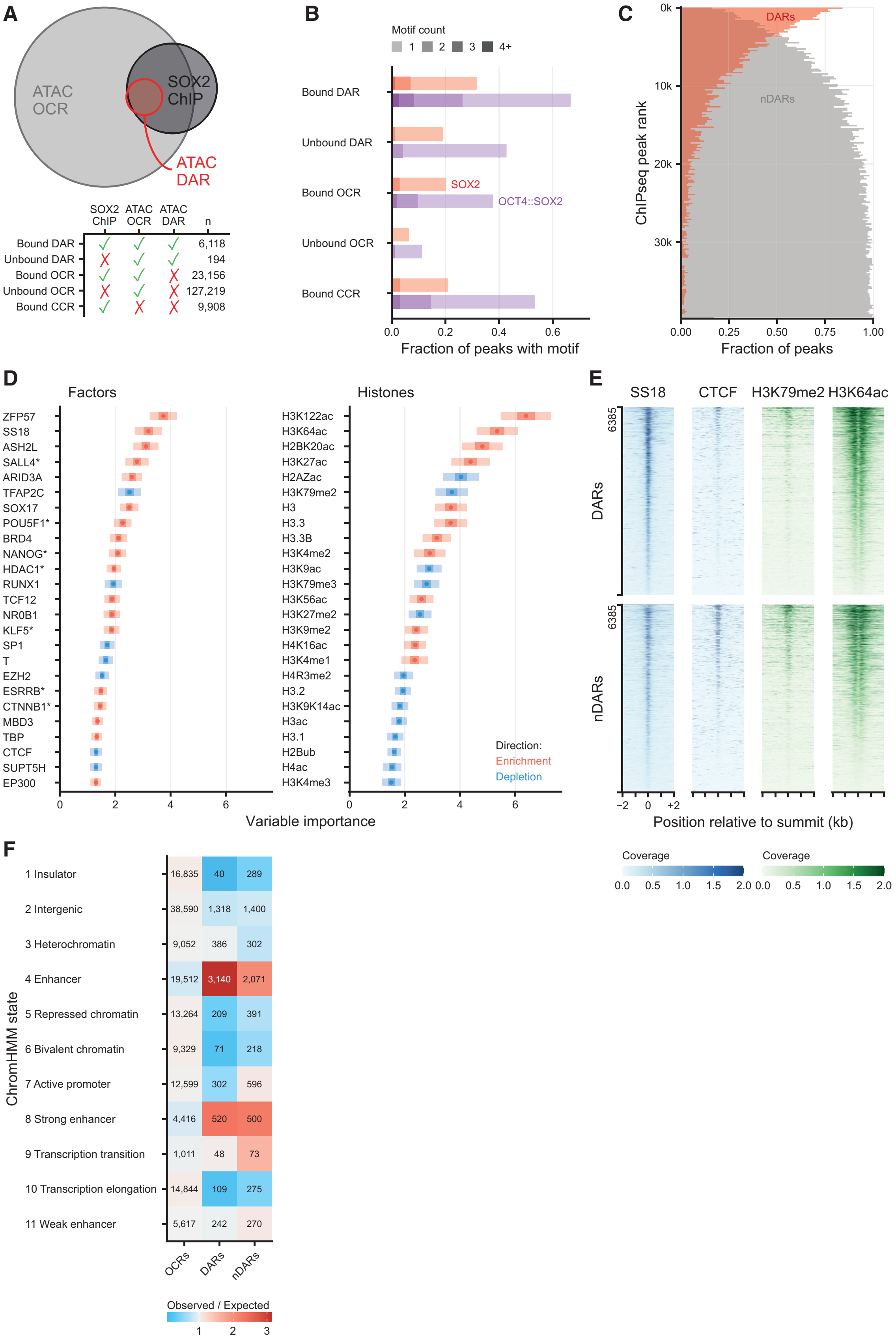

Figure EV2.

 © 2023 The Authors

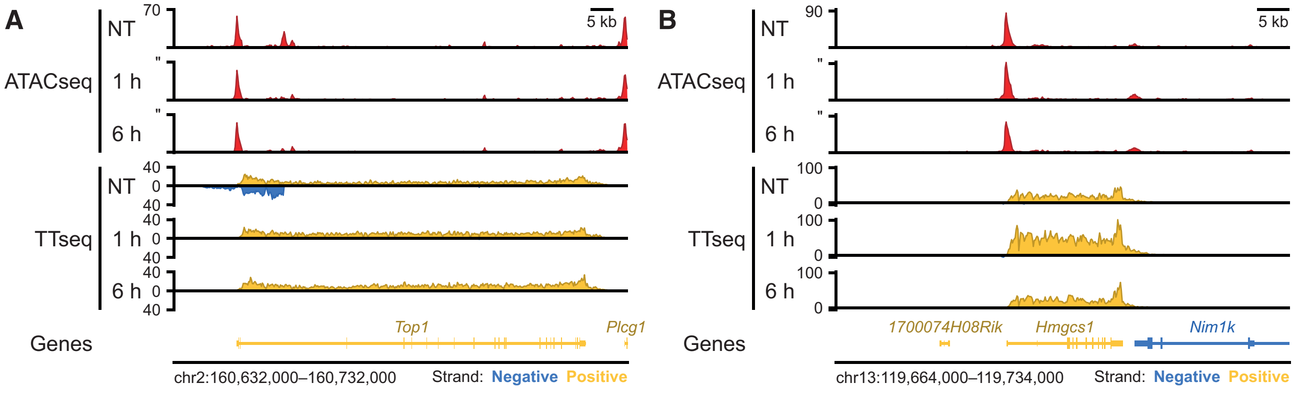

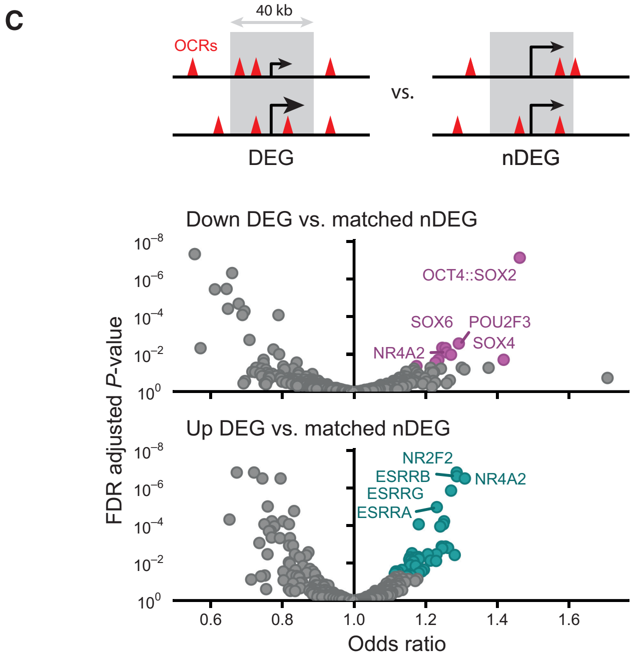

D

Pathway	Foreground Ratio	Background Ratio	*P*-value FDR adjusted
**1H Up DEGs**			
Cholesterlos biosynthesis	20/320	25/5704	$1.4 \cdot 10^{-18}$
Metabolism of steroids	23/320	77/5704	$3.7 \cdot 10^{-9}$
Metabolism of lipids	49/320	392/5704	$1.0 \cdot 10^{-5}$
Hemostasis	39/320	321/5704	$4.3 \cdot 10^{-4}$
Cardiac conduction	12/320	57/5704	$7.2 \cdot 10^{-3}$
**2H Up DEGs**			
Cholesterol biosynthesis	20/602	25/5704	$5.7 \cdot 10^{-13}$
Metabolism of steroids	24/602	77/5704	$2.3 \cdot 10^{-4}$
Cardiac conduction	16/602	57/5704	$4.8 \cdot 10^{-2}$
**6H Up DEGs**			
Ion homeostasis	15/665	36/5704	$2.7 \cdot 10^{-3}$
RHO GTPase cycle	63/665	319/5704	$2.7 \cdot 10^{-3}$
Cardiac conduction	19/665	57/5704	$2.7 \cdot 10^{-3}$
RAC1 GTPase cycle	31/665	121/5704	$2.7 \cdot 10^{-3}$
Muscle contraction	23/665	86/5704	$1.4 \cdot 10^{-4}$

Figure EV3. Upregulation of transcripts following SOX2 depletion.

A Example region showing changes in accessibility measured by ATACseq and transcription measured by TT_{chem}seq for the *Top1* locus in untreated (NT), 1 and 6 h of dTAG-13 treatment in SOX2-FKBP cells. *Y*-axes shows reads per genomic content.

B Same as in (A) but for *Hmgcs1* locus.

C Top: schematic of the widow selected for motif analysis around differentially expressed genes (DEGs) and expressed matched control non-DEGs (nDEGs). Bottom: volcano plot showing the motifs found in open chromatin regions (OCRs) in a 40 kb window centered on the TSSs of downregulated DEGs and at upregulated DEGs.

D Table showing reactome pathway (Gillespie *et al*, 2022) overrepresentation analysis results on TT_{chem}-seq DEGs.following SOX2 depletion. Table shows all significant hits at the FDR adjusted *P*-value threshold lower than 0.05, stratified by timepoint and direction. For 0.5 h and downregulated DEGs, no significant pathway enrichments were found.

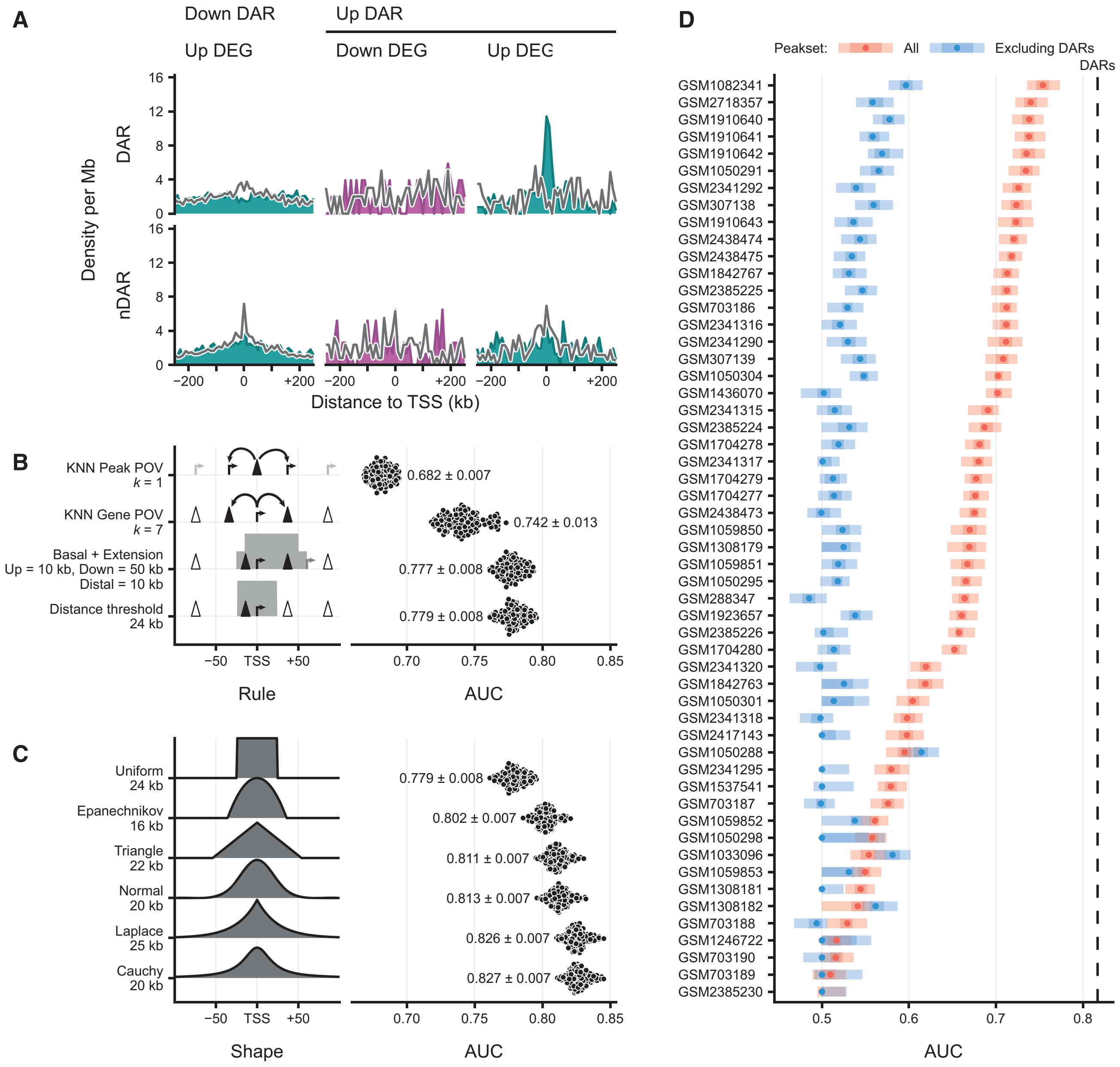

Figure EV4. Characterization of different association rules and chromatin features for the prediction of transcriptional changes.

A Density of peaks in 10 kb bins nearby transcription start sites (TSS) of upregulated (light blue) and downregulated (purple) differentially expressed genes (DEGs) and their expression-matched stable control (gray) non-DEGs (nDEGs). Panels indicate the set of peaks that were aligned to the TSS: down differentially accessible regions (DARs) and up DARs. Bottom row shows these densities for an equal number of stable non-DARs (nDAR) at the same gene sets.

B Predictive performance comparison of different association rules to discriminate downregulated DEGs upon SOX2 depletion at 2 h from matched nDEGs, with counts of associated (n)DAR peaks as predictors. *X*-axis metric notes the area under the receiver operator characteristic curve (AUC). Dots represent 100 re-samplings of the matched nDEGs. Numbers represent mean ± standard deviation. Optimal parameters for every rule indicated at the *Y*-axis labels, such as distance = 25 kb and *k* = 6, were chosen by performing a parameter sweep and choosing the parameter that minimized cross-validation error. Left part visually indicates association rule.

C Like (B), but for various kernel-based weighting functions instead of association rules, and weighted sums of peaks instead of counts as predictors. Left part gives visual indication of kernel shape. Numbers represent mean ± standard deviation.

D Predictive power on expression changes based on the many publicly available ChIPseq data, with or excluding peaks overlapping with DARs, for SOX2 in mESC-like cells (via Cistrome). The dotted line indicate the average predictive power of DARs for context. *Y*-axis gives Gene Expression Omnibus accession numbers for the datasets. *X*-axis metric notes the area under the receiver operator characteristic curve (AUC). Intervals and medians were calculated for 100 re-samplings of the matched nDEGs. The light shade gives the 95% inter-percentile range (IPR), the darker shade gives the inter-quartile range and the dots give the medians.

Source data are available online for this figure.

 © 2023 The Authors

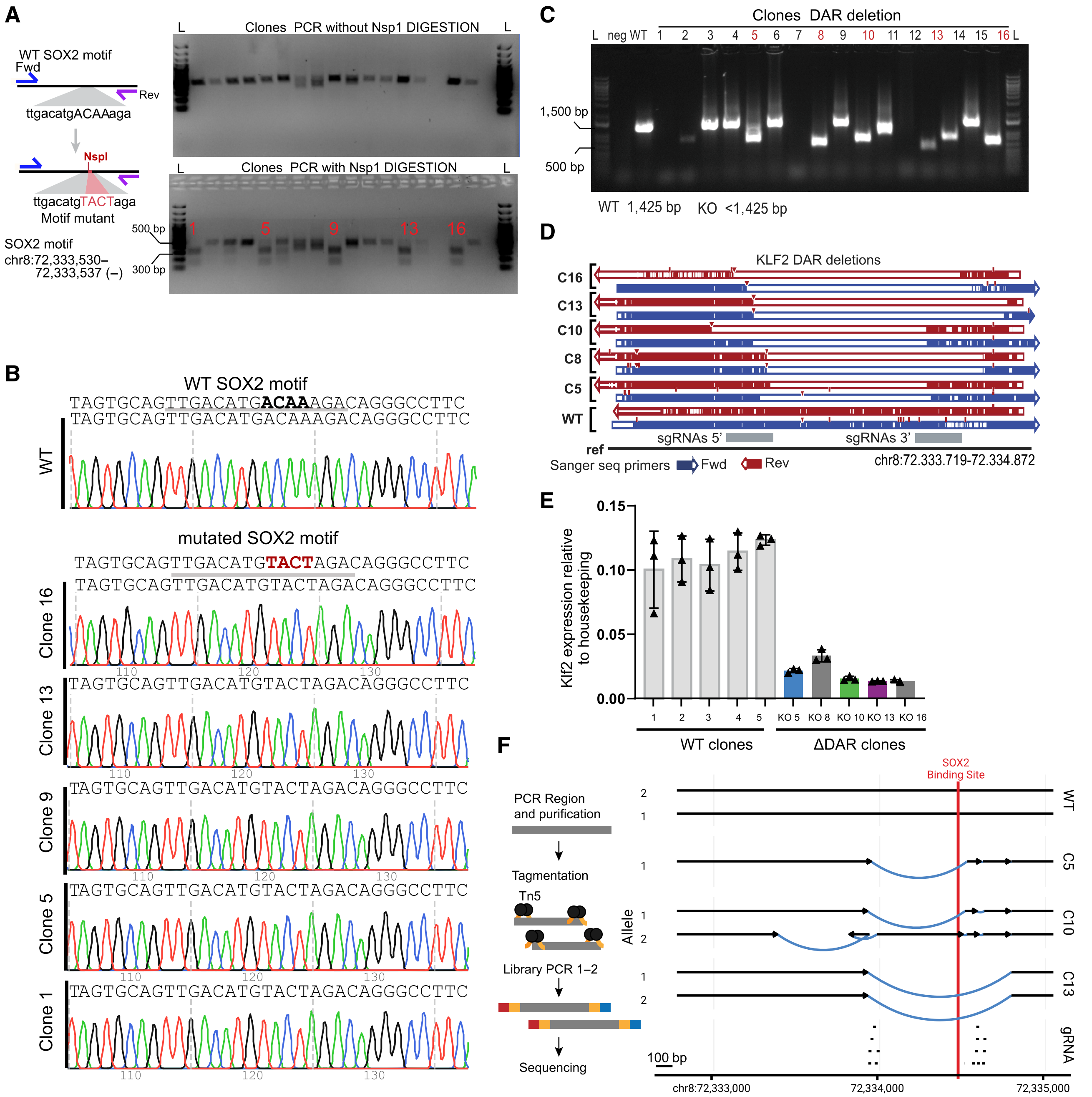

Figure EV5. Validation of genome editing of DAR KO and *Klf2* gene expression.

A Left: schematic of the procedure used for editing and selection of clones with homozygous mutation of the motif. Right: Top, DNA gel electrophoresis of the PCR product related to region selected for point mutant. Bottom, digestion of the PCR product (top) using NspI. Homozygous clones show 2 bands. Unedited clones show 1 band. L, ladder.

B Sanger tracks for WT and motif mutant clones.

C Gel electrophoresis of PCR for genotyping disruption of the DAR region in clones from the gene edited SOX2-FKBP parental cell line. Primers amplifying the targeted regions were used to control for the homozygous disruption compared to WT amplification. L: ladder, Neg: water control, DAR KO clones: clones selected for genotyping. In red, clones selected for further experiments.

D Validation of the disruption using Sanger sequencing in clones compared to non-edited clones. Blue: forward primer, red: reverse primer, gray: region targeted by sgRNAs 5′ and 3′ of the DAR.

E RT–qPCR of *Klf2* expression, similar as Fig 5B, but using an alternative set of primers, in 5 parental clones and the DAR KO clones. Expression is relative to housekeeping gene *Rsp26*. Error bar represent standard deviation of three biological replicates.

F Left panel shows simplified overview of the amplicon sequencing procedure. Right panel shows the most likely assembly based on the amplicon sequencing of the DAR regions in WT and KO clones. Region targeted by sgRNAs are shown at the bottom. Arrows indicate the centromere to telomere orientation. Black lines shows assembled sequence that is identical to the reference sequence. Blue lines show structural variants identified in the clones. Red indicates position of the SOX2 binding motif overlapping with a SOX2 ChIPseq peak.

© 2023 The Authors
