# Supplementary figures and images for: Pioneer activity distinguishes activating from non‐activating SOX2 binding sites
Source: EMBO J. 2023 Sep 11;42(20):e113150. doi: 10.15252/embj.2022113150 (PMC10577566; doi:10.15252/embj.2022113150)

Related to Figure EV 1C

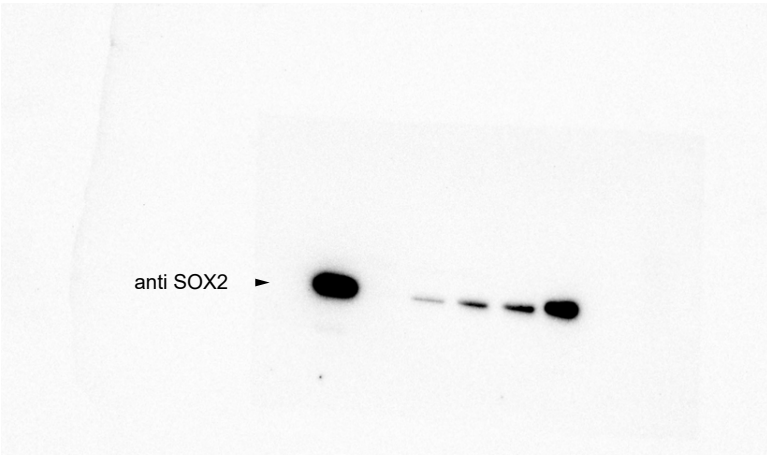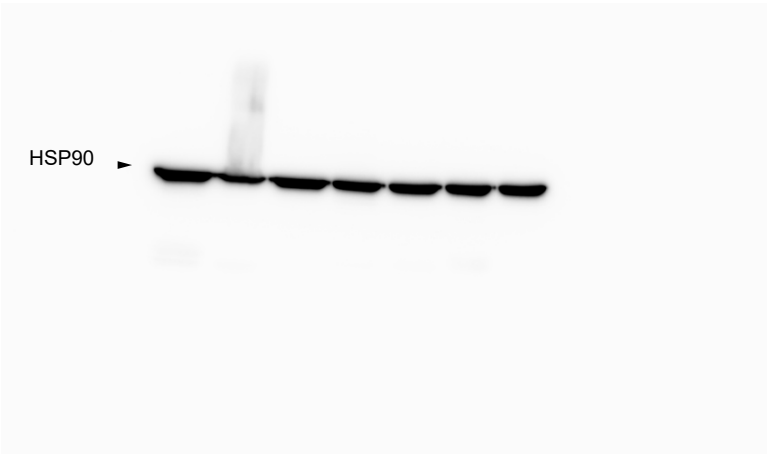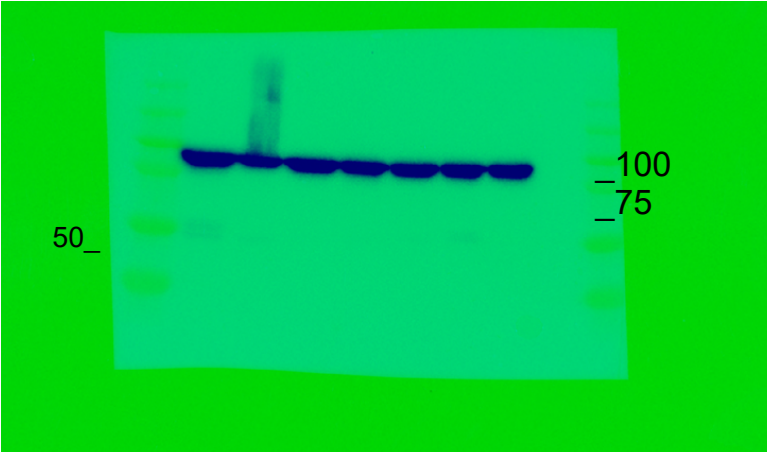

Supplement: Supplementary file 5 — Source Data for Expanded View [file EMBJ-42-e113150-s008.zip › Related to Figure EV1C.pdf]

Related to Figure EV 1E

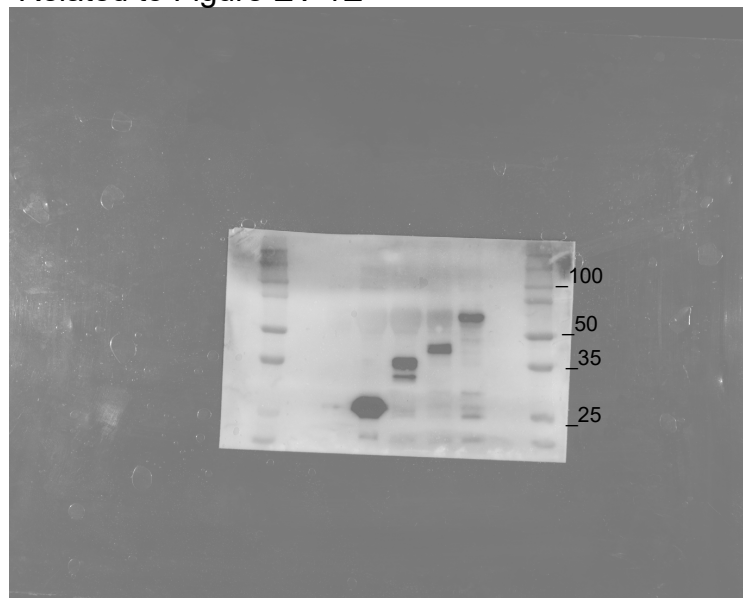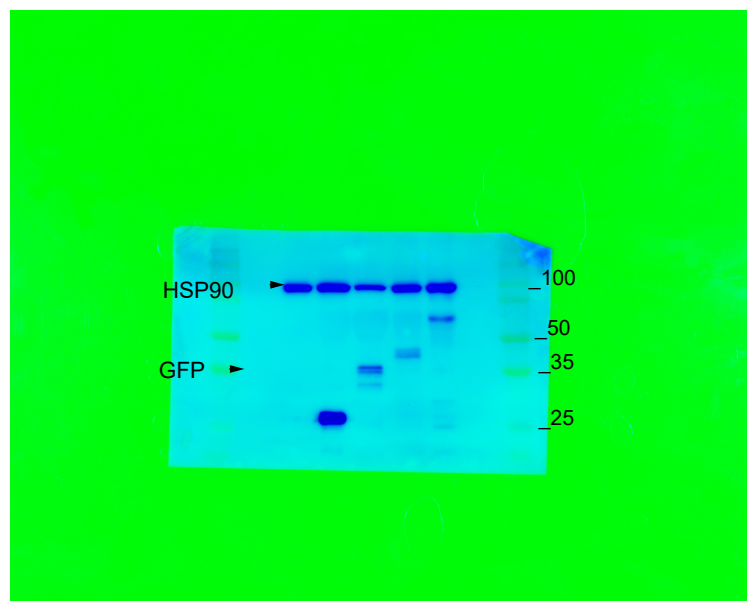

Supplement: Supplementary file 5 — Source Data for Expanded View [file EMBJ-42-e113150-s008.zip › Related to Figure EV1E.pdf]

Related to Figure EV 4A

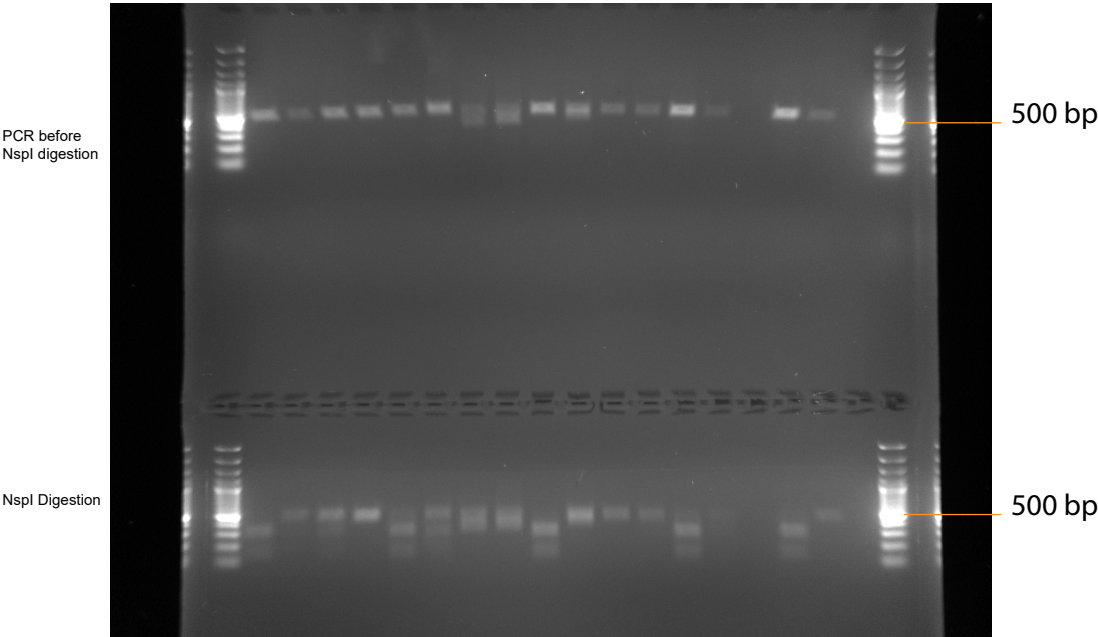

Supplement: Supplementary file 5 — Source Data for Expanded View [file EMBJ-42-e113150-s008.zip › Related to Figure EV4A.pdf]

Related to Figure EV 4C

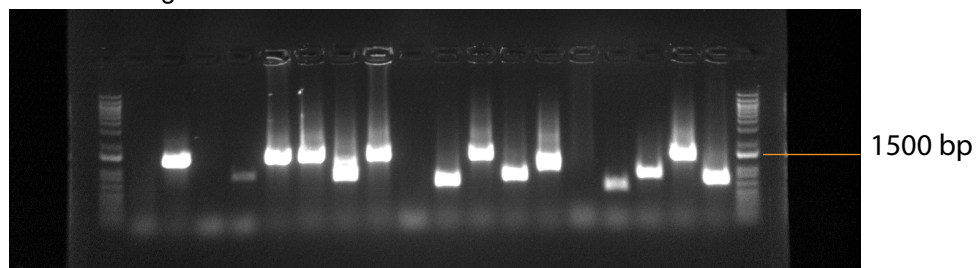

Supplement: Supplementary file 5 — Source Data for Expanded View [file EMBJ-42-e113150-s008.zip › Related to Figure EV4C.pdf]

Related to Fig 1B

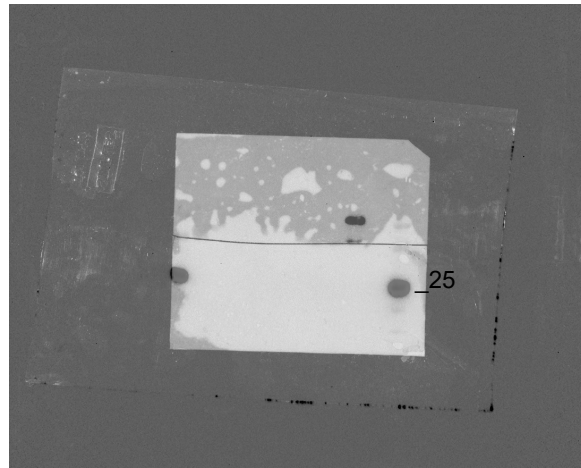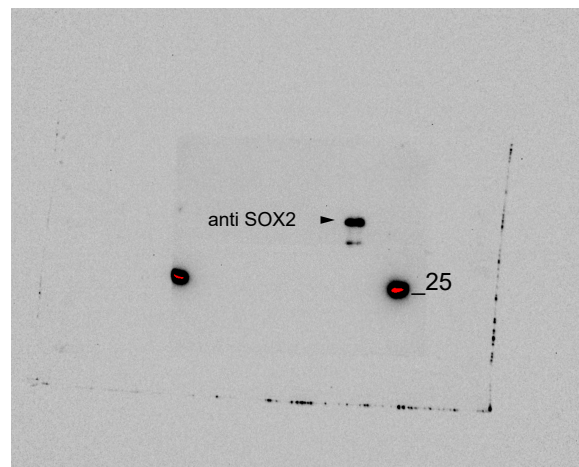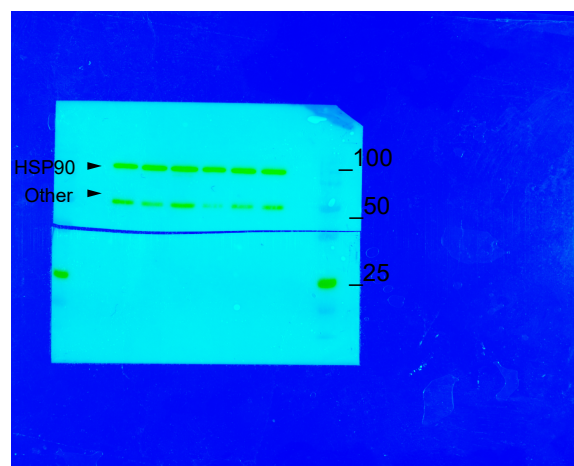

Supplement: Supplementary file 7 — Source Data for Figure 1 [file EMBJ-42-e113150-s007.pdf]
