# Supplementary material for: Pioneer activity distinguishes activating from non‐activating SOX2 binding sites
Source: EMBO J. 2023 Sep 11;42(20):e113150. doi: 10.15252/embj.2022113150 (PMC10577566; doi:10.15252/embj.2022113150)
Supplement: Supplementary file 6 — PDF+ [file EMBJ-42-e113150-s001.pdf]

Article

SOURCE DATA | TRANSPARENT PROCESS | OPEN ACCESS

THE EMBO JOURNAL

Pioneer activity distinguishes activating from non-activating SOX2 binding sites

Michela Maresca[1,†], Teun van den Brand[1,†], Hangpeng Li[2], Hans Teunissen[1], James Davies[2] & Elzo de Wit[1,*]

Abstract

Genome-wide transcriptional activity involves the binding of many transcription factors (TFs) to thousands of sites in the genome. Pioneer TFs are a class of TFs that maintain open chromatin and allow non-pioneer TFs access to their target sites. Determining which TF binding sites directly drive transcription remains a challenge. Here, we use acute protein depletion of the pioneer TF SOX2 to establish its functionality in maintaining chromatin accessibility. We show that thousands of accessible sites are lost within an hour of protein depletion, indicating rapid turnover of these sites in the absence of the pioneer factor. To understand the relationship with transcription, we performed nascent transcription analysis and found that open chromatin sites that are maintained by SOX2 are highly predictive of gene expression, in contrast to all other SOX2 binding sites. We use CRISPR-Cas9 genome editing in the Klf2 locus to functionally validate a predicted regulatory element. We conclude that the regulatory activity of SOX2 is exerted mainly at sites where it maintains accessibility and that other binding sites are largely dispensable for gene regulation.

Keywords acute protein depletion; chromatin accessibility; gene regulation; pioneer activity; transcription factors

Subject Categories Chromatin, Transcription & Genomics; Genetics, Gene Therapy & Genetic Disease

DOI 10.15252/embj.2022113150 | Received 25 November 2022 | Revised 17 July 2023 | Accepted 22 July 2023 | Published online 11 September 2023

The EMBO Journal (2023) 42: e113150

Introduction

In a given cell, gene expression is regulated by the concerted activity of hundreds of transcription factors (TFs) (Holmberg & Perlmann, 2012). Sequence-specific TFs enhance transcription by directly binding to DNA and promoting the recruitment of the transcriptional machinery (Grunstein, 1997; Ptashne & Gann, 1997; Allen & Taatjes, 2015; Isbel *et al*, 2022). Mammalian genomes encode over a thousand different TFs (Vaquerizas *et al*, 2009) and cell type specific TFs to a large degree determine cellular states. Therefore, delineating cell type specific activity of TFs is crucial to understanding organismal development and homeostasis. Discriminating functional TF binding sites, i.e., those that activate transcription, from non-functional sites that do not activate transcription, is a key challenge of the post-genomic era.

Classically, TF binding specificity has been characterized with different complementary methods. Proteins DNA interactions can be measured *in vitro* with methods such as SELEX, EMSA, or protein binding microarrays (Hellman & Fried, 2007; Stormo & Zhao, 2010). Alternatively, one can determine the binding sites of DNA binding factors in cellular context using chromatin immunoprecipitation (ChIP), which, when coupled to genome-wide read-outs (Hebbes *et al*, 1988; Solomon *et al*, 1988; Blat & Kleckner, 1999; Ren *et al*, 2000; Johnson *et al*, 2007; Robertson *et al*, 2007), enables their genome-wide identification. These methods result in the identification of tens of thousands of binding sites for a typical TF. For most sequence specific TFs, bound sites is substantially outnumbered by DNA sequences in the genome that correspond to the *in vitro* defined binding motif, indicating that these factors are not recruited to all motifs with equal efficiency. Furthermore, the same sequence-specific TF can have different binding profiles depending on the cell type (Lodato *et al*, 2013). The number of regulatory elements that control gene expression (i.e., functional elements) is again smaller than the number of bound sites (Todd *et al*, 2019).

One of the reasons why not all motifs for a given TF are bound is that the DNA is not accessible for binding by the factor in question. To make DNA elements accessible, a special class of TFs called pioneer factors is required (Zaret & Carroll, 2011; Zaret & Mango, 2016). Pioneer factors can bind to DNA wrapped around the histone octamer. Upon binding, these factors can either unwrap nucleosome on their own (Cirillo *et al*, 2002) or recruit chromatin remodeling complexes (Swinstead *et al*, 2016) resulting in the formation of nucleosome free, accessible DNA. These genomic regions can then be bound by non-pioneer TFs to activate gene expression. During differentiation, the transition from one cell type to another is driven by the formation of cell type specific accessible regions, which, in turn, activate cell type specific genes. As such, activity of pioneer factors often represents the first step in cell fate commitment

1 Division of Gene Regulation, The Netherlands Cancer Institute, Amsterdam, The Netherlands
2 MRC Molecular Haematology Unit, MRC Weatherall Institute of Molecular Medicine, Radcliffe Department of Medicine, University of Oxford, Oxford, UK
*Corresponding author. Tel: +31 20 512 9111; E-mail: e.d.wit@nki.nl
†These authors contributed equally to this work

© 2023 The Authors. Published under the terms of the CC BY 4.0 license

(Zaret & Mango, 2016). Reprogramming of somatic cells to induced pluripotent stem cells is also preceded by the induction of pluripotency associated accessible DNA elements (Li *et al*, 2017, 2021). It is therefore not surprising that three out of four of the original Yamanaka reprogramming factors are pioneer transcription factors (i.e., OCT4, SOX2 and KLF4) (Takahashi & Yamanaka, 2006; Iwafuchi-Doi & Zaret, 2014; Jerabek *et al*, 2014; Soufi *et al*, 2015; Roberts *et al*, 2021). In addition, knock-out of OCT4 leads to a loss of accessible sites (King & Klose, 2017), further emphasizing its role as pioneer factor.

Chromatin accessibility, which can be measured with ATACseq (Buenrostro *et al*, 2013) or DNAse I hypersensitivity mapping (Boyle *et al*, 2008), clearly correlates with gene expression changes at selected loci (see e.g., Isoda *et al*, 2017; Pijuan-Sala *et al*, 2020). However, identifying the exact sites that drive the expression changes genome-wide remains difficult. For instance, experiments that aimed to link accessibility changes to changes in mRNA levels in a breast cancer cell line following treatment with retinoic acid or TGF-β for 72 h found only a meager correlation (preprint: Kiani *et al*, 2022). Loss-of-function studies for TFs suffer from a related complication. During conditional knock-out or knock-down experiments, the protein level is passively decreased through cell division or the rate of degradation, which means it can take many hours before the protein is completely depleted. Because loss of pluripotency TFs can induce differentiation before the TF is completely depleted it can be difficult to separate direct from indirect effects. In recent years, acute protein depletion methods have been developed which can ablate proteins directly (Verma *et al*, 2020). These methods typically work by using a small molecule to target the protein of interest to an E3 ligase complex resulting in ubiquitination followed by proteasomal degradation. This results in rapid, synchronous, and near complete loss of a protein.

In this work, we used acute depletion of SOX2 to characterize the dynamics of accessible regions created by pioneer factors and the relationship to gene expression. Acute loss of SOX2 in mouse embryonic stem cells (mESCs) reveals the loss of thousands of accessible regions in less than 2 h. When we link these accessibility changes following SOX2 depletion to changes in nascent transcription, we find that differentially accessible regions are strongly predictive for changes in gene expression. Importantly, binding sites of SOX2 that are not associated with changes in accessibility have limited predictive power for changes in gene expression. Our experiments establish the importance of chromatin accessibility created and continuously maintained by pioneer transcription factors in the regulation of gene expression.

Results

Continuous pioneering activity of transcription factors is necessary for maintenance of accessible chromatin

To determine the direct consequences of TF loss at high temporal resolution, we employed the degradation tag (dTAG) system to induce rapid and acute protein depletion (Nabet *et al*, 2018). We previously performed C-terminal tagging of the *Sox2* and *Nanog* genes with the *FKBP*^{F36V} sequence using CRISPR/Cas9 genome editing in mouse embryonic stem cells (mESC) (Liu *et al*, 2020). The heterobifunctional molecule dTAG-13 binds to the FKBP^{F36V} domain and targets the fusion protein for proteasomal degradation (Fig 1A). Immunoblotting shows that treatment with dTAG efficiently degrades tagged SOX2 protein within 1 h (Fig 1B). To determine whether dTAG treatment is uniquely selective for SOX2, we performed quantitative mass spectrometry-based proteomics after 30 min of protein depletion (Fig 1C). After 30 min of dTAG treatment, the only protein that showed significant changes was SOX2. These results show that acute protein depletion of SOX2 is fast and highly specific.

To determine the direct effects on accessibility following SOX2 loss we performed ATACseq (Buenrostro *et al*, 2013). In ATACseq the bacterial transposase Tn5 tagments DNA that is accessible enabling the detection of open chromatin regions (OCRs). We performed an ATACseq time course experiment at 30 min, 1, 2, 6, and 24 h following SOX2 depletion. The genomic region surrounding the *Ctgf* locus is exemplary for the consequences of acute SOX2 loss on the chromatin accessibility landscape (Fig 1D). An OCR ~4.5 kb upstream from the *Ctgf* TSS starts losing accessibility already after 2 h of SOX2 depletion. To catalog the genome-wide changes upon SOX2 depletion, we perform peak calling using MACS2, which detected 169,050 peaks on the combined time series. After filtering, we called differentially accessible regions (DARs) on 157,839 peaks using DESeq2 (see Materials and Methods for details). SOX2 depletion results in loss of accessibility at thousands of regions already at 2 h post depletion (hpd) (n = 6,365) (Fig 1E), in line with its role as a pioneer factor. SOX2 forms a heterodimer with OCT4, which is thought to form the initial binding event that facilitates nucleosome removal (Ambrosetti *et al*, 1997; Kumar Mistri *et al*, 2011; Michael *et al*, 2020). To test whether acute loss of OCT4 also results in the rapid loss of accessible sites we performed ATACseq in an OCT4 degron line (Boija *et al*, 2018). We found that accessible sites were lost with roughly similar dynamics (Fig EV1A and B). When we deplete the non-pioneer factor NANOG we see hardly any changes in accessible chromatin (Fig EV1A and B). The surprisingly fast loss of accessible chromatin sites in the absence of pioneer TFs suggests continuous pioneer activity is required for the maintenance of these OCRs.

To determine whether the loss of accessibility is reversible, we made use of a feature of degrons that enables the reconstitution of the depleted protein. By washing out the dTAG molecule, SOX2 is no longer degraded. We measured SOX2 protein levels at different time points after dTAG washout and could only observe levels comparable to non-depletion after 24 h (Fig EV1C). To determine whether there is a re-establishment of OCRs following SOX2 re-expression, we performed 2 h of SOX2 depletion followed by 24 h of dTAG washout and performed ATACseq. We found that after 24 h of washout, there is a near complete restoration of accessibility at DARs (Fig 1F), indicating the reversibility of the phenotype.

We next dissected the functional domain of SOX2 protein that is necessary to mediate chromatin accessibility. SOX2 binds DNA with its N-terminal high-mobility group (HMG) domain. Interaction of SOX2 with other proteins is promoted mostly by its trans-activation domain (TAD) at the C-terminus (Cox *et al*, 2010). Our degron lines offer an opportunity to investigate the role of the different protein domains in the opening of chromatin in their native chromatin environment in living cells. We transfected SOX2 degron cells with plasmids encoding full-length or truncated versions of the SOX2 protein fused to GFP, or GFP alone as a negative control (Fig EV1D and E).

 © 2023 The Authors

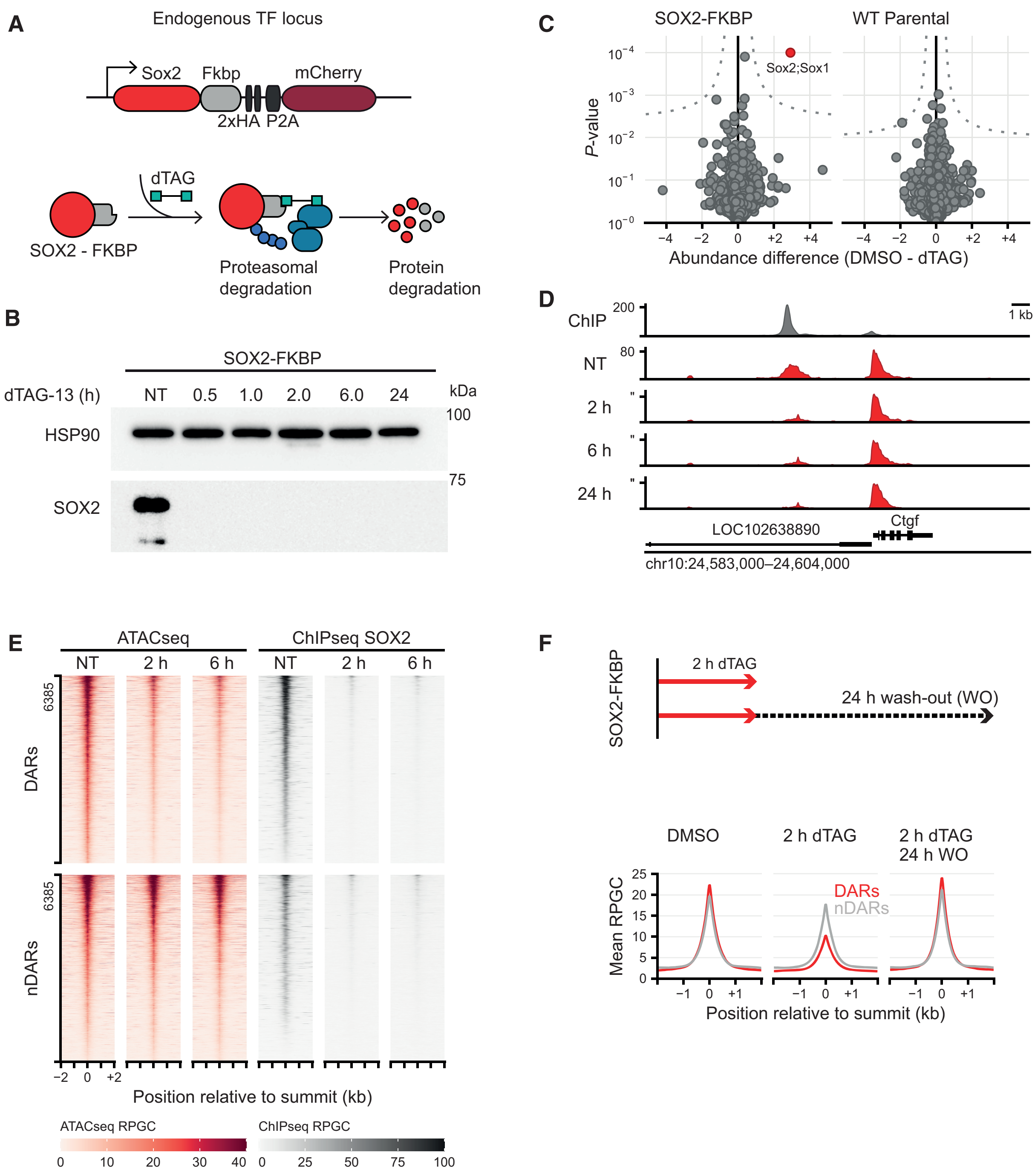

Figure 1. Rapid depletion of SOX2 and OCT4 affects the accessibility landscape of thousands of sites.

A Schematic representation of the dTAG system, wherein an FKBP-tagged protein can be proteasomally degraded upon addition of the dTAG-13 small molecule.

B Western blot showing protein abundance of SOX2 upon addition of dTAG-13 for the indicated times in SOX2-FKBP cell lines. HSP90 was used as loading control (NT, Not Treated).

C Quantitative mass spectrometry results showing the differential protein abundance upon 30 min of dTAG treatment versus DMSO treated SOX2-FKBP cells and parental (untagged) cells.

D Genomic tracks showing SOX2 binding by ChIPseq (gray) and accessibility by ATACseq (red) around the *Ctgf* gene for SOX2-FKBP cell line after indicated times of depletion by dTAG treatment. *Y*-axes show reads per genomic content (RPGC).

E Heatmap showing accessibility and SOX2 ChIPseq before and after dTAG treatment in SOX2-FKBP cell line at SOX2 peaks that are differentially accessible regions (DAR) or where no differentially accessible region is detected (nDAR) partially matched for SOX2 binding.

F Top: Experimental procedure for SOX2 ATACseq after wash-off of dTAG. Bottom: Average profile of ATACseq in SOX2 degradation system and restoration of SOX2 after 2 and 24 h of dTAG wash-off at the same DARs/nDARs as in (E).

Source data are available online for this figure.

We then treated the transfected cells with dTAG to deplete the endogenous SOX2 protein or DMSO as a control and performed ATACseq on the sorted GFP positive cells. As expected, expression of the GFP protein alone resulted in a loss of accessibility at DARs we find in the untransfected cells following SOX2 depletion (Fig EV1E). On the other hand, exogenous expression of the full-length SOX2-GFP fusion did not show dramatic loss of accessibility at DARs in the absence of endogenous SOX2, indicating that a SOX2-GFP fusion protein can exhibit pioneering activity similar to endogenous SOX2 (Fig EV1F). Cells expressing either SOX2-HMG-GFP or SOX2-TAD-GFP do not rescue accessibility at DARs following SOX2 depletion. Our results show that the SOX2 HMG is not enough to promote the formation of accessible sites, rather it is the concerted action of both the HMG and the TAD that is required to maintain accessibility.

To determine how DARs relate to SOX2 binding, we performed ChIPseq with an antibody to the HA-tag in the SOX2 fusion protein in absence and presence of the dTAG-13 degrader. To our surprise, a sizable fraction of SOX2 bound regions did not lose accessibility (non-DAR: nDAR) (Figs 1E and EV2A). We performed ChIPseq of HA-tagged SOX2 at 2 and 6 hpd to show that the nDARs are not a consequence of residual binding of SOX2 (Fig 1E). Furthermore, motif analysis shows that regions bound by SOX2 and both losing or retaining accessibility, show an enrichment of SOX2 motifs over background levels (Fig EV2B). Many of these sites are accessible, but the retention of accessibility following SOX2 depletion shows that SOX2 is not uniquely responsible for the maintenance of these OCRs. In conclusion, there are different categories of SOX2 binding sites, those where SOX2 shows pioneer activity and those where SOX2 has no pioneer activity or is not the sole pioneering factor.

To understand what determines a DAR from a nDAR we analyzed the protein composition at these sites using a compendium of publicly available ChIPseq data in mESCs (i.e., Cistrome, Dataset EV1) (Zheng *et al*, 2019). We reasoned that important factors would be predictive of these sites, so we aimed to discriminate DARs from a control set of nDARs with partially matched SOX2 binding levels using random forest classification. After filtering, we considered 233 unique DNA binding factors as predictors. As a performance metric, these factors reached an area under the receiver operator characteristic (ROC) curve (AUC) of 0.79. Among the top scoring factors we find OCT4 and NANOG, but also ZFP57 and the BAF (a.k.a. mSWI/SNF) complex member SS18 (King & Klose, 2017), which showed specific binding at DARs (Fig EV2D). While SOX2-bound regions that do not change accessibility are preferentially bound by the transcription factors TFAP2C (Pastor *et al*, 2018) and CTCF (Fig EV2E) (King & Klose, 2017). Next, we wanted to gain insight into the specific histone modifications that are predictive for DARs, so we performed random forest analysis on 38 histone modification ChIPseq datasets, which reached an AUROC of 0.66. Interestingly, among the histone modifications that are most important in the prediction of differential accessibility are the non-canonical histone modifications H2BK20ac (Kumar *et al*, 2016) and H3K64ac (Di Cerbo *et al*, 2014) (Fig EV2D). We also overlapped the DAR and nDAR set with published ChromHMM states (Pintacuda *et al*, 2017) and found that the DARs are enriched for the "enhancer" state, whereas the matched nDARs are also enriched for the "active promoter" state (Fig EV2F). This is also consistent with the H3K79me2 levels enriched on the nDARs (Fig EV2E). It is important to reiterate that like DARs, these matched nDARs generally have SOX2 binding.

SOX2 and OCT4 act both as partners and independently to maintain accessibility

In pluripotent cells, OCT4 and SOX2 often occupy the same genomic sites (Chen *et al*, 2008; Marson *et al*, 2008). Furthermore, Cryo-EM structures of the DNA binding domains in complex with nucleosomes have revealed that OCT4 and SOX2 form a heterodimer that distorts the interface between DNA and the nucleosome, which may promote the formation of an accessible site (Dodonova *et al*, 2020; Michael *et al*, 2020). The rapid and specific depletion of OCT4 and SOX2 enables us to determine the contribution of either factor to the formation of OCRs in a cellular context. To this end, we investigated the DARs at 2 hpd in more detail. A simple stratification revealed sites that lose accessibility following depletion of either OCT4 or SOX2 (Common DARs) and thus depend on the action of both proteins (Fig 2A, left panel). On the other hand, we observed a sizable number of peaks that are exclusively lost following depletion of SOX2 (SOX2 DARs) or OCT4 (OCT4 DARs), but not in both, making these uniquely dependent on either factor (Fig 2A, middle and right panel). Depletion of the non-pioneer factor NANOG has no consequences on any of these DARs.

After quantifying the peaks responding to both SOX2 and OCT4 depletions we found 3,168 common DARs depend on both SOX2 and OCT4 for their accessibility, whereas we found 3,497 SOX2 DARs and 3,205 OCT4 DARs (Fig 2B). To understand the differences between these three groups of sites we performed motif analysis. We found that the common DARs were most strongly enriched for the Pou5f1::Sox2 compound motif, consistent with the co-dependency of these proteins. The SOX2 DARs on the other hand were enriched for SOX-family motifs, but not for many POU-family motifs. For the OCT4 DARs this was actually reversed (Fig 2C). When we performed a more granular analysis incorporating the relative strengths of effect sizes on the accessibility we could recapitulate these observations (Appendix Fig S1A and B). Moreover, this revealed that the least OCT4 dependent SOX2 DARs have no enrichment for the Pou5f1::Sox2 compound motif, whereas the least SOX2 dependent OCT4 DARs retained this enrichment.

To determine the contribution of SOX2 and OCT4 binding to accessibility, we performed ChIPseq of OCT4 in the SOX2-FKBP line and of SOX2 in the OCT4-FKBP line (Fig 2D). We find that all DARs we identified, regardless of their specificity to SOX2 and OCT4 depletion, are bound by both SOX2 and OCT4 together. Upon depletion of the tagged TF, the ChIPseq signal of the tagged TF disappears. Generally, we also find that the signal of the partner TF at DARs is greatly reduced by, suggesting that OCT4 and SOX2 stabilize each other's binding affinity to chromatin. The exception to the general case is that the SOX2 DARs after OCT4 depletion only mildly decreased in SOX2 binding intensity (27%), indicating that SOX2 is able to maintain accessibility at these sites independent of OCT4. Conversely, OCT4 binding intensity is reduced by 66% at OCT4 DARs after SOX2 depletion, hinting that accessibility is maintained here through alternative means. These results demonstrate that despite the co-occupation of SOX2 and OCT4 proteins at most DARs, accessibility changes upon pioneer TF depletion are not solely encoded by binding patterns in non-treated conditions.

 © 2023 The Authors

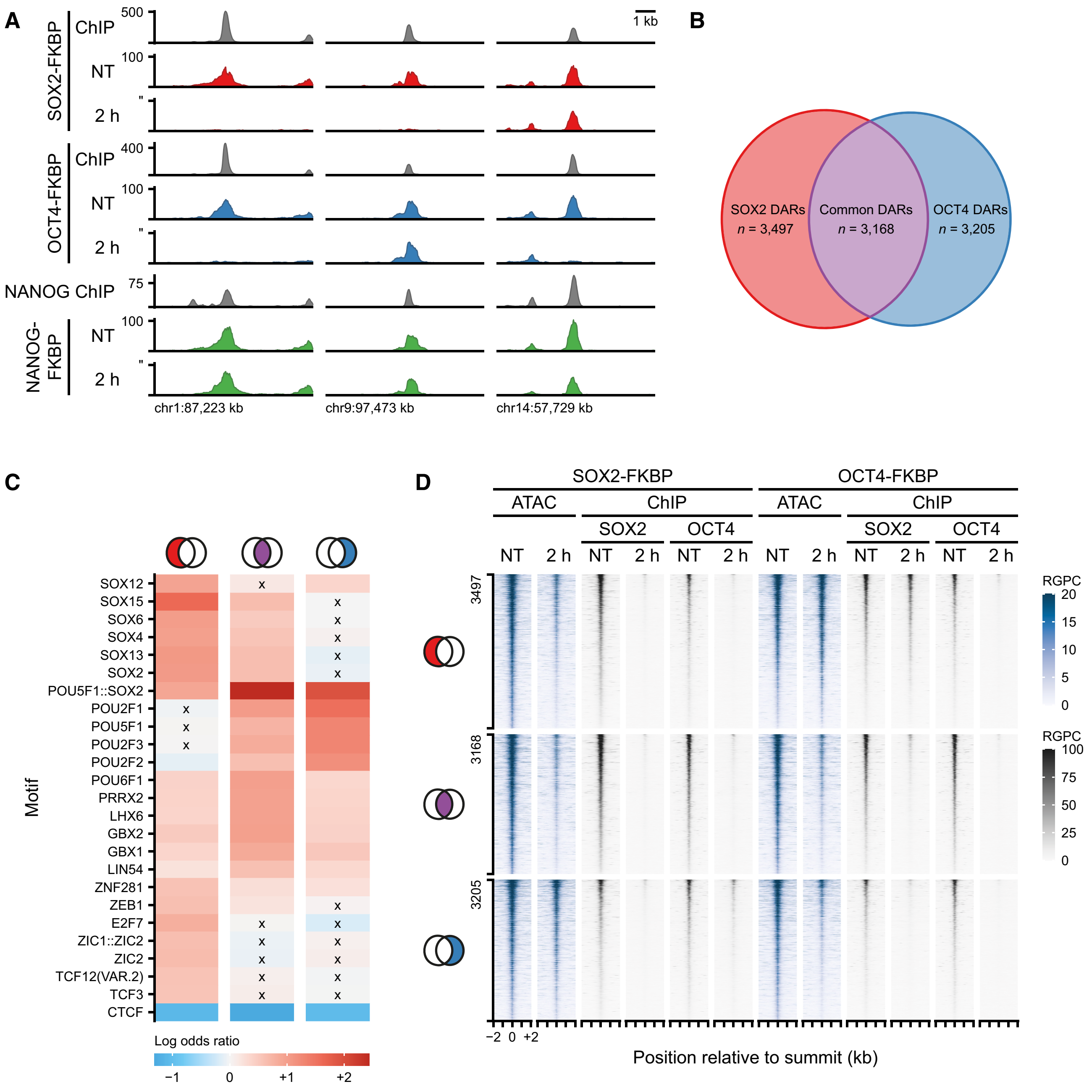

Figure 2. Loss of SOX2 and OCT4 effect accessibility at shared and independent regions.

A Example regions of changes in accessibility as measured by ATACseq during the SOX2, OCT4, and NANOG depletion time course. *Y*-axis indicates reads per genomic content (RPGC). In gray are shown the ChIPseq tracks for SOX2, OCT4, and NANOG.

B Euler diagram shows the number of regions losing accessibility (DARs) after SOX2 depletion only (red, SOX2 DARs), OCT4 depletion only (blue, OCT4 DARs) and in common (purple, common DARs).

C Heatmap showing the top 25 motifs at SOX2, common and OCT4 DARs. Colorbar indicates odds of finding the motif in the DAR set relative to all other OCRs. Crosses denote non-significant odds.

D Tornado plots showing ATACseq (blue) after SOX2 and OCT4 depletion. Regions are divided by SOX2, Common and OCT4 DARs. In gray, ChIPseq signal of SOX2 and OCT4 after SOX2 and OCT4 depletion. RPGC: reads per genomic content.

SOX2 directly controls accessibility with a sub-hour time resolution

Our high-resolution time series allows us to investigate differences in the temporal dynamics of OCRs following SOX2 depletion. Upon visual inspection it became clear that sites lose accessibility at different rates (Fig 3A). For a subset of sites, no residual accessibility can be detected as soon as 30 min after depletion, whereas other sites take more than 2 h to become fully inaccessible. Quantification of the DARs at every time point shows a gradual loss of OCRs over

© 2023 The Authors

time (Fig 3B). The initial gain of accessibility in OCRs following SOX2 depletion is very limited (less than 1,000 sites in the first 6 hpd), and only at 24 hpd thousands of OCR have increased their accessibility.

Our temporal resolution is sufficient to calculate parameters for the dynamics of each individual DAR. To this end we fitted a log-logistic curve describing the decrease or increase in accessibility. From these curves we can estimate for every site the moment

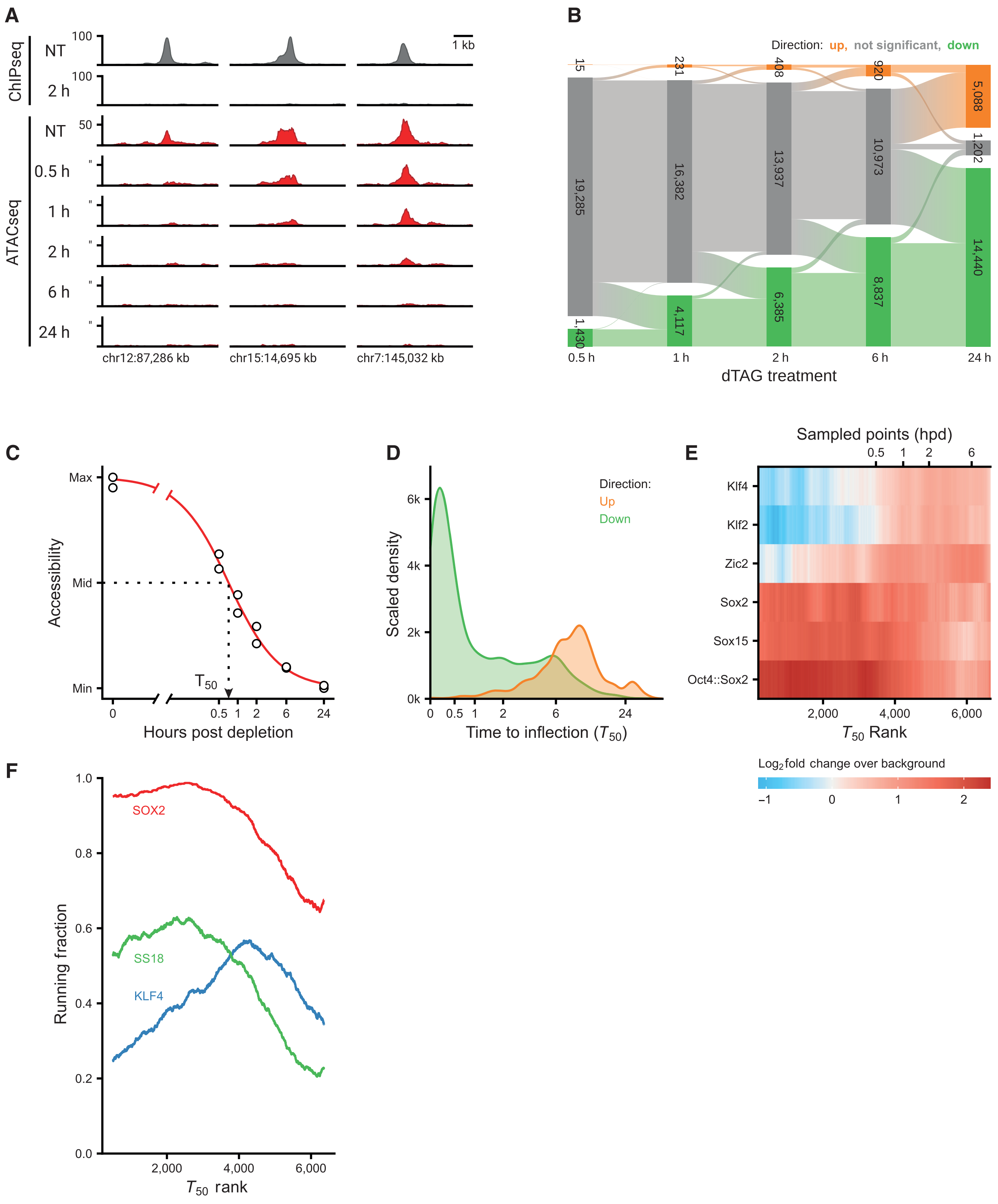

Figure 3.

 © 2023 The Authors

Figure 3. Loss of SOX2 affect chromatin accessibility within sub-hour time resolution.

A Example regions of changes in SOX2 occupancy measured by ChIPseq (to, in gray) and in accessibility as measured by ATACseq (red) during the SOX2 depletion time course. *Y*-axis indicates reads per genomic content (RGPC).
B Alluvial diagram shows the number of regions gaining accessibility (up, orange) or losing accessibility (down, green) over the SOX2 depletion time course.
C Illustration of estimating the time to inflection (T_{50}) for a DAR, wherein a log-logistic model is fit to the data over time after depletion.
D Kernel density estimate of the time to inflection for DARs going up and down. The density is scaled such that the area under the curve integrates to the number of DARs. Time to inflection is displayed with inverse hyperbolic sine transformation.
E Heatmap of motif enrichment displaying downregulated DARs on the *X*-axis sorted by time to inflection. Colors display a fold change of a centered running mean, measuring the fraction of peaks containing the indicated motifs over a 400 peaks window, relative to the fraction of nDARs containing these motifs. Sampled time points are indicated at the bins where the time to inflection surpasses the sampled time.
F Patterns of protein binding at sites ordered by their inflection time (T_{50}). Running fraction represents the fraction of peaks in a 1,000-peak window that overlaps one of the specified ChIPseq datasets. The SS18 and KLF4 peaks were acquired from Cistrome, whereas the SOX2 peaks were taken from the SOX2-FKBP line in the untreated condition.

following depletion at which there is a half-maximal change in accessibility: a value we refer to as T_{50} (Fig 3C). The distributions of the T_{50} values for increases and decreases in accessibility (Fig 3D) confirm the analysis of significantly changed peaks (Fig 3B) that losses in accessibility are fast (majority of T_{50} reached before 30 min after depletion) and increases are slow (the majority $>$ 6 hpd). The T_{50} values enabled us to rank the peaks according to the speed at which they lose accessibility. When we determined the motif enrichment as a function of the T_{50} rank we found that the most rapidly changing peaks are enriched for motifs containing the SOX2 consensus motif (Fig 3E). Peaks that rank later according to their T_{50} values show enrichment for KLF2/4 and ZIC2, which could mean that these accessible sites are not solely regulated by SOX2. To investigate this we used publicly available ChIPseq data (Zheng *et al*, 2019) to determine the protein composition at the DARs according to their T_{50}. As expected, SOX2 binding was strongly enriched in the sites that are lost most quickly, but is less enriched in the sites that take more time to change. This pattern is closely followed by the mSWI/SNF subunit SS18, suggesting that this chromatin remodeling complex collaborates with SOX2 to create these open chromatin sites (Fig 3F). Interestingly, consistent with our motif enrichment analysis, sites that lose accessibility later in the time course are more often bound by KLF4. These analyses show that studying accessibility changes at high temporal resolution can help to predict which proteins are involved in the cascade of regulatory changes.

Changes in chromatin accessibility are directly related to transcriptional changes

As a transcription factor, SOX2 is responsible for maintaining transcription of genes associated with the pluripotent state. Because RNAseq measures the stable transcript pool, rapid changes in transcription may be occluded by the total level of mRNA. Therefore, to determine direct effects on transcription we measured the production of nascent transcripts using TT_{chem}seq (Gregersen *et al*, 2020). In TT_{chem}seq, nascent transcripts are pulse-labeled with a nucleotide analog, allowing the detection of transcription from both genic and intergenic sequences. Already after 30 min of SOX2 depletion we find that hundreds of transcripts are significantly de-regulated (Fig 4A). An example of a gene that is immediately lost following SOX2 depletion is the non-coding pluripotency transcript *Platr11* (Fig 4B and C). Upon closer examination we found that accessibility surrounding the (empirical) TSS of this transcript is also severely diminished following SOX2 depletion (Fig 4C). These results are consistent with SOX2 controlling chromatin accessibility to promote the expression of its target genes. It should be noted that, in contrast to the nearly unidirectional DARs, a balanced number of genes show a decrease and increase in transcription upon SOX2 depletion (Fig 4A). An example of an upregulated gene is *Suv39h1*, which is associated with the downregulation of an anti-sense non-coding transcript coming from the opposite strand. The TSS of this antisense transcript is associated with an open chromatin region that is lost upon SOX2 depletion (Fig 4D). A similar trend is observed for *Top1* (Fig EV3A), suggesting that SOX2 regulating an anti-sense transcripts may be a mechanism for repressing protein coding genes. Interestingly, we also find a subset of genes that show transient upregulation (Figs 4A and B, and EV3B). Because there is a general lack of DARs that show an increase in accessibility, we perform motif analysis at all OCRs in the 40 kb region centered at the TSS of downregulated genes and find a mild enrichment for OCT4::SOX2 and SOX family motif (Fig EV3C). For upregulated genes, a similarly mild enrichment for nuclear receptor family motifs (e.g., ESRRB) is observed, but not SOX2. This indicates that upregulation cannot be explained a direct role of SOX2 as repressor. Pathway analysis on differentially expressed genes shows a transient enrichment of cholesterol biosynthesis genes in the early time points (Fig EV3D), wherein *Hmgcs1* is a member (Fig EV3B). Intriguingly, we do not find any pathway enriched among the downregulated genes.

To systematically determine the relationship between changes in chromatin accessibility and gene expression we aligned the positions of DARs to the TSSs of differentially expressed genes at 2 hpd compared to a set of (expression matched) control genes that do not change expression. We found a strong enrichment of DARs around the TSSs of downregulated genes which was not observed for stable nDARs that do not change accessibility (Fig 5A). We did not find a similar enrichment for upregulated genes, where downregulated DARs are mildly depleted (Fig EV4A). In order to identify the most critical factors that determine which genes are activated by SOX2, we aimed to predict which genes go down in expression upon SOX2 depletion in comparison to an expression matched control set. In absence of a precise enhancer-target map, we exploited the pattern of DAR enrichment around TSSs of downregulated genes for making predictions. Specifically, we used logistic regression using the gene outcome (downregulated or undetectable change) as dependent variable and the counts (or sum of weights, Fig 5B) of different categories of nearby genomic loci as predictors. To ensure our prediction model is not dependent on a single set of control genes we

© 2023 The Authors

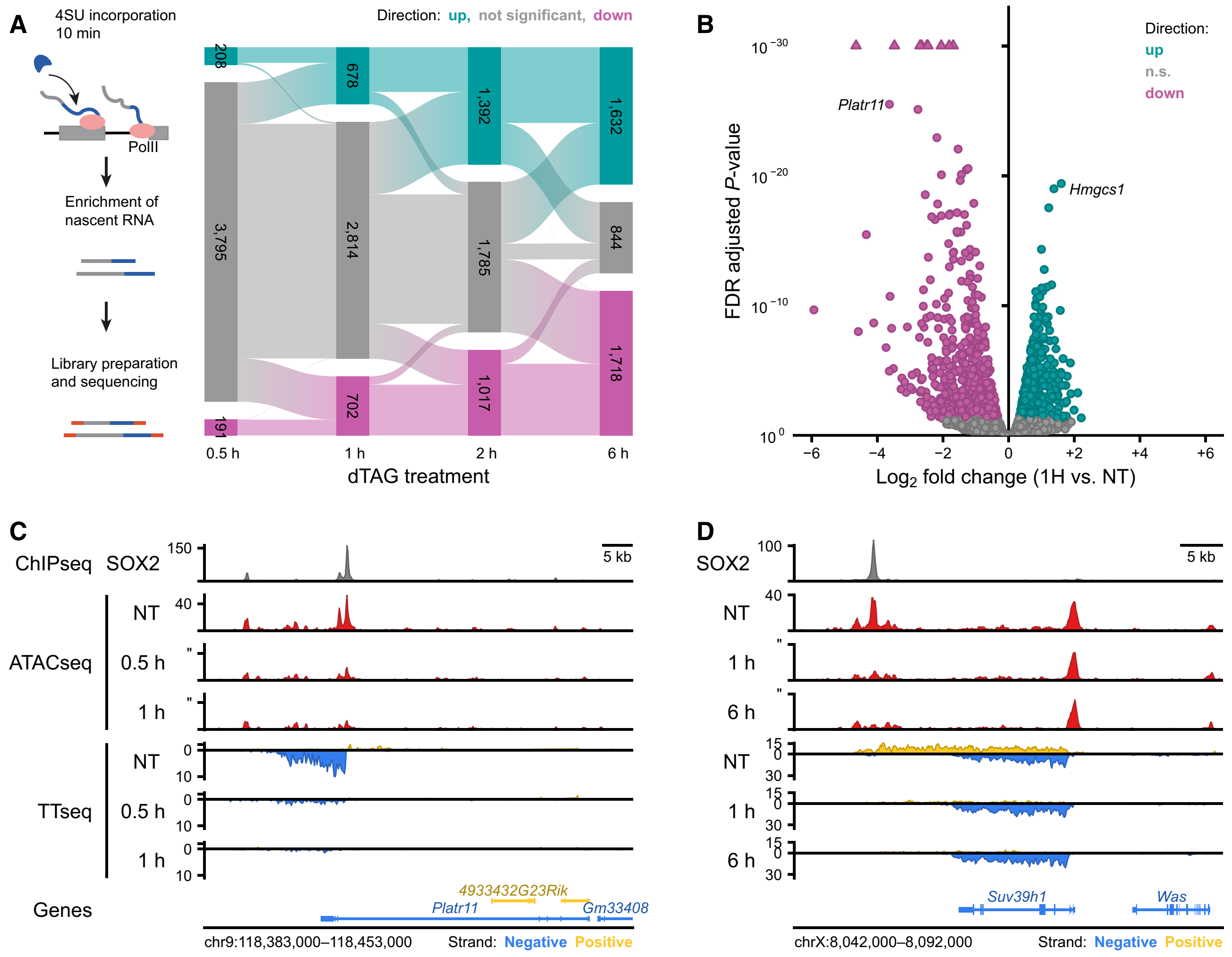

Figure 4. Direct effects of SOX2 depletion on transcription.

A Top: Schematic representation of the TT_{chem}seq method to measure nascent transcripts. Bottom: Alluvial diagram showing the differentially transcribed units: upregulated units (up, light blue) and downregulated units (down, violet) during SOX2 depletion time course.

B Volcano plot showing effect sizes and significance of the downregulated (violet) and upregulated (lightblue) transcribed units after 1 h of SOX2 degradation. n.s.: not significant.

C Genomic tracks showing SOX2 ChIPseq (top), ATACseq data (middle) and nascent transcription measured with TT_{chem}seq (bottom) at the *Platr11* locus in untreated (NT) 0.5 and 1 h dTAG-13 treated SOX2-FKBP cells.

D The same as in (C) but for *Suv39h1* locus in untreated (NT), 1 and 6 h dTAG-13 treated.

generated 100 samples of expression matched genes. We assessed a variety of association rules for predictive performance and found that setting a simple distance threshold from the transcription start site outperforms more complex association rules (Fig EV4B). Reasoning that counting peaks using a distance threshold is equivalent to weighting the peaks according to a uniform kernel centered at the TSS, we next explored whether using other kernel functions as weighting schemes could improve prediction (Fig EV4C). The heavy-tailed Laplace or Cauchy probability density functions (PDF) yielded the best predictions and we continued with the Cauchy weighting for further predictions. In the end, we achieved an area under the receiver operator characteristic curve (AUC) of 0.82 (Figs 5C and EV4C) in predicting downregulated genes, demonstrating a tight connection between loss of chromatin accessibility and downregulation of transcription.

Since SOX2 is crucial in maintaining a subset of OCRs, we wanted to test how differential accessibility compares to SOX2 occupancy in predicting gene expression changes. To test this, we included our SOX2 ChIPseq data (Marson *et al*, 2008) in our analyses (Fig 5C). Importantly, solely using SOX2 occupancy as predictor performs noticeably worse, showing that accessibility changes are better predictors for genes regulated by SOX2 than occupancy of SOX2 (Fig 5C). Note that this poorer performance is not particular to this specific dataset we created, rather, this is the case for all publicly available SOX2 ChIPseq datasets in mouse embryonic stem cells that we have tested (Fig EV4D). Next, we analyzed how well genomic regions where SOX2 displays no pioneer activity, i.e., bound by SOX2, but which show no change in accessibility following SOX2 depletion, could predict expression changes (Fig 5C). These regions have an even lower predictive power for changes in

 © 2023 The Authors

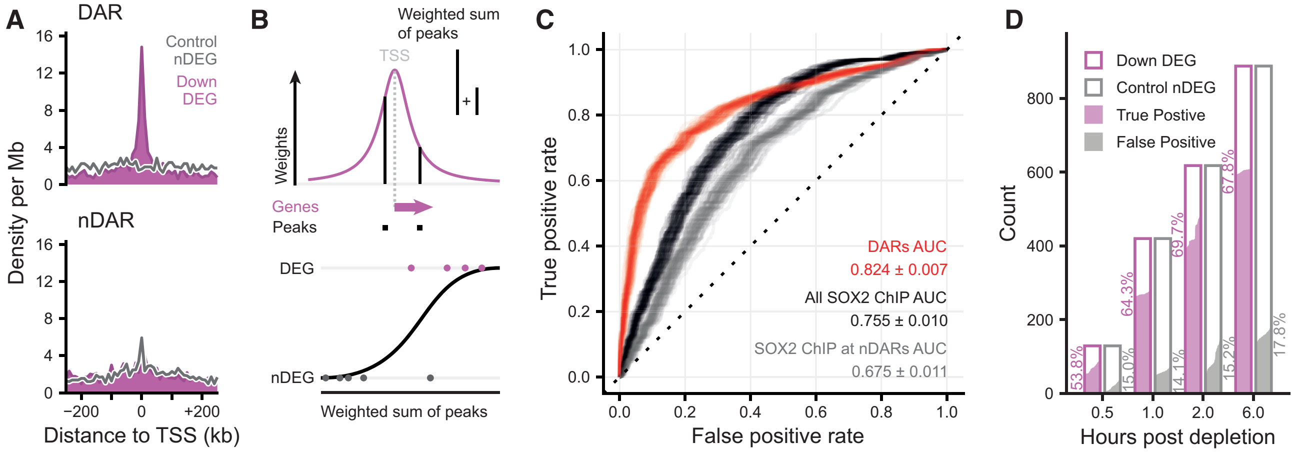

Figure 5. Open chromatin regions maintained by SOX2 are associated with transcription.

A Density of peaks in 10 kb bins nearby transcription start sites (TSS) of downregulated (purple) differentially expressed genes (DEGs) and expression-matched stable control (gray) non-DEGs (nDEGs). Panels indicate the set of peaks that were aligned to the TSS: differentially accessible regions (DARs) or stable non-DARs (nDAR).

B Schematic illustration of taking the weighted sum of peaks per gene, weighted by a kernel function (top) followed by logistic regression predicting the differential expression status of a gene (bottom).

C Receiver operator characteristic (ROC) curves indicating the predictive strength on differential expression status of downregulated DEGs versus matched control nDEG, for various sets of predictors. The "DARs" set of predictors use peak categories of ATACseq data. The "All SOX2 ChIP" set has weighted sums of SOX2 ChIPseq peaks. The "SOX2 ChIP at nDAR" has weighted sums of the SOX2 peaks that do not overlap DARs. Different transparent lines indicate 100 re-samplings of the expression-matched nDEGs.

D Barplot showing the number of downregulated DEG (case) and nDEG (control) genes identified as true or false positive in the predictions with the "DARs" features at the indicated time points. Predictions were taken from ROC curves at the threshold of maximum accuracy. Filled area indicates complementary cumulative distribution function of counts under 100 re-samplings of the expression-matched nDEGs. Percentages indicate the average true and false positive rates of the different samplings.

gene expression (Figs 5C and EV4D). From these results, we conclude that the genomic regions where SOX2 acts as a dominant pioneer factor are crucial for SOX2 mediated gene activation. The sites that are bound by SOX2, but where it exerts no pioneer activity contribute to gene regulation in a much diminished manner.

Next, we decided to determine the predictive power of accessibility changes at different timepoints. Data derived from 2 hpd samples were most predictable, wherein we can correctly predict downregulation of about 70% of genes on average, at the expense of incorrectly flagging about 14% of non-DEGs as downregulated (Fig 5D). We suspect that prior to 2 h the prediction is hindered by the time it takes to establish and detect the primary effects, whereas after 2 h secondary effects may start to weaken the causal linkage between accessibility and transcription. These results show that TF depletion followed by nascent transcription mapping is a powerful method to prioritize direct from indirect regulatory targets of a TF. Furthermore, our results show that chromatin accessibility is tightly linked to transcription and, in fact, differential accessibility has more predictive power than ChIPseq data with regard to which sites drive gene expression.

Disruption of a SOX2 dependent OCR confirms prediction of a putative regulatory element

We have demonstrated that SOX2 depletion alters the transcription of many genes and that changes in chromatin accessibility can predict which genes respond to SOX2 depletion. Although this strongly suggests that these accessible regions directly control transcription of their putative target genes, the results remain correlative. Based on our prediction model the gene that has the highest probability of being affected by SOX2 depletion is *Klf2*. Correspondingly, we find that *Klf2* transcription is strongly reduced, already after 30 min (Fig 6A). Flanking the TSS of *Klf2* there are two OCRs that rapidly lose accessibility after SOX2 depletion. One is 3 kb upstream from the TSS and the second is 15 kb downstream of the TSS; both are bound by SOX2. Distal regulatory elements are thought to regulate genes through physical proximity within the space of the nucleus (De Laat & Duboule, 2013). To determine whether the regulatory elements around the *Klf2* gene are interacting with the *Klf2* promoter, we made use of Micro-Capture-C (MCC), a basepair resolution chromosome conformation method (Hua *et al*, 2021). Our analyses show that both the proximal and the distal enhancer interact with the *Klf2* promoter (Fig 6A), further suggesting that these elements are functional in driving *Klf2* gene expression.

In order to demonstrate that our prediction of functional regulatory elements based on differential ATACseq and TT_{chem}seq is correct, we wanted to genetically address the causality. We used CRISPR-Cas9 coupled with homology directed repair (HDR) to mutate the core SOX2 motif within the DAR region 15 kb downstream of *Klf2*. The mutation is expected to impair SOX2 binding, while keeping the surrounding DNA sequence intact. We selected 5 clones homozygous for the motif mutant and measured *Klf2* expression levels by qPCR (Figs 6B and Fig EV5A and B). We find that mutation in the SOX2 motif leads to a ~45% downregulation of the *Klf2* gene. To determine whether there is additional signal present in the regulatory element we decided to also disrupt the surrounding sequence. We used CRISPR-Cas9 genome editing to homozygously disrupt the downstream putative regulatory element (Fig EV5C and

© 2023 The Authors

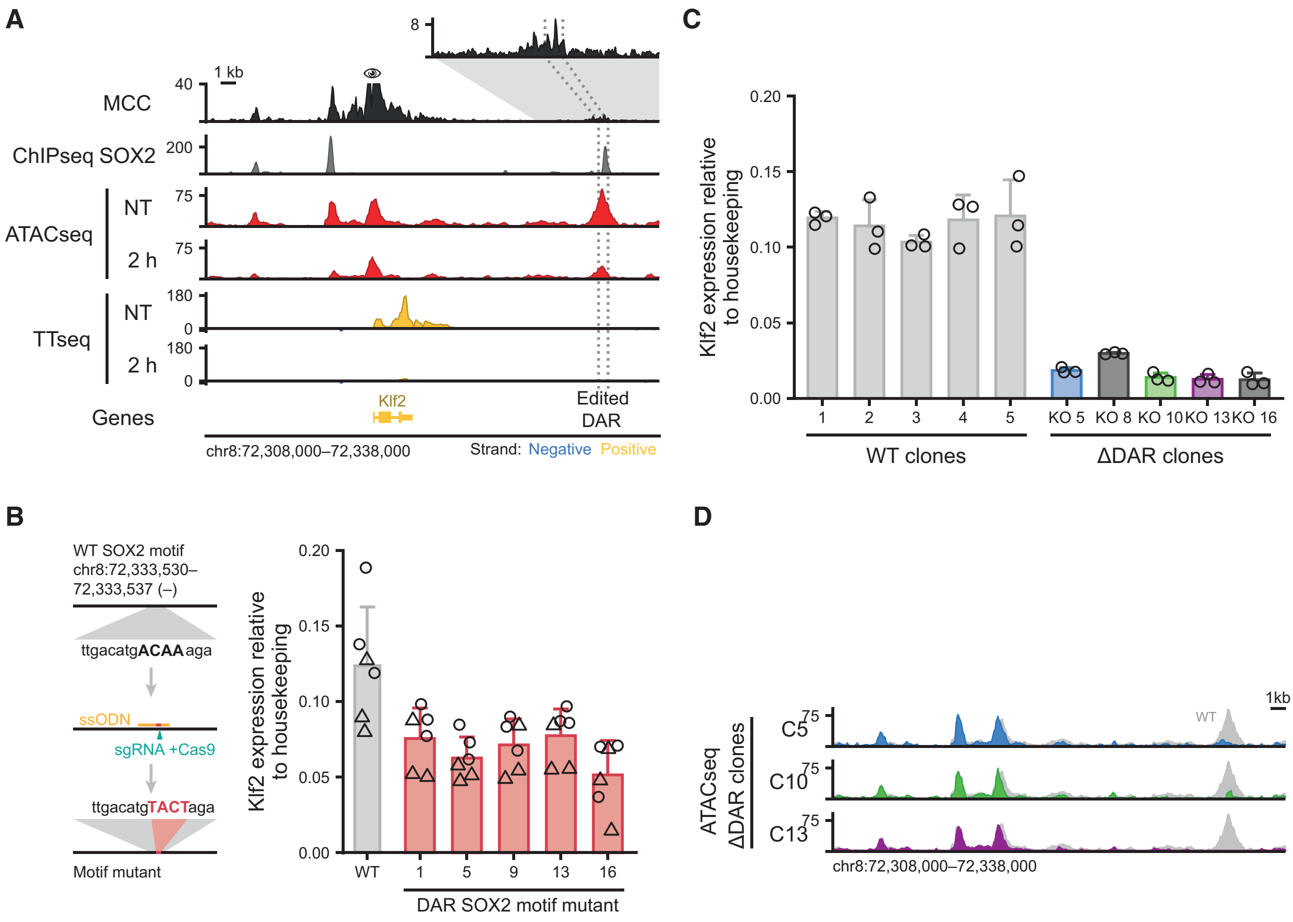

Figure 6. ***Klf2*** **transcription is dependent on a downstream regulatory element that is in physical proximity with the promoter.**

A Genomic tracks showing physical interactions with the *Klf2* promoter measured by micro-capture-C (MCC). SOX2 binding measured by ChIPseq is shown for the untreated (NT) condition. ATACseq and by TT_{chem}seq data is shown for NT and 2 h dTAG-13 treatment in SOX2-FKBP cells. The eye symbol indicates the viewpoint for MCC. Contacts with a downstream DAR is shown in the zoomed-in inset. Dotted lines indicate the region targeted for deletion. *Y*-axes indicate reads per genomic content (RPGC).

B Left: schematic of the procedure to mutate the SOX2 motif. Right: bar plot showing Klf2 expression in wild-type (WT) and mutant clones for the SOX2 motif. Error bars indicate standard deviation for three biological replicates. Two primers set (circle and triangle) are indicated.

C Barplot showing *Klf2* expression in clones with intact (WT) and disrupted DAR (ΔDAR) indicated in (A), as measured by RT–qPCR normalized to the *Rsp26* housekeeping gene. Bar heights indicate means and error bars indicate standard deviation for three biological replicates.

D Genomic tracks showing ATACseq signal around the *Klf2* locus upon knockout of the DAR indicated in (A) in three ΔDAR clones. In gray, accessibility of the NT sample in (A) is shown for comparison. *Y*-axes indicate reads per genomic content.

D) and selected 5 clones in which the putative regulatory element was disrupted. In these clones, and a corresponding set of wild-type clones, we measured *Klf2* expression levels using RT–qPCR (Figs 6C and EV5E). In all the mutant clones the level of *Klf2* was drastically decreased, by ~85% on average, showing that the region indeed serves as a cis-regulatory element that enhances gene expression. These results show that SOX2 is critically important for driving *Klf2* expression through this distal regulatory element. However, the regulatory information is conferred by the SOX2 binding site in combination with the surrounding sequence.

The genomic region surrounding Klf2 contains three SOX2 binding sites that lose accessibility upon SOX2 depletion. Genetic disruption of the 15 kb downstream regulatory element leads to a sizeable (i.e., ~85%) decrease in transcription. We wondered whether the nearby regulatory elements that purportedly also contribute to expression of Klf2 are affected in their accessibility in the disruption clones. To test this, we performed ATACseq in three of the clones (Fig 6D) and could detect hardly any changes in accessibility of the upstream elements. Therefore, we conclude that accessibility of the upstream regulatory regions maintained in an autonomous manner and not influenced by the downstream element. Because ATACseq on the edited clones showed some residual ATAC signal at the DAR, this suggested that the genomic region was not fully deleted. To determine the nature of the disruption of the DAR, we PCR amplified a 2.3 kb region overlapping the DAR and performed paired-end Illumina sequencing (Fig EV5F). This enabled us to assemble the locus following genome editing. For clone 13 we observe a deletion, which is consistent with the lack of sequence reads in the ATACseq. In clone 5 we observed a slightly smaller deletion, but the ATACseq still detects sequence reads in this locus which suggests that the allele is still found in the

 © 2023 The Authors

genome, but was not amplified in our PCR. Interestingly, clone 10 shows a complex pattern of rearrangements following the genome editing. One allele in this clone still harbors an intact SOX2 binding motif, while the upstream sequence is rearranged. These results combined with the motif mutation results suggest that, not only the presence of SOX2 binding motifs, but the integrity of the entire locus is essential for proper enhancer activity. Our genome editing results show that using inducible depletion of a pioneer factor coupled to nascent transcription and chromatin accessibility mapping enables us to determine functional cis-regulatory elements that are necessary for transcription.

Discussion

Here we use acute protein depletion to determine the dynamics of chromatin remodeling induced by pioneer transcription factors. We find that loss of SOX2 leads to a rapid loss of accessible sites in the genome. We find that already within half an hour of depletion over a thousand open chromatin sites are significantly decreased in accessibility. This is consistent with a previous study that showed rapid loss of accessibility following OCT4 depletion (Friman *et al*, 2019). Our observations are also in line with recent reports that used catalytic inhibitors of the SWI/SNF complex or a molecular degrader of SWI/SNF complex member BRG1 that showed that loss chromatin remodeling activity results in a rapid change in the open chromatin landscape (Iurlaro *et al*, 2021; Schick *et al*, 2021; Xiao *et al*, 2022). This indicates that continuous activity of both the transcription factors and these complexes is required for the maintenance of open chromatin. Our results show that maintenance of open chromatin is a highly dynamic process. The fast and direct effects on chromatin and gene expression emphasize the importance of studying the early molecular events that are in play that can lead to transition to a different cell state.

Open chromatin sites are highly predictive for gene expression dynamics

One of the challenges of assigning functionality to the regulatory landscape of cells is that the number of putative regulatory sites is often much larger than the number of genes. How these regulatory elements collaborate to drive gene expression is an important unresolved question. We have developed a statistical framework to predict changes in transcription based on changes in chromatin accessibility. We show that integrating acute protein depletion methods with direct read-outs of chromatin features (e.g., ATACseq) and transcription (with TT_{chem}seq) is a powerful combination to identify functional accessible chromatin sites. The highly dynamic nature of the open chromatin sites means that non-acute methods will be much less accurate in predicting the transcriptional changes, because indirect effects start to play an important role once secondary regulators are up- or downregulated. An example of this is the *Klf2* gene, which encodes for a transcription factor and rapidly loses expression following SOX2 depletion and may in turn regulate other genes.

We find that the highest predictive power is attained when we use a function where weights are assigned in a decreasing manner from the TSS of a gene. This seems to mimic the decreasing contact frequency that is observed as a consequence of polymer dynamics of the chromatin fiber (Zuin *et al*, 2022). Although the optimal predictive power is reached when we use a distance of 20 kb, we would like to note that our analyses do not rule out regulation over larger distances. However, our results strongly suggest that the majority of gene regulation occurs relatively close to the promoter of a gene. Activation of genes by enhancers over larger distances may depend on the presence of additional regulators such as CTCF and cohesin (Kubo *et al*, 2021; preprint: Rinzema *et al*, 2021). A complicating factor in predicting regulation over larger distances is that although it may result in the correct prediction of genes that are dependent on a particular long-distance enhancer, it will also result in an even stronger increase in false positive predictions. How the cell can make the correct decision to upregulate the correct gene is an important question for the future. One possibility is for instance promoter enhancer compatibility (Martinez-Ara *et al*, 2022), which is something that we cannot easily explore in our system.

Our results clearly show that SOX2 pioneer activity is a stronger predictor for changes in transcription than SOX2 binding on its own. In fact, SOX2 binding sites where we detect no changes in accessibility effectively have a much weaker predictive power for changes in transcription. This has important implications for how to interpret genome-wide binding data, particularly for pioneer factors. How pioneer factor activity and the formation of accessible sites contribute to gene expression is not fully understood. It may create binding sites for secondary transcription factors (Isbel *et al*, 2022) which, in turn, recruit co-factors such as Mediator (Allen & Taatjes, 2015). The compendium of genomic regions that are directly controlled by pioneer factors that we describe here can serve as a starting point for further investigating these questions. The degron lines that we have developed are a powerful tool for these future research questions. What the function of SOX2 binding at non-pioneer sites is—if any—is an interesting question for the future, although our results suggest that SOX2 binding alone is involved in gene expression regulation to a much lesser degree.

What distinguishes genomic regions where SOX2 exerts pioneer activity from regions where SOX2 is merely binding is unknown? Although binding levels of SOX2 seem to be higher on average, there is a stronger association with mSWI/SNF subunit SS18 and there seems to be enrichment of the histone modification H2BK20ac no definitive combination of factors could be defined that discerns the pioneer regions from non-pioneer regions. Most likely, the difference is also encoded in the DNA. However, current basic models using TF binding motifs cannot yet help in distinguishing these sites. Likely, the defining features are higher-order interactions that are not captured by the current model. Deep learning methods such as convolution neural networks (Avsec *et al*, 2021) should be able to capture these interactions. We expect that datasets like the one presented here can aid in the delineation of functional regulatory elements based on genomic sequence, which can in turn help to predict the effect on mutations in regulatory elements.

Following the genetic disruption of the upstream *Klf2* regulatory element only 15% of Klf2 expression remains. We identified two elements flanking the *Klf2* gene that both lose accessibility following SOX2 depletion. We can consider two models for the regulation by these elements, an additive and a super-additive model. In the additive model gene expression is the sum of activity of the flanking regulatory elements. This would mean that the distal downstream

© 2023 The Authors

element drives 85% of expression and the proximal element a much smaller part of expression. Massively parallel reporter assays show that the both elements drive expression in roughly equal measure in an episomal context (Martinez-Ara *et al*, 2022). An alternative is a super-additive model in which multiple weak enhancers synergistically activate gene expression to a higher level than would be predicted from the sum of all enhancer activities. This behavior has been observed for in the *Fgf5* locus (Thomas *et al*, 2021). Further genetic dissection of the *Klf2* locus is required to determine the mode of action in this locus.

A model for SOX2 dependent regulatory element establishment

We have shown that not all SOX2 binding sites that are accessible lose accessibility and our computational analyses give insight into which additional factors assist SOX2 in maintaining the accessible state. Apart from SOX2, SS18 is the most predictive protein for differential accessibility following SOX2 depletion. Our separation of function assays showed that SOX2 needs both its DNA binding domains and the transactivation domain for the formation of open chromatin. Based on our data we propose the following model: SOX2 binds to its cognate binding site, which can be the compound site with OCT4 or the sites where it binds alone or with a different factor. CryoEM models have shown that binding of OCT4 and SOX2 partially disrupt the DNA interface with the histone octamer (Dodonova *et al*, 2020; Michael *et al*, 2020). However, our reconstitution experiments have shown that the DNA binding domain alone, which was used in the CryoEM experiments, is not enough to maintain accessible chromatin. Rather, both the DNA binding domains and the transactivation domains are necessary for the maintenance of open chromatin. We therefore propose that following binding, it recruits the SWI/SNF complex through its transactivation domain, resulting in the remodeling of the nucleosomes around the binding site. Furthermore, the genomic regions that are kept in an accessible state by SOX2 are enriched for acetylated histone modifications such as H2BK20ac, H3K64ac, and H3K27ac. The latter two are known to be a consequence of the activity of the p300 acetyltransferase (Di Cerbo *et al*, 2014; Dancy & Cole, 2015; Pradeepa *et al*, 2016), but whether p300 is directly recruited by SOX2, a secondary consequence of SOX2 binding or recruited through a parallel mechanism remains to be determined. Recent results have also shown that acetylated histone tails promote cooperativity between SOX2 and OCT4 (Sinha *et al*, 2023). Furthermore, acetylation of histones is thought to change the electrostatic interaction of DNA with the histone octamer which could assist the nucleosome remodelers in creating accessible sites as a direct consequence of SOX2 binding. An elegant recent study examined the remodeling of nucleosomes by Nanog, Pouf5f3 and Sox19b during zygotic genomic activation in zebrafish. These orthologs of NANOG, OCT4 and SOX2 were able to remodel nucleosomes more efficiently when nucleosome occupancy was high, suggesting that the proper positioning of nucleosomes can be a contributor to pioneer activity (Miao *et al*, 2022). Specific nucleosome positions may arrange the binding site of the pioneer factor such that it can be bound more efficiently (Michael & Thomä, 2021).

Our results emphasize the importance of using acute depletion tools to dissect direct and indirect relationships in the gene regulatory network. Furthermore, they enable the distinguishing of functional and non-functional binding sites of a specific transcription factor. These two features combined should ultimately lead to improved models for how transcription factors induce gene expression changes as a consequence of developmental cues or external stimuli.

Materials and Methods

Cell lines

Mouse Embryonic Stem Cells E14Tg2A (129/Ola) cell lines were cultured on 0.1% gelatin-coated plates in serum-free DMEM/F12 (Gibco) and Neurobasal (Gibco) medium (1:1) supplemented with N-2 (Gibco), B-27 (Gibco), BSA (0.05%, Gibco), 10 × 4 U of Leukemia Inhibitory Factor/LIF (Millipore), MEK inhibitor PD0325901 (1 μM, Selleckchem), GSK3-β inhibitor CHIR99021 (3 μM, Cayman Chemical) and 1-Thioglycerol (1.5×10^{-4} M, SigmaAldrich). The cell lines were passaged every 2 days in daily culture. The OCT4-FKBP (v6.5 mESC) were kindly provided by Richard Young (Boija *et al*, 2018). E14 mESC were used for NANOG and SOX2 (IB10 mESC) targeting. For depletion treatment, the OCT4-, SOX2-, NANOG-FKBP (Liu *et al*, 2020) cell lines were treated with a final concentration of 500 nM dTAG-13 or DMSO (NT, not treated). For time course experiments, cells were seeded overnight and treated the next day, by inducing protein depletion at different time points and harvesting at the end of the time course. Prior to treatment medium was refreshed. DMSO and dTAG was added in parallel. For wash-off experiments, cells were passaged and maintained in medium without dTAG-13.

Gene targeting

For the knock-in of the *FKBP* sequence at the genes of interest, we used previously described plasmids and approach (SOX2 fkbp-donor, Addgene # 175552; SOX2 sgRNA, Addgene #175553; NANOG donor, Addgene # 175554; NANOG sgRNA, Addgene # 175555) Briefly, cells were transfected with the plasmids containing the gRNA sequence and the donor plasmid designed to include the FKBP-2xHA-P2A-[GFP/mCherry] in between two homology arms for the gene of interest. After transfection, cells were sorted in 96-well plates and manually picked for genotyping using PCR and western blot. Homozygous clones responding to dTAG-13 were used for experiments. Primers used are listed in Dataset EV2.

Western blotting

Cells were harvested and lysed in RIPA lysis buffer (150 mM NaCl, 1% NP-40, 0.5% sodium deoxycholate, 0.1% SDS, and 25 mM Tris (pH = 7.4)). A 10% SDS–PAGE gels were used to separate proteins. Protein was transferred to a pre-activated PVDF membrane using Trans-Blot Turbo Transfer System (Bio-Rad). The blots were incubated with the following primary antibodies overnight at 4°C: SOX2 (1:1,000, D9B8N, Cell Signaling), GFP (1:1,000, ab6673, abcam) and as loading control HSP90 (1:2,000, 13171-1-AP). After incubation, the blots were washed three times with TBS-0.1% Tween-20. The blots were then incubated with secondary antibody against rabbit IgG at room temperature for 1 h, following by 3-time

© 2023 The Authors

TBS-0.1%-Tween-20. The proteins attached with antibodies were hybridized with Clarity Western ECL Substrate reagent (Bio-Rad) and visualized in a ChemiDoc MP Imaging System (Bio-Rad).

ATACseq

Chromatin accessibility was assessed using the previously established protocol for ATACseq (Buenrostro *et al*, 2013). Briefly, cells were harvested and washed 1× in cold PBS. 50,000 cells were counted and nuclei were lysed using 2× lysis buffer (1 M Tris–HCl pH 7.5, 5 M NaCl, 1 M MgCl2, 10% IGEPAL). Cells were spun down incubated for 1 h at 37°C in tagmentation buffer (20 mM Tris (hydroxymethyl)aminomethane; 10 mM MgCl2, 20% dimethylformamide, brought at pH 7.6 with acetic acid) containing custom Tn5 produced by the NKI in-house Protein Facility. For amplification of the tagmented DNA, two rounds of PCR were used using KAPA kit amplification protocol and sequencing adapters were included during the PCRs for library preparation. Fragments smaller than 700 bp were purified using SPRI beads selection. Quality of the libraries was checked on a Bioanalyzer before sequencing. All ATACseq experiments for NANOG and OCT4 depletion were done in duplicate and for SOX2 we performed experiments in duplicate as well as in an independent biological clone.

Genome editing of Klf2 regulatory element

For deletion of the *Klf2* downstream regulatory region, SOX2-FKBP cells were used. CRISPR-Cas9 was used to induce the deletion using sgRNAs targeting sites flanking the region. sgRNAs were cloned into a plasmid expressing GFP (Plasmid #111596, Addgene). The unmodified PX330 plasmid (Plasmid #42230, Addgene) was delivered into the cells to express the Cas9 protein. Plasmids containing the sgRNA and Cas9 were co-transfected into the cells with nucleofection Kit Amaxa (VPH-1001) following the manufacturer's protocol using the A030 program. After 2 days, single GFP positive cells were sorted in a 96 well plate precoated with 0.1% gelatin and expanded. Clones were manually picked and expanded for further experiments. Clones were genoptyped for the homozygous disruption using PCR and Sanger was used to validate the disruption with primers spanning the sites of editing.

For mutation of the SOX2 Motif sequence SOX2-FKBP cells were used. CRISP-Cas9 and a sgRNA targeting the locus was cloned into a CAS9-GFP expression plasmid (Plasmid #111596, Addgene). Modification of the locus was achieved co-transfecting a repair template designed to contain the desired mutation and 80 bp homology arms. The repair template was ordered as Altr-R ssODN template and 100 μM were co-transfected with 1 μg sgRNA-Cas9-Egfp plasmid with nucleofection (Kit Amaxa (VPH-1001), A030 program). After transfection the DNA-PK inhibitor M3814 was added to the medium (1 nM). Transfected cells were sorted for GFP positive cells after 2 days in bulk and further expanded. After 1 week, live cells were re-sorted in single cells and clones were isolated. Clones homozygous for the editing were further expanded and used for analysis.

Klf2 gene expression RT–qPCR

Clones for the homozygous modifications were expanded. Clones from the parental cell were obtained to control for the endogenous variation of *Klf2* gene expression. RNA, DNase I treated, was isolated in triplicates from cells using RNeasy Kit (Qiagen cat #74106). Purified RNA was retro-transcribed into cDNA with the iScript cDNA synthesis kit (Biorad). Expression of the *Klf2* gene was quantified with RT–qPCR, with primers for *Rps26* and *Klf2* genes using SensiFast No-Rox kit (Bioline). The expression of Klf2 gene was calculated using the $2^{-\Delta\Delta Ct}$, normalizing over the housekeeping *Rsp26* expression. Primers used are listed in Dataset EV2.

Amplicon sequencing

To characterize the deletion at the downstream *Klf2* DAR we performed amplicon sequencing. The genomic region corresponding to the region of interest was amplified using PCR using primers amplifying a 2 kb region, spanning the putative deletion site. The PCR product was purified using Qiagen PCR purification Kit (cat n. 28106) and the product was tagmented using 0.02 mg/ml Tn5 for 10 min at 55°C. The reaction was stopped by adding 0.02% SDS for 5 min at 55°C. Similar to the ATACseq protocol, tagmented DNA was used as input for library preparation and amplified by KAPA HiFi DNA polymerase, following the manufacturer's protocol. Libraries size was checked on Bioanalyzer and samples were sequenced on a MiSeq Nano, paired end 250 bp reads length.

Ectopic expression of SOX2 domain mutants for ATACseq

SOX2 protein sequence (uniprot #P48432) was used as a reference for identifying the HMG (DNA binding domain), TAD (transactivation domain) and full length of SOX2 protein, and the corresponding mRNA sequence was used to create the truncation of the proteins. The SOX2 endogenous start codon was added to promote expression of the domains. An eGFP cassette was included in the design to be in-frame with the different domains and used for selection of domains expression. The designed constructs were ordered from Twist Bioscience and cloned into an expression vector with the EIF1A promoter. The eGFP only vector was used as control. Plasmids (5 μg/10^6 cells) were transfected into SOX2-FKBP degron cells with nucleofection following the AMAXA protocol and A030 program. For each plasmid, 5 million cells were transfected and then seeded dividing the total volume of cells into two plates pre-coated with 0.1% gelatin. At 46 h after nucleofection cells were treated with DMSO or 500 nM dTAG-13 to deplete the endogenous SOX2-FKBP. At 48 h cells were harvested and GFP positive cells were sorted and ATACseq was performed directly after sorting.

ChIP-seq

Chromatin immunoprecipitation (ChIP) was performed using available protocol (King & Klose, 2017) with some modifications. For ChIP-seq experiments, cells were expanded and harvested at the end of the time-point for chromatin isolation. Chromatin was fixed using double cross-linked protocol. DSG (disuccinimidyl glutarate) (2 mM final concentration) was added to cell suspension for 45 min at RT. Formaldehyde was then added (to a concentration of 1%) for an additional 15 min. Reaction was quenched with Glycine (2 M). 5 million cells were harvested for each IP. Cells were lysed lysis buffer 1 (50 mM Hepes, 140 mM NaCl, 1 mM EDTA, 10% glycerol, 0.5% N40, 0.25% Tx100), followed by buffer 2 (10 mM Tris, 200 mM

© 2023 The Authors

NaCl, 1 mM EDTA, 0.5 mM EGTA) and then kept in buffer 3 (10 mM Tris, 100 mM NaCl, 1 mM EDTA, 0.5 mM EGTA, 0.1% Na-DOC, 0.5% lauroylsarcosine). Chromatin was sheared on Bioruptor Pico (Diagenode) for 5–7 cycles. Shearing of chromatin was controlled on agarose gel and shearing was stopped when reaching an average size of chromatin from 200 to 1,000 base pairs. Protein beads (DyanaBeads) were prepared and coupled for 6 h to the antibody of interest. Before adding chromatin beads/antibodies were washed in PBS-BSA 0.5% and then washed three times. Chromatin was added to beads-antibodies overnight. Samples were washed in RIPA buffer cold RIPA (50 mM Hepes, 500 mM LiCl, 1 mM EDTA, 1% NP40, 0.7% Na-DOC) 10 times. Followed by one extra wash in TBS buffer (20 mM Tris, 150 mM NaCl) and chromatin was eluted (50 mM Tris, 10 mM EDTA, 1% SDS). DNA was decrosslinked overnight at 65C and treated with RNaseA for 1 h at 37°C. Followed by Proteinase K treatment (0.1 mg/ml) AT 55°C for 1 h. DNA was finally eluted and purified using miniElute PCR purification kit (Qiagen). DNA fragments were used for library preparation following KAPA HTP Library preparation Kit (Roche).

Antibodies used in this study: HA (ab9110 10 μg per ChIP), anti SOX2 (SOX2; AF2018, R&D Systems; 5 μg per ChIP). OCT4 (OCT4; AF1759, R&D Systems; 5 μg per ChIP). 10% of chromatin was used as input.

TT_{chem}seq

Libraries for TT_{chem}seq were prepared following a published protocol (Gregersen *et al*, 2020). SOX2-FKBP cells were seeded and the day after treated with 500 nM of dTAG-13 (0.5, 1, 2, 6 hpd) or DMSO as control. Cells were labeled with 2 mM 4SU for 10 min. Total RNA was isolated and fragmented. The 4SU-biotin labeled RNA was enriched using streptavidin coated MicroBeads. Libraries were prepared using KAPA RNA HyperPrep kits (Roche) using dual indexing adapters. Libraries were sequenced on a NextSeq 550.

Quantitative mass spectrometry

For quantitative measure of protein abundance after dTAG treatment, cells from the SOX2-FKBP and parental cells were expanded and treated with either DMSO or 500 nM dTAG-13, for 30 min. Cells were harvested and 30 million cells per condition were centrifuged at 500 $g \times 5$ min, washed with PBS and pellet was snap frozen. All samples were prepared in quadruplicate before analysis.

For protein digestion, frozen cell pellets were lysed in 5% SDS lysisbuffer, boiled and sonicated. Aliquots corresponding to 100 μg of protein were digested using S-Trap micro-columns (ProtiFi) according to the manufacturer's protocol. In short, samples were reduced and alkylated using DTT (20 mM, 15 min, 55°C) and IAA (40 mM, 10 min). The samples were acidified and a methanol TEAB buffer was added, prior to loading on the S-Trap column. Trapped proteins were washed four times with the methanol TEAB buffer and then digested for 2 h at 47°C using Trypsin (Sigma). Digested peptides were eluted and dried in a vacuum centrifuge before LC–MS analysis.

Prior to mass spectrometry analysis, the peptides were reconstituted in 2% formic acid. Peptide mixtures were analyzed by nanoLC-MS/MS on an Orbitrap Exploris 480 Mass Spectrometer equipped with an EASY-NLC 1200 system (Thermo Scientific). Samples were directly loaded onto the analytical column (ReproSil-Pur 120 C18-AQ, 2.4 μm, 75 μm × 500 mm, packed in-house). Solvent A was 0.1% formic acid/water and solvent B was 0.1% formic acid/80% acetonitrile. Samples were eluted from the analytical column at a constant flow of 250 nl/min in a 140-min gradient, containing a 124-min linear increase from 6 to 24% solvent B, followed by a 16-min wash at 90% solvent B.

Mass spectrometry data analysis

Raw data were analyzed by MaxQuant (version 2.0.1.0) using standard settings for label-free quantitation (LFQ). MS/MS data were searched against the Swissprot Mus Musculus database (17,073 entries, release 2021_04) complemented with a list of common contaminants and concatenated with the reversed version of all sequences. The maximum allowed mass tolerance was 4.5 ppm in the main search and 0.5 Da for fragment ion masses. False discovery rates for peptide and protein identification were set to 1%. Trypsin/P was chosen as cleavage specificity allowing two missed cleavages. Carbamidomethylation was set as a fixed modification, while oxidation and acetyl (protein N-term) were used as variable modifications. LFQ intensities were $\log_2$-transformed in Perseus (version 1.6.15.0), after which proteins were filtered for 3 out of 3 valid values in at least one sample group. Missing values were replaced by imputation based on a normal distribution (width: 0.3 and downshift: 1.8). Differentially expressed proteins were determined using a Student's *t*-test (threshold: FDR = 0.05 and S0 = 0.1).

The mass spectrometry proteomics data have been deposited to the ProteomeXchange Consortium via the PRIDE [1] partner repository with the dataset identifier PXD043672.

Micro-capture-C

Micro-capture-C was performed as previously described (Hua *et al*, 2021). Cells were fixed with formaldehyde (2% (wt/vol)) for 10 min at room temperature and permeabilized with digitonin (0.005% (wt/vol)). 2–3 × 10^6 cells were treated with MNase (NEB M0247) ranging from 10 to 30 Kunitz U in 800 μl of custom buffer (Tris–HCl pH 7.5 10 mM, $CaCl_2$ 1 mM) for 1 h at 37°C. Ethylene glycol-bis(2-aminoethylether)-N,N,N′,N′-tetraacetic acid (EGTA) 5 mM (Sigma E3889) was added to quench the reaction. The reaction was centrifuged (5 min, 300 × *g*) and the supernatant discarded. The cells were resuspended in 1 ml phosphate buffered saline with 5 mM EGTA of which 200 μl was used to measure the digestion efficiency. The remainder was centrifuged (5 min, 300 × *g*) and the supernatant discarded. Cells were resuspended in DNA ligase buffer (Thermo Scientific EL0013) supplemented with dNTPs (dATP, dCTP, dGTP and dTTP; 400 μM final concentration of each (Thermo Fischer R0191)); EGTA 5 mM; T4 Polynucleotide kinase PNK 200 U/ml (NEB M0201L); DNA Polymerase I Large (Klenow) Fragment 100 U/ml (NEB M0210L) and T4 DNA ligase 300 U/ml (Thermo Scientific EL0013). The reaction was incubated at 37°C for 2 h followed by 20°C for 8 h using an Eppendorf Thermomixer at 500 rpm. The ligation reaction was centrifuged and the supernatant was discarded. The chromatin was decrosslinked with proteinase K at 65°C (> 2 h) and the DNA was extracted. Double oligonucleotide capture was performed as previously described

 © 2023 The Authors

(Davies *et al*, 2015). Data were analyzed with a custom analysis pipeline specifically developed for MCC data analysis.

Data preparation

Sequencing data from ATACseq, ChIPseq, and TT_{chem}seq were mapped to the mm10 reference genome with the bwa program version 0.7.17-r1188 using the command "bwa mem -M" (Li & Durbin, 2009). Alignments were filtered with samtools version 1.10 using "samtools view -h -b -q 10" (Li *et al*, 2009). Alignments were deduplicated using the "MarkDuplicates" command from Picard tools version 2.12.0, using the argument "REMOVE_DUPLICATES = true". For TT_{chem}seq data, alignments were split up by strand as described in (Gregersen *et al*, 2020). For ChIPseq data, peaks were called using MACS2 version 2.2.7.1 with the argument "-g mm -f BEDPE –keep-dup-all" using an input sample as the "control" argument (Liu, 2014). We conducted the rest of the analyses in R version 4.0.5 and Bioconductor version 3.12 (Huber *et al*, 2015). Bigwig coverage tracks were created using the GenomicRanges, rtacklayer and GenomicAlignments R/Bioconductor packages (Lawrence *et al*, 2009, 2013), scaling coverage to 1× genome size (reads per genomic content).

Identification of DARs

To identify DARs, peak calling was performed on merged data from timeseries experiments for the SOX2-FKBP line and all three degron lines separately, using MACS2 version 2.2.7.1 with the arguments "-g mm –nomodel –keep-dup all". Subsequently, the number of overlapping paired-end fragments was counted per peak. Peaks were subsequently filtered by requiring every peak to have more than 10 reads in more than 2 samples for the SOX2-FKBP experiment and, separately, more than 10 reads in more than 6 samples for all three degron lines. Differential accessibility analysis was performed by using DESeq2 with the design argument set to "∼ time" for the SOX2-FKBP line and "∼ genotype + time + time:genotype" for all three degron lines. The "nbinomWaltTest()" function was used with default arguments to estimate peak-wise *P*-values and effect sizes assuming the null hypothesis that there was no change over time. The results were generated per timepoint using the "results" function with the contrast specifying a timepoint against non-treated with the argument "alpha = 0.05". A peak was considered differentially accessible when the false discovery rate corrected *P*-value was below 0.05.

For finding a set of nDARs that are stable for Figs 5A and EV4A, we used the "results" function with the arguments "altHypothesis = 'lessAbs', lfcThreshold = 1" and considered a peak stable when the false discovery rate corrected *P*-value was below 0.05. We then used the top n peaks with the smallest $\log_2$ fold change as the stable set, where n is the number of DARs under consideration.

For finding a set of comparable nDARs for the SOX2-FKBP line, we calculated the $\log_{10} + 1$ transformed maximum coverage at 500 bp regions centered on the summit of ATACseq peaks of both non-treated ATACseq and non-treated SOX2 ChIPseq tracks. We used the "matchRanges" function in the nullranges package (Davis *et al*, 2023) with the ATACseq and ChIPseq heights as the covariates, in addition to using the "method = 'stratified'" argument.

Analysis of DNA binding motifs

Motif position weight matrices (PWMs) were taken from the JASPAR 2020 core vertebrate dataset as provided in the JASPAR2020 R/Bioconductor package (Castro-Mondragon *et al*, 2022). After matching PWMs to corresponding genes and filtering the PWMs on expression of the DNA binding factor, 408 PWMs were retained for further analysis. The presence or absence of a PWM match in a peak was determined using the motifmatchr R/Bioconductor package (Schep, 2020). Motif enrichment was determined by using logistic regression wherein peak set membership, along with peak specific GC fraction and total number motifs found in a peak, was used to model the absence or presence of a motif in a peak. The coefficients for peak set membership were used as the effect size (log odds ratio) and significance was tested using the Wald test in this model.

Quantification of accessibility dynamics

A selection of peaks was made by using the DESeq2's likelihood ratio test "nbinomlrt()" using "∼ 1" as the reduced model and "∼ time" as the full model. Peaks that had an FDR-corrected *P*-value below 0.05 were kept for further analysis. Library size normalized counts per peak were rescaled to a minimum of 0 and maximum of 1. The "drc" R package (Ritz *et al*, 2015) was used to fit log-logistic curves to the data, which uses the following formula:

$$f(t) = A + \frac{K - A}{\left(1 + e^{B(\log(t) - \log(T_{50}))}\right)^{f}}$$

wherein t is the sampled time, A and K are the lower and upper asymptotes respectively, B is the slope at the inflection point T_{50}, and f is also known as the Hill exponent. Multiple parameterizations of models were fit, wherein B and T_{50} were estimated every time, whereas in some models A, K and f were fixed at 0, 1 and 1 respectively. To balance model complexity and goodness of fit between different models, the difference in Akaike's information criterion (ΔAIC) was used to select the simpler model when the ΔAIC was less than 2. Moreover, we used the "lm()" function to fit the data with three polynomial models of 0th up to 2nd degree. Log-logistic models were preferred over 2nd degree polynomial when the $\Delta AIC < 2$, whereas 0th and 1st degree polynomials were preferred over log-logistic models when $\Delta AIC < 2$. Of the 15,906 DARs that were selected initially, the T_{50} of 9,692 DARs (for which the log-logistic models were preferred) are used in Fig 3.

Analysis of TT_{chem}seq data

To determine transcribed units (TUs), all TT_{chem}seq data was pooled per strand, the genome was tiled into 200 bp bins and middle-fragment positions were counted. The tiles were then discretized into low and high transcriptional states with a 2-state hidden Markov model using Poisson-Lognormal distributions with the STAN R/Bioconductor package (Zacher *et al*, 2020). Runs of consecutive bins of the same state (ignoring single-bin interruptions) were then merged to form putative TUs. Putative TUs were then filtered to have more than 25 overlapping fragments. These TUs were then refined to basepair position by finding positions in a 400 bp window

where the positive difference between two consecutive coverage positions exceeded a threshold of 1. The putative TUs were then associated to genes in the RefSeq annotation (O'Leary *et al*, 2016). TUs associated with multiple small genes (such as miRNAs, Jaccard index between TU and annotated gene < 0.02) were annotated as such. TUs associated with multiple, larger genes were split when an annotated transcription start site (TSS) internal to the TU was observed to be followed by an increased wave of transcripts. We required these TSSs to be followed by a median increase in coverage of 5 fragments within a 2 kb window centered at the annotated TSS, relative to 5′ of the TSS. Subsequently, fragments were counted in the resulting TUs. TUs were further filtered to have more than 10 fragments in at least two samples, after which they were subjected to differential expression analysis with the DESeq2 R/Bioconductor package (Love *et al*, 2014). The "nbinomWaldTest()" function with default arguments was used to test the contrasts of treatment time against untreated.

Prediction of downregulated DEGs

First, various association rules were used to associate peaks with genes. The TSS was taken empirically from the TT_{chem}seq data rather than from other annotations. The "distance threshold" rule took every peak with some distance to the TSS of a gene. The "basal + extension" rule associated peaks within a basal domain and in an extended domain (until another gene's basal domain was encountered) (McLean *et al*, 2010). The "KNN, many TSSs one peak" rule searched for the k nearest genes for every peak, and the "KNN, one TSS many peaks" searched for the k nearest peaks for every gene. For every gene in consideration, the associated peaks were counted by category (down DAR, nDAR or up DAR). These counts were then used in a logistic lasso regression model to predict a yes/no outcome for gene expression status (DEG or nDEG), using the glmnet R package version 4.1-3 (Friedman *et al*, 2010). Stable control nDEGs were chosen such that their average transcription are similar to the DEGs under consideration and moreover fail to reject the null hypothesis of a Kolmogorov-Smirnoff test that the distributions are equal. Because such nDEGs were more numerous than DEGs, these could be resampled to estimate variability in predictions. Appropriate parameters for the association rules were determined by performing a parameter sweep, and choosing a distance, k, extended and basal domains, that minimized cross-validation error. Because a simple distance threshold outperformed other association rules, we explored other distance-based weighting schemes.

Second, for the kernel-based predictions, a 10 Mb window around the TSS was used to putatively associate peaks to a gene. Every gene-peak association was then weighted by the distance between the TSS and the summit of a peak according to the kernel functions, which heavily penalize far-distance associations. These were then summarized per gene by taking the sum of weights of all putatively associated peaks for every category of peak. These weighted sums per peak categories were then used as predictors in a similar logistic regression model as described above. Again, appropriate parameters for the kernel functions were found by performing a parameter sweep over various distances and choosing these such that they have minimal cross-validation error. Further predictions were performed for the downregulated DEGs at 2 h, using the (truncated at 5 Mb) Cauchy density function as the kernel function with the "scale = 20e3" argument in R's "dcauchy()" function, and the down DARs, nDARs and up DARs at the 2 h dTAG treatment contrast were used as the predictor categories. Predictions in Figs 5C and EV4D also used SOX2 binding sites as determined by ChIPseq as predictors.

Random forest classification with Cistrome data

Data for mouse factors and histones were downloaded as batches from the Cistrome Data Browser website (http://cistrome.org/db/) and listed in Dataset EV1. Datasets were enriched from datasets from embryonic stem cells by filtering for the following inclusion and exclusion criteria. Inclusion criteria (i) the cell line was described as "V6.5", "E14" or "Mouse embryonic stem cells", (ii) the cell type was described as "Embryonic Stem Cell" or "Stem Cell" and (iii) the tissue was described as "Embryo". Exclusion criteria were (i) cell types described as "Embryonic Cortex", "B lymphocyte", "Embryonic Fibrobtst", "Fibroblast", "Lymphocyte", "Junction cell", "Neuron", "Epithelium", "Hemangioblast", "Mesodermal Progenitor Cell", "Progenitor Cells, Mesenchymal", "Trophoblast Giant Cell" or "Trophoblast Stem Cell" and (ii) tissues described as "Brain", "Colon", "Ectoderm", "Endoderm", "Intestine" or "Liver". Moreover, datasets were filtered out that had less than 1,000 peaks in standard chromosomes, or that had no or multiple associated factors or histones (notably, FAIREseq data and a single H3K9ac, H3K14ac combined ChIPseq dataset). The filtered datasets we considered are listed in the "public_datasets_ev_used.xlsx" file. To filter for redundant factors, the Jaccard index was calculated between the dataset and the ATACseq peakset (regardless of DAR status). Per factor, datasets with the highest Jaccard index were kept for further analysis. After this filtering, we separately considered 233 factor datasets and 38 histone datasets as predictors for random forest classifications using the randomForestSRC R package (Ishwaran & Kogalur, 2022). The independent variable to predict was the downregulated DAR status of peaks, among matched control nDAR peaks. Variable importance with confidence intervals were calculated by subsampling inference, and reported variable importance is the normal-Z subsampling confidence intervals with delete-d jack-knife estimator.

Quantification and statistical analysis

Quantification of sequencing data was performed by mapping sequencing reads to the reference genome and counting fragments in features of interest. Statistical analysis was performed with R software version 4.0.5 in combination with Bioconductor version 3.12 R packages. The details are described in the methods and in the figure legends.

Data availability

Fastq files for all libraries have been deposited at NCBI database under GSE209529 (http://www.ncbi.nlm.nih.gov/geo/query/acc.cgi?acc=GSE209529).

Expanded View for this article is available online.

 © 2023 The Authors

Acknowledgements

We would like to thank Miguel Martinez-Ara for help with the genomic editing of the *Klf2* enhancer. We thank Richard Young for providing the OCT4-FKBP cell line. We thank the NKI Genomics Core facility, FACS facility, Protein facility, Proteomics facility and Research High Performance Computing facility. We thank members of the de Wit lab for critically reading the manuscript. Work in the de Wit lab is supported by the Dutch Research Council (016.161.316, Vidi) and the European Research Council (865459, "FuncDis3D"). This research was supported by Oncode Institute which is partly financed by the Dutch Cancer Society. Research at the Netherlands Cancer Institute is supported by institutional grants of the Dutch Cancer Society and of the Dutch Ministry of Health, Welfare and Sport.

Author contributions

Elzo de Wit: Conceptualization; supervision; funding acquisition.
Michela Maresca: Conceptualization; data curation; formal analysis; writing – original draft; writing – review and editing. **Teun van den Brand:** Conceptualization; data curation; formal analysis; writing – original draft; writing – review and editing. **Hangpeng Li:** Methodology. **Hans Teunissen:** Methodology. **James Davies:** Methodology.

Disclosure and competing interests statement

JD is a co-founder of Nucleome Therapeutics and provides consultancy to the company. The remaining authors declare no competing interests.

References

Allen BL, Taatjes DJ (2015) The Mediator complex: a central integrator of transcription. *Nat Rev Mol Cell Biol* 16: 155–166

Ambrosetti DC, Basilico C, Dailey L (1997) Synergistic activation of the fibroblast growth factor 4 enhancer by Sox2 and Oct-3 depends on protein-protein interactions facilitated by a specific spatial arrangement of factor binding sites. *Mol Cell Biol* 17: 6321–6329

Avsec Ž, Weilert M, Shrikumar A, Krueger S, Alexandari A, Dalal K, Fropf R, McAnany C, Gagneur J, Kundaje A *et al* (2021) Base-resolution models of transcription-factor binding reveal soft motif syntax. *Nat Genet* 53: 354–366

Blat Y, Kleckner N (1999) Cohesins bind to preferential sites along yeast chromosome III, with differential regulation along arms versus the centric region. *Cell* 98: 249–259

Boija A, Klein IA, Sabari BR, Dall'Agnese A, Coffey EL, Zamudio AV, Li CH, Shrinivas K, Manteiga JC, Hannett NM *et al* (2018) Transcription factors activate genes through the phase-separation capacity of their activation domains. *Cell* 175: 1842–1855

Boyle AP, Davis S, Shulha HP, Meltzer P, Margulies EH, Weng Z, Furey TS, Crawford GE (2008) High-resolution mapping and characterization of open chromatin across the genome. *Cell* 132: 311–322

Buenrostro JD, Giresi PG, Zaba LC, Chang HY, Greenleaf WJ (2013) Transposition of native chromatin for fast and sensitive epigenomic profiling of open chromatin, DNA-binding proteins and nucleosome position. *Nat Methods* 10: 1213–1218

Castro-Mondragon JA, Riudavets-Puig R, Rauluseviciute I, Lemma RB, Turchi L, Blanc-Mathieu R, Lucas J, Boddie P, Khan A, Manosalva Pérez N *et al* (2022) JASPAR 2022: the 9th release of the open-access database of transcription factor binding profiles. *Nucleic Acids Res* 50: D165–D173

Chen X, Xu H, Yuan P, Fang F, Huss M, Vega VB, Wong E, Orlov YL, Zhang W, Jiang J *et al* (2008) Integration of external signaling pathways with the core transcriptional network in embryonic stem cells. *Cell* 133: 1106–1117

Cirillo LA, Lin FR, Cuesta I, Friedman D, Jarnik M, Zaret KS (2002) Opening of compacted chromatin by early developmental transcription factors HNF3 (FoxA) and GATA-4. *Mol Cell* 9: 279–289

Cox JL, Mallanna SK, Luo X, Rizzino A (2010) Sox2 uses multiple domains to associate with proteins present in Sox2-protein complexes. *PLoS One* 5: 15486

Dancy BM, Cole PA (2015) Protein lysine acetylation by p300/CBP. *Chem Rev* 115: 2419–2452

Davies JOJ, Telenius JM, McGowan SJ, Roberts NA, Taylor S, Higgs DR, Hughes JR (2015) Multiplexed analysis of chromosome conformation at vastly improved sensitivity. *Nat Methods* 13: 74–80

Davis ES, Mu W, Lee S, Dozmorov MG, Love MI, Phanstiel DH (2023) matchRanges: generating null hypothesis genomic ranges via covariate-matched sampling. *Bioinformatics* 39: btad197

De Laat W, Duboule D (2013) Topology of mammalian developmental enhancers and their regulatory landscapes. *Nature* 502: 499–506

Di Cerbo V, Mohn F, Ryan DP, Montellier E, Kacem S, Tropberger P, Kallis E, Holzner M, Hoerner L, Feldmann A *et al* (2014) Acetylation of histone H3 at lysine 64 regulates nucleosome dynamics and facilitates transcription. *Elife* 3: e01632

Dodonova SO, Zhu F, Dienemann C, Taipale J, Cramer P (2020) Nucleosome-bound SOX2 and SOX11 structures elucidate pioneer factor function. *Nature* 580: 669–672

Friedman J, Hastie T, Tibshirani R (2010) Regularization paths for generalized linear models via coordinate descent. *J Stat Softw* 33: 1–22

Friman ET, Deluz C, Meireles-Filho ACA, Govindan S, Gardeux V, Deplancke B, Suter DM (2019) Dynamic regulation of chromatin accessibility by pluripotency transcription factors across the cell cycle. *Elife* 8: e50087

Gillespie M, Jassal B, Stephan R, Milacic M, Rothfels K, Senff-Ribeiro A, Griss J, Sevilla C, Matthews L, Gong C *et al* (2022) The reactome pathway knowledgebase 2022. *Nucleic Acids Res* 50: D687–D692

Gregersen LH, Mitter R, Svejstrup JQ (2020) Using TT_{chem}-seq for profiling nascent transcription and measuring transcript elongation. *Nat Protoc* 15: 604–627

Grunstein M (1997) Histone acetylation in chromatin structure and transcription. *Nature* 389: 349–352

Hebbes TR, Thorne AW, Crane-Robinson C (1988) A direct link between core histone acetylation and transcriptionally active chromatin. *EMBO J* 7: 1395–1402

Hellman LM, Fried MG (2007) Electrophoretic mobility shift assay (EMSA) for detecting protein-nucleic acid interactions. *Nat Protoc* 2: 1849–1861

Holmberg J, Perlmann T (2012) Maintaining differentiated cellular identity. *Nat Rev Genet* 13: 429–439

Hua P, Badat M, Hanssen LLP, Hentges LD, Crump N, Downes DJ, Jeziorska DM, Oudelaar AM, Schwessinger R, Taylor S *et al* (2021) Defining genome architecture at base-pair resolution. *Nature* 595: 125–129

Huber W, Carey VJ, Gentleman R, Anders S, Carlson M, Carvalho BS, Bravo HC, Davis S, Gatto L, Girke T *et al* (2015) Orchestrating high-throughput genomic analysis with Bioconductor. *Nat Methods* 12: 115–121

Isbel L, Grand RS, Schübeler D (2022) Generating specificity in genome regulation through transcription factor sensitivity to chromatin. *Nat Rev Genet* 23: 728–740

Ishwaran H, Kogalur UB (2022) Fast unified random forests for survival, regression, and classification (RF-SRC)

Isoda T, Moore AJ, He Z, Chandra V, Aida M, Denholtz M, Piet van Hamburg J, Fisch KM, Chang AN, Fahl SP *et al* (2017) Non-coding transcription instructs chromatin folding and compartmentalization to dictate enhancer-promoter communication and T cell fate. *Cell* 171: 103–119

© 2023 The Authors

Iurlaro M, Stadler MB, Masoni F, Jagani Z, Galli GG, Schübeler D (2021) Mammalian SWI/SNF continuously restores local accessibility to chromatin. *Nat Genet* 53: 279–287

Iwafuchi-Doi M, Zaret KS (2014) Pioneer transcription factors in cell reprogramming. *Genes Dev* 28: 2679–2692

Jerabek S, Merino F, Schöler HR, Cojocaru V (2014) OCT4: dynamic DNA binding pioneers stem cell pluripotency. *Biochim Biophys Acta Gene Regul Mech* 1839: 138–154

Johnson DS, Mortazavi A, Myers RM, Wold B (2007) Genome-wide mapping of *in vivo* protein-DNA interactions. *Science* 316: 1497–1502

Kiani K, Sanford EM, Goyal Y, Raj A (2022) Changes in chromatin accessibility are not concordant with transcriptional changes for single-factor perturbations. *bioRxiv* https://doi.org/10.1101/2022.02.03.478981 [PREPRINT]

King HW, Klose RJ (2017) The pioneer factor OCT4 requires the chromatin remodeller BRG1 to support gene regulatory element function in mouse embryonic stem cells. *Elife* 6: e22631

Kubo N, Ishii H, Xiong X, Bianco S, Meitinger F, Hu R, Hocker JD, Conte M, Gorkin D, Yu M *et al* (2021) Promoter-proximal CTCF binding promotes distal enhancer-dependent gene activation. *Nat Struct Mol Biol* 28: 152–161

Kumar Mistri T, Lam CS, Arindrarto W, Rodda D, Foo YH, Ping Ng W, Ahmed S, Robson P, Wohland T (2011) Quantitative determination of Oct4-Sox2 heterodimer formation with nanog promoter element. *Biophys J* 100: 74a

Kumar V, Rayan NA, Muratani M, Lim S, Elanggovan B, Xin L, Lu T, Makhija H, Poschmann J, Lufkin T *et al* (2016) Comprehensive benchmarking reveals H2BK20 acetylation as a distinctive signature of cell-state-specific enhancers and promoters. *Genome Res* 26: 612–623

Lawrence M, Gentleman R, Carey V (2009) rtracklayer: an R package for interfacing with genome browsers. *Bioinformatics* 25: 1841–1842

Lawrence M, Huber W, Pagès H, Aboyoun P, Carlson M, Gentleman R, Morgan M, Carey V (2013) Software for computing and annotating genomic ranges. *PLoS Comput Biol* 9: e1003118

Li H, Durbin R (2009) Fast and accurate short read alignment with Burrows-Wheeler transform. *Bioinformatics* 25: 1754–1760

Li H, Handsaker B, Wysoker A, Fennell T, Ruan J, Homer N, Marth G, Abecasis G, Durbin R, 1000 Genome Project Data Processing Subgroup (2009) The sequence alignment/map format and SAMtools. *Bioinformatics* 25: 2078–2079

Li D, Liu J, Yang X, Zhou C, Guo J, Wu C, Qin Y, Guo L, He J, Yu S *et al* (2017) Chromatin accessibility dynamics during iPSC reprogramming. *Cell Stem Cell* 21: 819–833

Li D, Shu X, Zhu P, Pei D (2021) Chromatin accessibility dynamics during cell fate reprogramming. *EMBO Rep* 22: e51644

Liu T (2014) Use model-based analysis of ChIP-Seq (MACS) to analyze short reads generated by sequencing protein–DNA interactions in embryonic stem cells. *Methods Mol Biol* 1150: 81–95

Liu NQ, Maresca M, van den Brand T, Braccioli L, Schijns MMGA, Teunissen H, Bruneau BG, Nora EP, de Wit E (2020) WAPL maintains a cohesin loading cycle to preserve cell-type-specific distal gene regulation. *Nat Genet* 53: 100–109

Lodato MA, Ng CW, Wamstad JA, Cheng AW, Thai KK, Fraenkel E, Jaenisch R, Boyer LA (2013) SOX2 co-occupies distal enhancer elements with distinct POU factors in ESCs and NPCs to specify cell state. *PLoS Genet* 9: e1003288

Love MI, Huber W, Anders S (2014) Moderated estimation of fold change and dispersion for RNA-seq data with DESeq2. *Genome Biol* 15: 550

Marson A, Levine SS, Cole MF, Frampton GM, Brambrink T, Johnstone S, Guenther MG, Johnston WK, Wernig M, Newman J *et al* (2008) Connecting microRNA genes to the core transcriptional regulatory circuitry of embryonic stem cells. *Cell* 134: 521–533

Martinez-Ara M, Comoglio F, van Arensbergen J, van Steensel B (2022) Systematic analysis of intrinsic enhancer-promoter compatibility in the mouse genome. *Mol Cell* 82: 2519–2531

McLean CY, Bristor D, Hiller M, Clarke SL, Schaar BT, Lowe CB, Wenger AM, Bejerano G (2010) GREAT improves functional interpretation of cis-regulatory regions. *Nat Biotechnol* 28: 495–501

Miao L, Tang Y, Bonneau AR, Chan SH, Kojima ML, Pownall ME, Vejnar CE, Gao F, Krishnaswamy S, Hendry CE *et al* (2022) The landscape of pioneer factor activity reveals the mechanisms of chromatin reprogramming and genome activation. *Mol Cell* 82: 986–1002

Michael AK, Thomä NH (2021) Reading the chromatinized genome. *Cell* 184: 3599–3611

Michael AK, Grand RS, Isbel L, Cavadini S, Kozicka Z, Kempf G, Bunker RD, Schenk AD, Graff-Meyer A, Pathare GR *et al* (2020) Mechanisms of OCT4-SOX2 motif readout on nucleosomes. *Science* 368: 1460–1465

Nabet B, Roberts JM, Buckley DL, Paulk J, Dastjerdi S, Yang A, Leggett AL, Erb MA, Lawlor MA, Souza A *et al* (2018) The dTAG system for immediate and target-specific protein degradation. *Nat Chem Biol* 14: 431–441

O'Leary NA, Wright MW, Brister JR, Ciufo S, Haddad D, McVeigh R, Rajput B, Robbertse B, Smith-White B, Ako-Adjei D *et al* (2016) Reference sequence (RefSeq) database at NCBI: current status, taxonomic expansion, and functional annotation. *Nucleic Acids Res* 44: D733–D745

Pastor WA, Liu W, Chen D, Ho J, Kim R, Hunt TJ, Lukianchikov A, Liu X, Polo JM, Jacobsen SE *et al* (2018) TFAP2C regulates transcription in human naive pluripotency by opening enhancers. *Nat Cell Biol* 20: 553–564

Pijuan-Sala B, Wilson NK, Xia J, Hou X, Hannah RL, Kinston S, Calero-Nieto FJ, Poirion O, Preissl S, Liu F *et al* (2020) Single-cell chromatin accessibility maps reveal regulatory programs driving early mouse organogenesis. *Nat Cell Biol* 22: 487–497

Pintacuda G, Wei G, Roustan C, Kirmizitas BA, Solcan N, Cerase A, Castello A, Mohammed S, Moindrot B, Nesterova TB *et al* (2017) hnRNPK recruits PCGF3/5-PRC1 to the xist RNA B-repeat to establish polycomb-mediated chromosomal silencing. *Mol Cell* 68: 955–969

Pradeepa MM, Grimes GR, Kumar Y, Olley G, Taylor GCA, Schneider R, Bickmore WA (2016) Histone H3 globular domain acetylation identifies a new class of enhancers. *Nat Genet* 48: 681–686

Ptashne M, Gann A (1997) Transcriptional activation by recruitment. *Nature* 386: 569–577

Ren B, Robert F, Wyrick JJ, Aparicio O, Jennings EG, Simon I, Zeitlinger J, Schreiber J, Hannett N, Kanin E *et al* (2000) Genome-wide location and function of DNA binding proteins. *Science* 290: 2306–2309

Rinzema NJ, Sofiadis K, Tjalsma SJD, Verstegen MJAM, Oz Y, Valdes-Quezada C, Felder A-K, Filipovska T, van der Elst S, de Andrade dos Ramos Z *et al* (2021) Building regulatory landscapes: enhancer recruits cohesin to create contact domains, engage CTCF sites and activate distant genes. *bioRxiv* https://doi.org/10.1101/2021.10.05.463209 [PREPRINT]

Ritz C, Baty F, Streibig JC, Gerhard D (2015) Dose-response analysis using R. *PLoS One* 10: e0146021

Roberts GA, Ozkan B, Gachulincová I, O'Dwyer MR, Hall-Ponsele E, Saxena M, Robinson PJ, Soufi A (2021) Dissecting OCT4 defines the role of nucleosome binding in pluripotency. *Nat Cell Biol* 23: 834–845

Robertson G, Hirst M, Bainbridge M, Bilenky M, Zhao Y, Zeng T, Euskirchen G, Bernier B, Varhol R, Delaney A *et al* (2007) Genome-wide profiles of STAT1

© 2023 The Authors

DNA association using chromatin immunoprecipitation and massively parallel sequencing. *Nat Methods* 4: 651–657
Schep A (2020) motifmatchr: fast motif matching in R
Schick S, Grosche S, Kohl KE, Drpic D, Jaeger MG, Marella NC, Imrichova H, Lin JMG, Hofstätter G, Schuster M *et al* (2021) Acute BAF perturbation causes immediate changes in chromatin accessibility. *Nat Genet* 53: 269–278
Sinha KK, Bilokapic S, Du Y, Malik D, Halic M (2023) Histone modifications regulate pioneer transcription factor cooperativity. *Nature* 619: 378–384
Solomon MJ, Larsen PL, Varshavsky A (1988) Mapping proteinDNA interactions *in vivo* with formaldehyde: evidence that histone H4 is retained on a highly transcribed gene. *Cell* 53: 937–947
Soufi A, Garcia MF, Jaroszewicz A, Osman N, Pellegrini M, Zaret KS (2015) Pioneer transcription factors target partial DNA motifs on nucleosomes to initiate reprogramming. *Cell* 161: 555–568
Stormo GD, Zhao Y (2010) Determining the specificity of protein–DNA interactions. *Nat Rev Genet* 11: 751–760
Swinstead EE, Paakinaho V, Presman DM, Hager GL (2016) Pioneer factors and ATP-dependent chromatin remodeling factors interact dynamically: a new perspective: multiple transcription factors can effect chromatin pioneer functions through dynamic interactions with ATP-dependent chromatin remodeling factors. *Bioessays* 38: 1150–1157
Takahashi K, Yamanaka S (2006) Induction of pluripotent stem cells from mouse embryonic and adult fibroblast cultures by defined factors. *Cell* 126: 663–676
Thomas HF, Kotova E, Jayaram S, Pilz A, Romeike M, Lackner A, Penz T, Bock C, Leeb M, Halbritter F *et al* (2021) Temporal dissection of an enhancer cluster reveals distinct temporal and functional contributions of individual elements. *Mol Cell* 81: 969–982
Todd CD, Deniz Ö, Taylor D, Branco MR (2019) Functional evaluation of transposable elements as enhancers in mouse embryonic and trophoblast stem cells. *Elife* 8: e44344
Vaquerizas JM, Kummerfeld SK, Teichmann SA, Luscombe NM (2009) A census of human transcription factors: function, expression and evolution. *Nat Rev Genet* 10: 252–263
Verma R, Mohl D, Deshaies RJ (2020) Harnessing the power of proteolysis for targeted protein inactivation. *Mol Cell* 77: 446–460
Xiao L, Parolia A, Qiao Y, Bawa P, Eyunni S, Mannan R, Carson SE, Chang Y, Wang X, Zhang Y *et al* (2022) Targeting SWI/SNF ATPases in enhancer-addicted prostate cancer. *Nature* 601: 434–439
Zacher B, Ertl J, Campos-Martin R, Gagneur J, Tresch A (2020) STAN: the genomic STate ANnotation package
Zaret KS, Carroll JS (2011) Pioneer transcription factors: establishing competence for gene expression. *Genes Dev* 25: 2227–2241
Zaret KS, Mango SE (2016) Pioneer transcription factors, chromatin dynamics, and cell fate control. *Curr Opin Genet Dev* 37: 76–81
Zheng R, Wan C, Mei S, Qin Q, Wu Q, Sun H, Chen C-H, Brown M, Zhang X, Meyer CA *et al* (2019) Cistrome Data Browser: expanded datasets and new tools for gene regulatory analysis. *Nucleic Acids Res* 47: D729–D735
Zuin J, Roth G, Zhan Y, Cramard J, Redolfi J, Piskadlo E, Mach P, Kryzhanovska M, Tihanyi G, Kohler H *et al* (2022) Nonlinear control of transcription through enhancer–promoter interactions. *Nature* 604: 571–577

License: This is an open access article under the terms of the Creative Commons Attribution License, which permits use, distribution and reproduction in any medium, provided the original work is properly cited.

Expanded View Figures

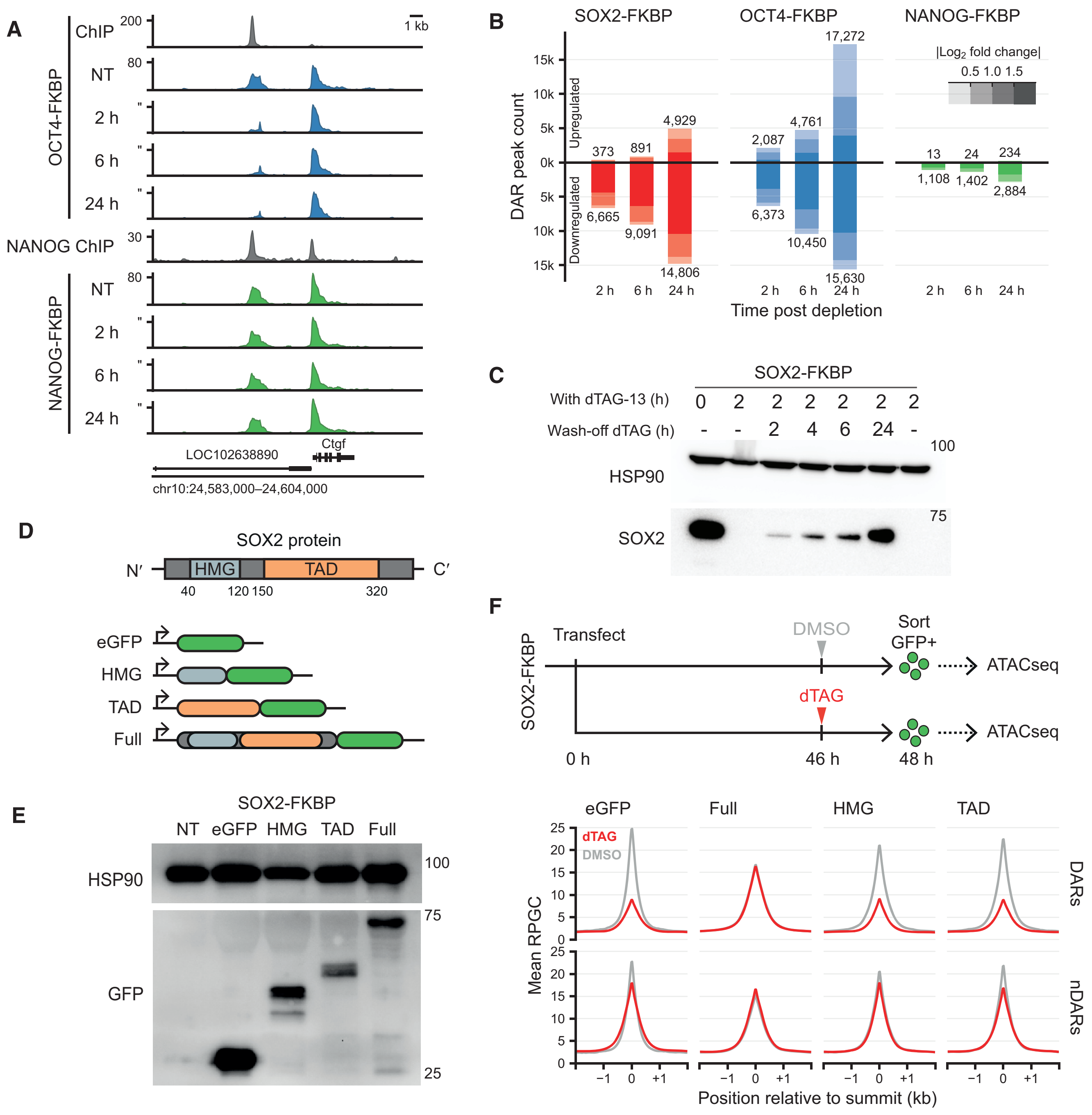

Figure EV1.

 © 2023 The Authors

Figure EV1. Loss of accessibility after pioneer factor loss.

A Genomic tracks of accessibility changes by ATACseq in OCT4 and NANOG FKBP tagged lines measured in untreated condition (NT) and after OCT4 and NANOG depletion at the indicated timepoints. ChIPseq tracks for OCT4 in the OCT4-FKBP line and NANOG from publicly available data are shown on top of ATACseq. *Y*-axes refers to reads per genomic content (RPGC).
B Bar plot showing the number of differential accessible regions (DARs) after a time course of SOX2, OCT4 and NANOG depletion, in FKBP tagged lines.
C Western blot analysis of SOX2 expression level at 0 and 2 h of dTAG-13 and during a time course of dTAG-13 washoff. HSP90 was used as loading control.
D Schematic representation of SOX2 protein showing the DNA binding domain (HMG) and the trans-activation domain (TAD). To generate versions of the protein for ectopic expression, the HMG, TAD or full length SOX2 was cloned in frame with the eGFP sequence in an episomal expression vector.
E Western blot using an antibody against eGFP shows the fusion proteins running at the expected size. HSP90 was used as a loading control.
F Top; Representation of the experimental procedure for ATACseq after ectopic expression of the truncated SOX2-EGFP constructs. SOX2-FKBP cells were transfected with the plasmids and seeded for DMSO or dTAG-13 treatment. eGFP positive cells were sorted and ATACseq was performed on the eGFP expressing cells. Bottom; Line plot showing the average signal of ATACseq after overexpression of the plasmids after DMSO (gray) or dTAG13 treatment (red) for the endogenous SOX2-FKBP degradation. The average ATACseq signal is plotted for the DARs and nDARs as identified in Fig 1F.

Source data are available online for this figure.

Figure EV2. Quantitative and qualitative analysis of ATACseq and ChIPseq and Random forest classification reveals proteins and histone modifications that can predict differential accessibility.

A Euler diagram showing the overlap between differentially accessible regions (DARs) after 2 h of SOX2 depletion, all ATACseq peaks (OCR, open chromatin regions) and SOX2 DNA binding (SOX2 ChIP). Bottom panel shows the number of peaks in each overlap category. CCR: closed chromatin region, i.e., SOX2 ChIPseq peaks that do not overlap OCRs.
B Fraction of peaks containing 1 or more OCT4::SOX2 or SOX2 DNA binding motifs, stratified by whether OCRs, DARs and/or SOX2 binding sites as measured by ChIPseq, or combinations thereof.
C Vertical histogram of SOX2 ChIPseq peaks ranked by signal intensity, stratified by their overlap with downregulated DARs or lack of such overlap (nDAR), displayed in 200 peak bins.
D Left, top 25 (chromatin binding) factors in the Cistrome factors datasets whose overlap with all ATACseq peaks is predictive in random forest classification to discriminate the DARs from non-DARs (nDAR) peaks partially matched for SOX2 binding levels. ATACseq peaks were extended by 300 bp in both directions. Variable importance was calculated with subsampling inference, wherein the 95% confidence interval (CI) is indicated with a light color, the 50% CI with a darker color and the median with a point. Enrichment and depletion indicate higher and lower average overlap in the DAR than nDAR categories respectively. Right, top 25 histone modifications using the Cistrome histone datasets of 100 re-sampling.
E Tornado plots showing example differences between DARs and nDARs for SS18, CTCF, H3K79me2 and H3K64ac from publicly available ChIPseq datasets. Coverage indicates values in pre-processed data.
F Heatmap of ChromHMM defining chromatin states of different set of ATACseq peaks: other open chromatin regions (OCRs), DARs and partially SOX2-binding matched nDARs. The expected value was calculated under independence of proportions assumption, as they are calculated for a chi-squared test.

© 2023 The Authors

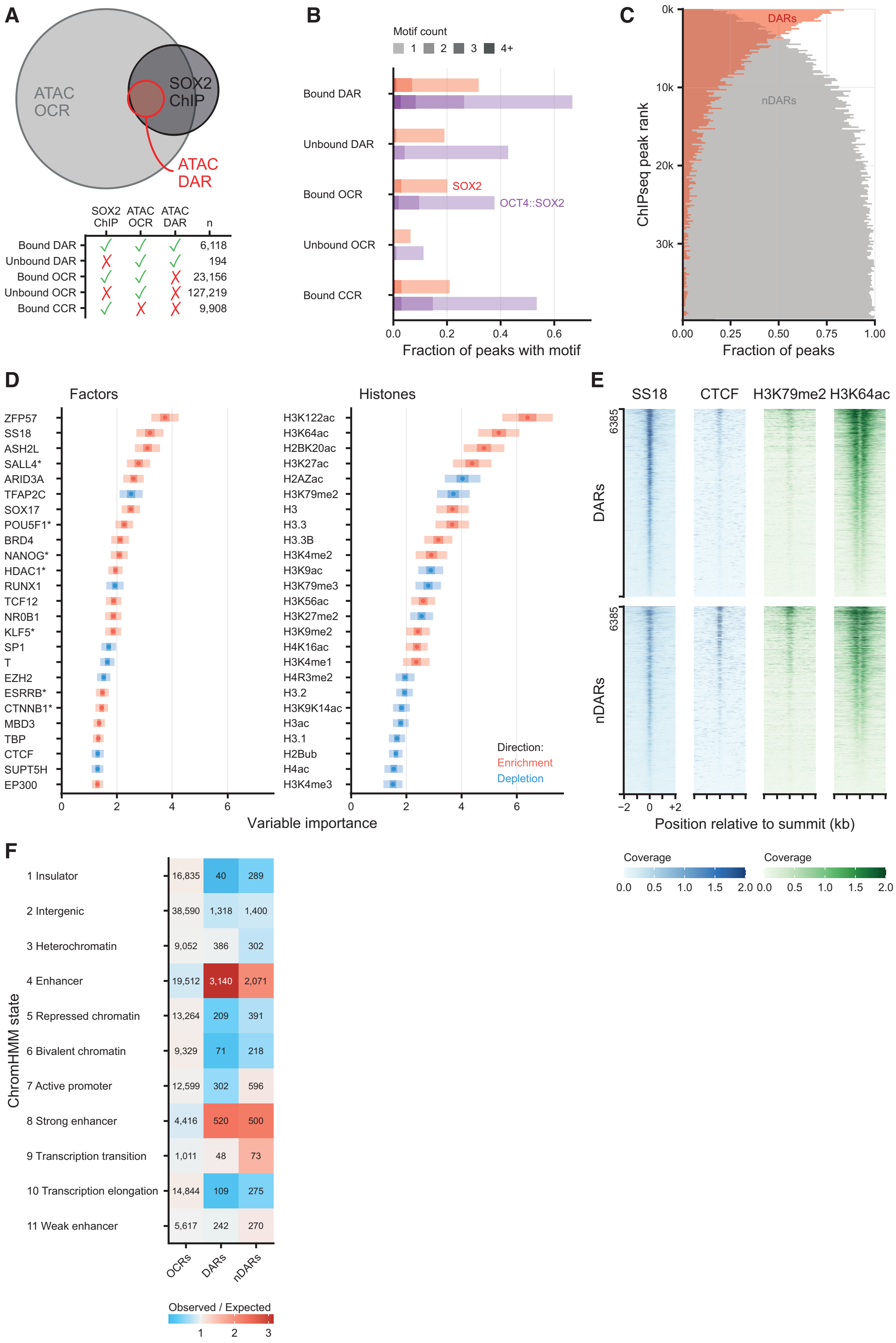

Figure EV2.

 © 2023 The Authors

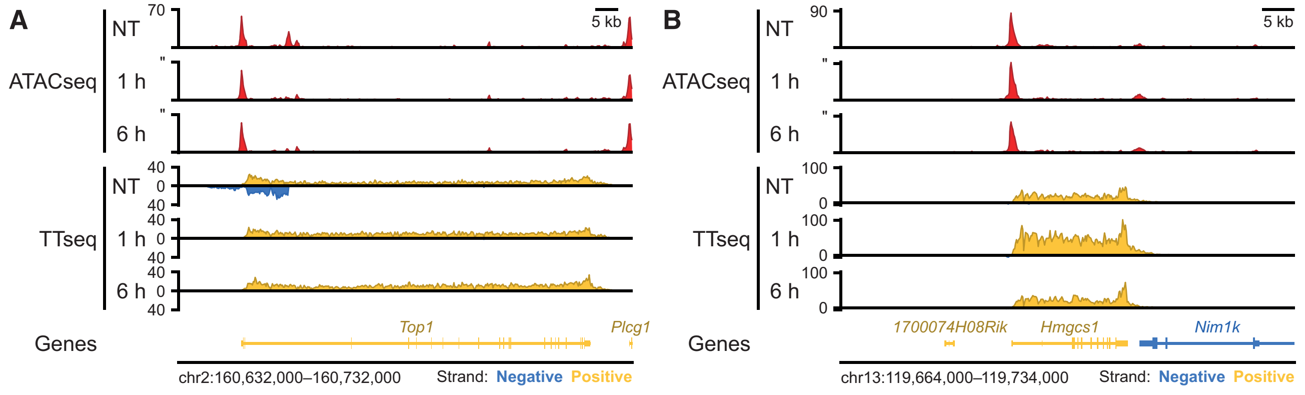

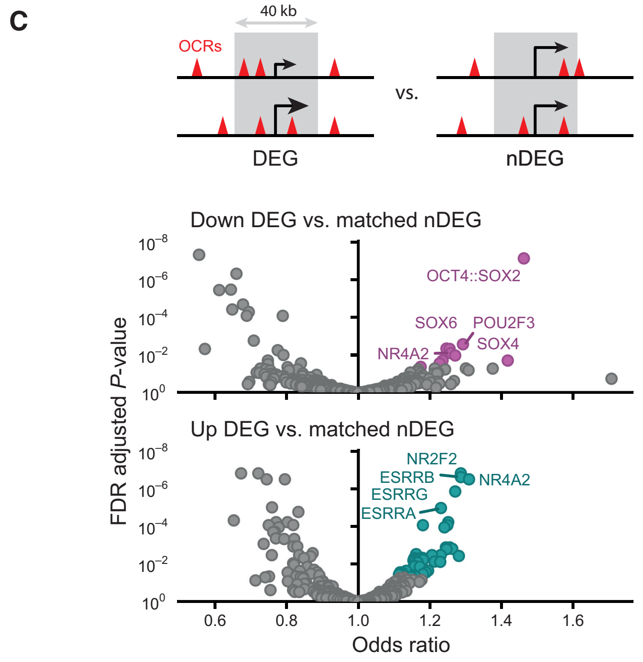

D

Pathway	Foreground Ratio	Background Ratio	*P*-value FDR adjusted
**1H Up DEGs**			
Cholesterlos biosynthesis	20/320	25/5704	$1.4 \cdot 10^{-18}$
Metabolism of steroids	23/320	77/5704	$3.7 \cdot 10^{-9}$
Metabolism of lipids	49/320	392/5704	$1.0 \cdot 10^{-5}$
Hemostasis	39/320	321/5704	$4.3 \cdot 10^{-4}$
Cardiac conduction	12/320	57/5704	$7.2 \cdot 10^{-3}$
**2H Up DEGs**			
Cholesterol biosynthesis	20/602	25/5704	$5.7 \cdot 10^{-13}$
Metabolism of steroids	24/602	77/5704	$2.3 \cdot 10^{-4}$
Cardiac conduction	16/602	57/5704	$4.8 \cdot 10^{-2}$
**6H Up DEGs**			
Ion homeostasis	15/665	36/5704	$2.7 \cdot 10^{-3}$
RHO GTPase cycle	63/665	319/5704	$2.7 \cdot 10^{-3}$
Cardiac conduction	19/665	57/5704	$2.7 \cdot 10^{-3}$
RAC1 GTPase cycle	31/665	121/5704	$2.7 \cdot 10^{-3}$
Muscle contraction	23/665	86/5704	$1.4 \cdot 10^{-4}$

Figure EV3. Upregulation of transcripts following SOX2 depletion.

A Example region showing changes in accessibility measured by ATACseq and transcription measured by TT_{chem}seq for the *Top1* locus in untreated (NT), 1 and 6 h of dTAG-13 treatment in SOX2-FKBP cells. *Y*-axes shows reads per genomic content.

B Same as in (A) but for *Hmgcs1* locus.

C Top: schematic of the widow selected for motif analysis around differentially expressed genes (DEGs) and expressed matched control non-DEGs (nDEGs). Bottom: volcano plot showing the motifs found in open chromatin regions (OCRs) in a 40 kb window centered on the TSSs of downregulated DEGs and at upregulated DEGs.

D Table showing reactome pathway (Gillespie *et al*, 2022) overrepresentation analysis results on TT_{chem}-seq DEGs.following SOX2 depletion. Table shows all significant hits at the FDR adjusted *P*-value threshold lower than 0.05, stratified by timepoint and direction. For 0.5 h and downregulated DEGs, no significant pathway enrichments were found.

© 2023 The Authors

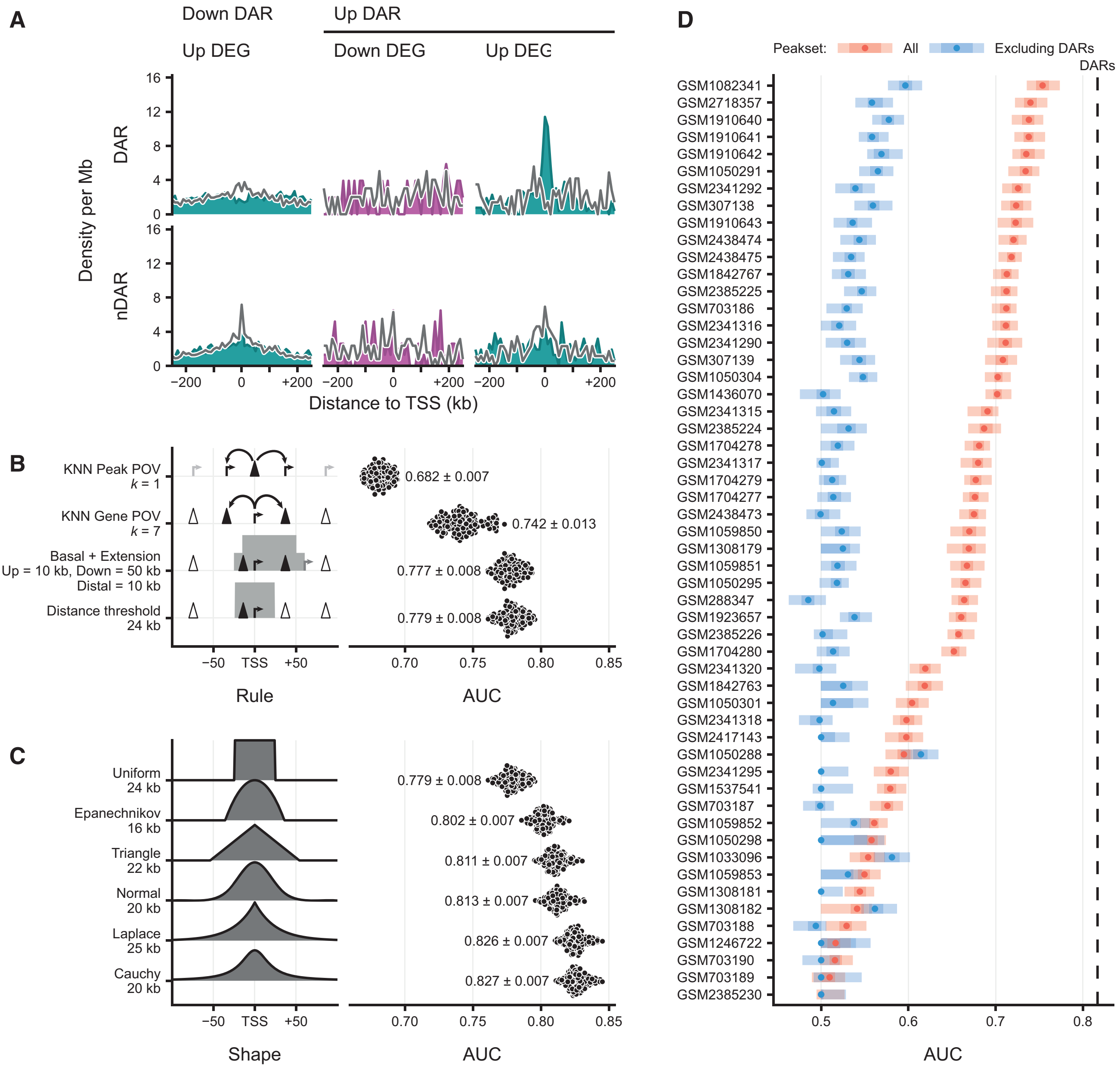

Figure EV4. Characterization of different association rules and chromatin features for the prediction of transcriptional changes.

A Density of peaks in 10 kb bins nearby transcription start sites (TSS) of upregulated (light blue) and downregulated (purple) differentially expressed genes (DEGs) and their expression-matched stable control (gray) non-DEGs (nDEGs). Panels indicate the set of peaks that were aligned to the TSS: down differentially accessible regions (DARs) and up DARs. Bottom row shows these densities for an equal number of stable non-DARs (nDAR) at the same gene sets.

B Predictive performance comparison of different association rules to discriminate downregulated DEGs upon SOX2 depletion at 2 h from matched nDEGs, with counts of associated (n)DAR peaks as predictors. *X*-axis metric notes the area under the receiver operator characteristic curve (AUC). Dots represent 100 re-samplings of the matched nDEGs. Numbers represent mean ± standard deviation. Optimal parameters for every rule indicated at the *Y*-axis labels, such as distance = 25 kb and k = 6, were chosen by performing a parameter sweep and choosing the parameter that minimized cross-validation error. Left part visually indicates association rule.

C Like (B), but for various kernel-based weighting functions instead of association rules, and weighted sums of peaks instead of counts as predictors. Left part gives visual indication of kernel shape. Numbers represent mean ± standard deviation.

D Predictive power on expression changes based on the many publicly available ChIPseq data, with or excluding peaks overlapping with DARs, for SOX2 in mESC-like cells (via Cistrome). The dotted line indicate the average predictive power of DARs for context. *Y*-axis gives Gene Expression Omnibus accession numbers for the datasets. *X*-axis metric notes the area under the receiver operator characteristic curve (AUC). Intervals and medians were calculated for 100 re-samplings of the matched nDEGs. The light shade gives the 95% inter-percentile range (IPR), the darker shade gives the inter-quartile range and the dots give the medians.

Source data are available online for this figure.

 © 2023 The Authors

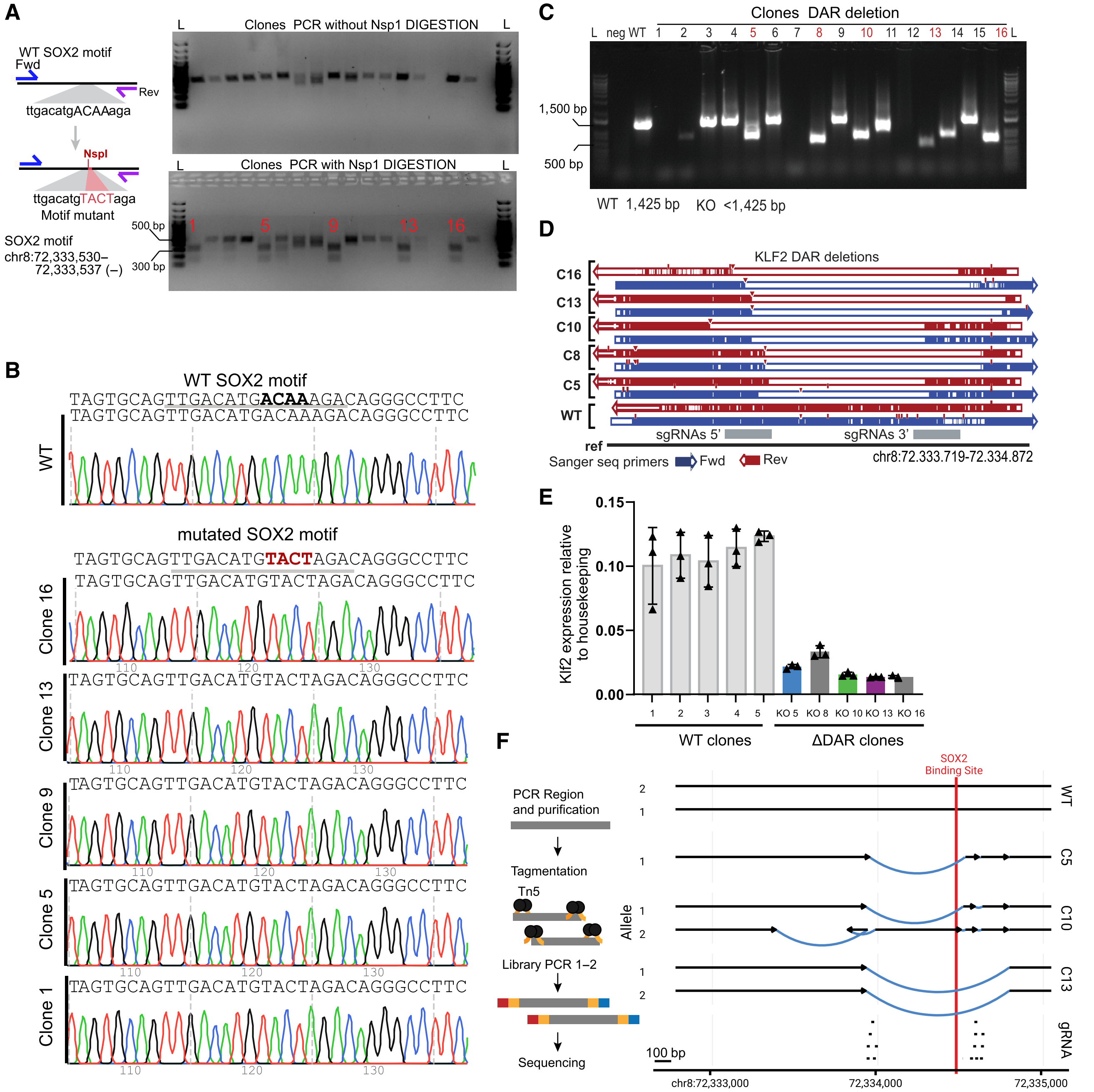

Figure EV5. Validation of genome editing of DAR KO and *Klf2* gene expression.

A Left: schematic of the procedure used for editing and selection of clones with homozygous mutation of the motif. Right: Top, DNA gel electrophoresis of the PCR product related to region selected for point mutant. Bottom, digestion of the PCR product (top) using NspI. Homozygous clones show 2 bands. Unedited clones show 1 band. L, ladder.

B Sanger tracks for WT and motif mutant clones.

C Gel electrophoresis of PCR for genotyping disruption of the DAR region in clones from the gene edited SOX2-FKBP parental cell line. Primers amplifying the targeted regions were used to control for the homozygous disruption compared to WT amplification. L: ladder, Neg: water control, DAR KO clones: clones selected for genotyping. In red, clones selected for further experiments.

D Validation of the disruption using Sanger sequencing in clones compared to non-edited clones. Blue: forward primer, red: reverse primer, gray: region targeted by sgRNAs 5′ and 3′ of the DAR.

E RT–qPCR of *Klf2* expression, similar as Fig 5B, but using an alternative set of primers, in 5 parental clones and the DAR KO clones. Expression is relative to housekeeping gene *Rsp26*. Error bar represent standard deviation of three biological replicates.

F Left panel shows simplified overview of the amplicon sequencing procedure. Right panel shows the most likely assembly based on the amplicon sequencing of the DAR regions in WT and KO clones. Region targeted by sgRNAs are shown at the bottom. Arrows indicate the centromere to telomere orientation. Black lines shows assembled sequence that is identical to the reference sequence. Blue lines show structural variants identified in the clones. Red indicates position of the SOX2 binding motif overlapping with a SOX2 ChIPseq peak.

© 2023 The Authors

APPENDIX

Pioneer activity distinguishes activating from non-activating SOX2 binding sites

Michela Maresca[1,3], Teun van den Brand[1,3], Hangpeng Li[2], Hans Teunissen[1], James Davies[2], Elzo de Wit[1,*]

Table of contents:

Appendix Figure S1: Appendix Figure S1: Granular motif analysis for SOX2 and OCT4 depletion.

Appendix Figure S1

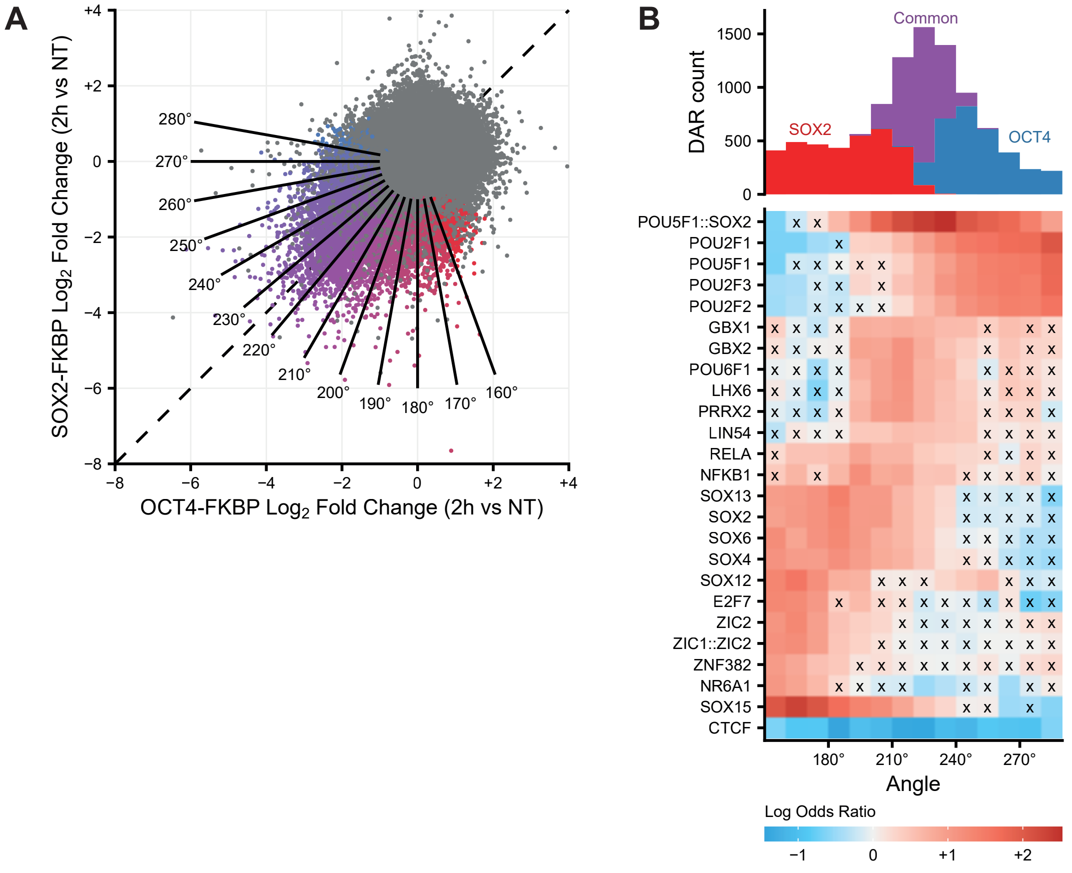

Appendix Figure S1: Granular motif analysis for SOX2 and OCT4 depletion.

A) Scatterplot showing ATACseq log_2 fold changes upon 2h of depletion of OCT4 (x-axis) and SOX2 (y-axis). Peaks that are downregulated differentially accessible regions (DARs) in either depletion are coloured according to angular bins, representing bins where the relative direction of change between the two depletions are similar. Breaks between angular bins are indicated with segments and their angles. B) Top: histogram of DAR categories from figure 2 by angular bin. Bottom: heatmap of motif enrichment analysis by angular bin, wherein effect size is indicated by log odds ratios of finding the motif in the bin relative to all other OCRs. Crosses denote non-significant depletions or enrichments.
