# Supplementary material for: Pioneer activity distinguishes activating from non‐activating SOX2 binding sites
Source: EMBO J. 2023 Sep 11;42(20):e113150. doi: 10.15252/embj.2022113150 (PMC10577566; doi:10.15252/embj.2022113150)
Supplement: Supplementary file 8 [file EMBJ-42-e113150-s006.pdf]

THE EMBO JOURNAL

Pioneer activity distinguishes activating from non-activating SOX2 binding sites

Michela Maresca, Teun van den Brand, Hangpeng Li, Hans Teunissen, James Davies and Elzo de Wit

DOI: 10.15252/embj.2022113150

Corresponding author(s): Elzo de Wit (e.d.wit@nki.nl)

Review Timeline:

Submission Date:	25th Nov 22
Editorial Decision:	13th Feb 23
Revision Received:	13th Jun 23
Editorial Decision:	8th Jul 23
Revision Received:	17th Jul 23
Accepted:	22nd Jul 23

Editor: Kelly Anderson

Transaction Report:

(Note: With the exception of the correction of typographical or spelling errors that could be a source of ambiguity, letters and reports are not edited. Depending on transfer agreements, referee reports obtained elsewhere may or may not be included in this compilation. Referee reports are anonymous unless the Referee chooses to sign their reports.)

Dear Dr. de Wit,

Thank you for submitting your manuscript for consideration by the EMBO Journal. It has now been seen by three referees whose comments are shown below.

Should you be able to address their criticisms in full, we could consider a revised manuscript. It is EMBO Journal policy to allow a single round of revision only and that, therefore, acceptance or rejection of the manuscript will depend on the completeness of your responses in this revised version. It would be good to discuss your plan to address the referee concerns and I will be available to do so via email or zoom in the coming weeks.

Along with your revised manuscript, please include a detailed point-by-point response to the referees' comments. Please bear in mind that this will form part of the Review Process File, and will therefore be available online to the community. For more details on our Transparent Editorial Process, please visit our website: https://www.embo.org/embo-press I have included a guide to revisions for your convenience.

We generally allow three months as standard revision time. As a matter of policy, competing manuscripts published during this period will not negatively impact on our assessment of the conceptual advance presented by your study. However, we request that you contact the editor as soon as possible upon publication of any related work, to discuss how to proceed. Should you foresee a problem in meeting this three-month deadline, please let us know in advance and we may be able to grant an extension.

Thank you for the opportunity to consider your work for publication. I look forward to your revision.

Yours sincerely,

Kelly

Kelly M Anderson, PhD
Editor
The EMBO Journal
k.anderson@embojournal.org

Further information is available in our Guide For Authors: https://www.embopress.org/page/journal/14602075/authorguide

The revision must be submitted online within 90 days; please click on the link below to submit the revision online before 14th May 2023.

Referee #2:

In the paper by Maresca et al the authors utilize a degradation tag system to examine how an acute depletion of SOX2 in mouse ESCs, affects chromatin accessibility and gene expression. Two classes of SOX2 bound DNA-regions are identified; regions that become less accessible after SOX2 depletion and regions that remain open. SOX2 depletion results in a similar set of genes that are down- and upregulated. Finally, by comparing SOX2 binding to chromatin accessibility and gene transcription, the authors conclude that genomic regions that are dependent on SOX2 for their accessibility are more significant for SOX2 mediated gene activation, compared to accessible chromatin regions that are independent of SOX2.

The ability to rapidly deplete SOX2 protein expression provides a powerful system to examine the role of SOX2 in the regulation of chromatin accessibility and gene expression. One interesting finding is that SOX2 binding combined with chromatin accessibility is a better predictor of transcriptional regulation than SOX2 ChIP-seq data alone. However, the function of SOX2 as a pioneer factor has been established in several previous publications. Therefore, my major concern regards the general novelty and interest of their findings.

Specific comments:
1) Papers demonstrating the role of SOX2 as a pioneer factor should be properly referenced and discussed.

2) Like SOX2 depletion, OCT4 depletion leads to a loss of open chromatin regions. The authors should examine SOX2

expression in cells depleted for OCT4.

3) As shown in Fig. 1, open chromatin regions are restored by washing-out the dTAG molecule after 2 hrs. The authors should examine how well SOX2 expression is restored following dTAG wash-out? The authors should determine if the same DNA-regions are "re-opened" after the dTAG wash-out as those closed after SOX2 depletion?

4) In Fig. S1 the authors show that only full-length SOX2 can maintain open chromatin, and not the HMG or transactivation domain in isolation. The authors should discuss this finding in relation to previous publications showing that the HMG-domain of SOX2 has a pioneer function (e.g Dodonova et al., 2020).

5) In Fig. S2 the authors define SOX2 bound regions using ChIP-seq. To validate the quality of the ChIP the authors should present centrally enriched motifs within DNA regions suggested to be bound by SOX2. The authors should explain/discuss the strong enrichment of SOX2 motifs in unbound DARs compared to for instance bound OCRs (Fig. S2B).

6) In Fig. 2 the authors show that the chromatin accessibility is decreased more rapidly upon SOX2 depletion in DNA regions enriched for SOX motifs. Are regions in which the accessibility is decreased more slowly bound by SOX2, or is the decreased accessibility a secondary effect of SOX2 depletion? Is the expression of transcription factors proposed to bind slowly changing chromatin regions, e.g. KLF4, altered by SOX2 depletion?

Referee #3:

This is a very interesting study using acute protein degradation and multiple epigenomic assays to investigate the mechanisms that decide functionality of Sox2 binding sites in mouse embryonic stem cells. The authors found that Sox2 affects chromatin accessibility at a subset of its binding sites, which decides their gene regulation function. The manuscript is well written and the figures are of high quality. That being said, I have a few concerns/comments for the manuscript, particularly for the experimental validation part.

1. Figure 1F. It seems that DAR has stronger SOX2 binding than nDAR, which should be quantified. Does SOX2 binding intensity/frequency decides the pioneer activity and functionality, especially for the distal enhancer sites?

2. It would be interesting to compare the DAR sites regulated by Sox2 and Oct4. Do they overlap with each other? And what are the motifs enriched at the unique and shared DAR sites of Sox2 and Oct4?

3. For gene expression results, while SOX2 mainly activates its sites, the gene expression responses are seem in both directions (up and down) based on Figure 3A. It would be interesting to explore/discuss the mechanisms of how genes are upregulated in response to Sox2 degradation. Also, I would recommend compare the genes expression changes in response to acute Sox2 degradation to those previously reported from traditional Sox2 knockdown assays in mESC and show how different they could be. In addition, it would be interesting to present pathways that these acute responsive genes are enriched in.

4. I am somehow confused of Figure 4A. If Sox2 DAR sites are mostly distal enhancers (based on ChromHMM results), how come they are enriched at TSS regions of DEG? Was this due to the 10kb bins that the authors used in the figure? Would a higher resolution plot in a smaller window centered at TSS work better?

5. The experimental validation needs more work in my opinion. First, for the klf2 locus, there are two additional SOX2 DAR sites upstream of Klf2, which should be included in the validation (the authors already discussed some hypothesis for them, which need to be tested). Second, deletion of the entire DAR site may affect many other bound transcription factors. How would we know the effects come from Sox2? Could the authors just cut (by one guide) or delete the SOX motif in the site? or there are multiple SOX motifs within it, making it difficult to choose where to cut? Finally, I think including additional example(s) for validation, especially for genes that have nDAR Sox2 sites, would strengthen the conclusion a lot.

Some minor points:
- Figure S2E listed 5461 DAR and 5488 nDAR sites, which are different from the numbers shown in Figure 1F. What are the differences behind them?
- It seems from Fig S2B that unbound DARs are also enriched with SOX2/OCT4:SOX2 as compared to unbound OCRs. Are these sites also enriched with Sox2 binding (based on ChIP-seq signal) but were not called as peaks?
- Sox2 ChIP-seq tracks should be added to Figures 1D, 2A, 3C, S1A. S1A also needs Oct4 and Nanog tracks.

Referee #4:

Maresca and colleagues describes the effect on chromatin accessibility and nascent transcription after acute SOX2 degradation in murine ES cells. The short time-course reveals distinct SOX2 regulomes divided into SOX2 pioneering binding sites where chromatin is open and SOX2 binding sites where chromatin accessibility is not affected. To further elucidate the effect of SOX2 pioneering activity on chromatin opening, the authors focused on two SOX2 bound regulatory sequences in the Klf2 locus that they had showed interacted with the Klf2 promoter. CRISPR edited mutations of the SOX2 binding site in the downstream regulatory region reduced chromatin accessibility and abrogated Klf2 expression.

In general, this is a valuable piece of work as it provides detailed characterisation of SOX2 and its pioneering activity in mES cells. The manuscript is well written, and the figures are nice and clear. The study addresses some of the limitations of the Friman et al., Elife 2019 study, where time for reduction of SOX2 was much longer (towards 40 hours). The acute SOX2 protein degradation observed here has improved the elucidation of the pioneering function of this TF. However, I have some concerns that I wish that the authors should address which are listed below.

Major comments:

1. As SOX2 protein levels have been shown to affect OCT4 levels (Strebinger et al., MolSystBiol 2019), a western representing the OCT4 protein during the time-course of SOX2-FKBP degradation should be shown (Figure 1B). If OCT4 protein levels are affected this should be discussed. SOX2 and OCT4 binds as heterodimers at closed chromatin (Soufi et al., Cell 2015 and Echigoya et al., SciRep 2020). It is therefore pertinent that the authors show and discuss what effect acute SOX2 depletion have on global OCT4 enrichment. A ChIP-seq of OCT4 should be conducted upon acute SOX2 kd time course incubation with dTag-13. With the acute knockdown of SOX2 and OCT4, can the authors re-examine the cooperational relationship between SOX2 and OCT4 on chromatin accessibility as has been addressed in Friman et al., Elife 2019?
2. Furthermore, to follow up on the point above, for Figure 1E, washout and restoration of chromatin opening should be complemented with a western blot to show SOX2, OCT4 and loading control protein levels.
3. Cistrome scoring analysis: Figure S2D is not referred to in the text, please revise . It is interesting that OCT4 and NANOG are relatively high up on the list as "Enriched" and that these are also known to interact with SOX2. SALL4 has also been shown to interact with SOX2 and OCT4 (Tanimura et al., JBC 2013) and should be discussed. Can Maresca and colleagues identify other SOX2 interacting proteins among the "Enriched" versus "Depleted" factors?
4. As you have described for Figure 1F and S2E, the nDARs do not loose chromatin accessibility. However, for the SOX2 ChIP heatmaps, I wonder whether the potential residency time or dynamics for SOX2 binding is different for DAR versus nDAR after two hours dTAG-13 treatment? Or is this a consequence of slower depletion of some SOX2 proteins as was observed for SOX2 reduced expression (using dox/TET system) in Friman et al., Elife 2019? Please comment on the presence of SOX2 at 2h nDAR in the text. I suggest that you perform SOX2 ChIP at 6 hours dTAG-13 knockdown of SOX2 to show that the SOX2 enrichment at nDAR is lost at this later timepoint. This can be correlated with the chromatin opening at these SOX2 binding sites.
5. Related to the point above, it would be useful to compare differences in transcriptional activity between some DAR and nDARs in a reporter assay. The authors should clone a few representative nDAR and DAR sequences and perform SOX2 luciferase reporter assay in mESCs. Such reporter assays could also complement the comparison of SOX HMG and TAD domain activity in Figure S1 C-E.
6. For Figure S2F both DARs and nDARs show a relatively high enrichment on Strong Enhancer state but this has to my knowledge not been discussed in the manuscript. Can this be correlated with histone modifications described in Figure S2D?
7. Nascent transcriptional analysis shows both up and downregulated genes (Figure 3A) whereas ATACseq analysis shows most loss of chromatin accessibility vs gain of chromatin accessibility upon a time-course loss of SOX2 (Figure 2B/D).
In Figure 4, the focus has been on overlap of DEGs with downregulated genes. The role of transcription factors on repression of gene expression is often overlooked. Can the authors identify any overlap of DARs with upregulated genes and SOX2 binding sites? Can the authors identify the overlap here?
8. It is very interesting that Maresca et al., can distinguish sites for SOX2 pioneer function (defined bound with chromatin opening and gene activity) versus (bound without chromatin opening and low gene activity) using DEGs/nDEGs and nascent transcription within a two-hour window of SOX2 depletion (Figure 4D). I wonder whether there is any correlation of SOX2 binding with enrichment of OCT4 PWM and protein at these pioneering sites (? In other words, is SOX2 operating alone as a pioneer factor or (more often) as a heterodimer with OCT4 (as also addressed in Friman et al., Elife 2019)?
This observation may suggest that depletion of pioneer transcription factor SOX2 affect chromatin remodeling within two hours. However, according to Figure 1E, the washout and restoration of the chromatin opening after SOX2 depletion is slow, as there was only a limited restoration of chromatin opening after 2 hours. Complete restoration was observed after 24 hours. Is there a delay in reestablishment of SOX2 protein levels upon washout? A western should be performed to show this. Are there differential kinetics of active chromatin opening by SOX2 versus chromatin compaction upon loss of SOX2? The chromatin opening of SOX2 pioneering sites versus non-pioneering sites can be distinguished in ATACseq data in a washout time course to address this. How does this correlate with gene expression?
The authors could explore the overexpression S1E dataset with WT and TAD/HMG can be used to distinguish an effect on active chromatin opening by SOX2 at the pioneering sites versus non-pioneering binding sites.
9. The Klf2 locus mutant clones (Figure 5 and S4) are very interesting and show a marked Klf2 reduction in expression by qRT-PCR. A third replicate would be good to include as there are some discrepancies between some of the datapoints. I wonder how the KLF2 protein levels are affected? Can the authors show a western blot probing for KLF2 and SOX2 and loading control on

the WT vs Klf2 locus mutant clones?

10. The mutant clones C5, C10 and C13 shows differential loss of ATAC-seq signal at the down stream regulatory site but the chromatin accessibility upstream regulatory were not affected. Could the authors use available Hi-C data and CTCF ChIP to project the TAD structure and CTCF boundary regions of the Klf2 locus. What is the effect on chromatin looping of the downstream regulatory element with the promoter in the C13 clone? Will loss of SOX2 binding and chromatin accessibility of this regulatory region affect the interaction with the promoter? MCC in clone 13 could nicely address this question and shed some light on what impact pioneer transcription factors can have on higher order chromatin looping.

Minor concerns:

11. The title would benefit from the addition of "in embryonic stem cells" after "binding sites".
12. The introduction lacks references to methodology and literature. For example, electrophoretic mobility shift assay (EMSA) is a methodology extensively used to define protein (TF) - DNA interactions and should be included. For ChIP, a method originally described by Varshavsky and colleagues (Solomon et al., Cell 1988) and Crane-Robinson and colleagues (Hebbes et al., EMBOJ 1988), for whole genome read outs references given here are to pre-sequencing era. ChIP-seq was originally described in Barski et al., Cell 2007 (histones), and for DNA binding factors e.g., in Johnson et al., Science 2007 (NRSF) and Robertson et al., NatMet 2007 (Stat1).
13. In the introduction, reference to acute protein depletion method review is misspelled (Verma et al., 2020).
14. In results, line 4, please define which mESCs used (E14).
15. Add reference to the ATACseq method (Buenostro et al., Current protocols in molecular biology 2015) when first described in results.
16. Define "AUC" in the results.
17. Indicate molecular weight marker size on westerns (Figure 1B). What is the lower molecular band in SOX2 western? (It is also depleted upon addition of dTag). The figure legend describes western for NANOG; however, this is not shown?
18. For H3K64ac, a reference to Pradeepa et al., Nat Gen 2016 would be useful to include as it has been shown to be a histone mark enriched at active promoters and different types of active enhancers.
19. Add a scale bar (base pair) to better describe the size of the mutations in Figure S4B. Could the site for SOX2 binding be indicated? This is also to better understand that clone 13 has the complete binding site mutated compared to the other clones.
20. In Figure S4C legends the authors should describe number of replicates and error bars.
21. Genes when described should be written in italic. This applies to Figure 1D, 3C, 5A, S1, all S5.
22. Figure 4B is not described in the main text.
23. Please refer to Figure 5A when the MCC experiment is described in results.
24. Please revise this sentence in the results: "We could detect hardly any change in accessibility of the upstream elements in the clones in which the downstream elements were disrupted."
25. Figure S4D is not appropriately referred to in the manuscript.
26. What is the impact of acute knockdown of OCT4 and NANOG on chromatin accessibility in the Klf2 locus?
27. In the methods, was there any media change after addition of dTAG-13 before collection? Please clarify. The sentence of "DMSO was added at the same time as the latest dTAG-13 treatment" is confusing.
28. ChIP-seq methods needs some textual revisions. Also indicate the sonication time used and what type of beads used should be described.
29. Oligonucleotide table has empty rows, please revise.
30. List public datasets used in this study in a supplemental table.

We would like to thank the reviewers for their careful examination of our work. It was good to read that the reviewers we have a “powerful system” which provides an “interesting” and “valuable piece of work”.

Based on the reviewer comments we have improved the manuscript in a number of major ways:
1. We have performed ChIP for SOX2 and OCT4 using double crosslinking conditions (DSG-Formaldehyde). This has dramatically improved the quality of our ChIPseq tracks. The new ChIPseq data is now used throughout the manuscript.
2. We have added a more in-depth comparison of chromatin accessibility following SOX2 and OCT4 depletion. From this we learn that many sites depend on the activity of both SOX2 and OCT4, but that there is also a large contingent of sites that are lost exclusively when either SOX2 or OCT4 is depleted. We describe the differences in motif enrichment and TF binding to these sites.
3. In our original submission we disrupted an entire SOX2 binding site to determine the effect on expression. We have now also perform site-specific mutagenesis using CRISPR-Cas9 to disrupt a single SOX2 binding site. We find that this mutation affects the expression of the target gene, but not to the extent of a full site disruption.

For detailed answers to specific reviewer questions we refer point-by-point response below.

Referee #2:

In the paper by Maresca et al the authors utilize a degradation tag system to examine how an acute depletion of SOX2 in mouse ESCs, affects chromatin accessibility and gene expression. Two classes of SOX2 bound DNA-regions are identified; regions that become less accessible after SOX2 depletion and regions that remain open. SOX2 depletion results in a similar set of genes that are down- and upregulated. Finally, by comparing SOX2 binding to chromatin accessibility and gene transcription, the authors conclude that genomic regions that are dependent on SOX2 for their accessibility are more significant for SOX2 mediated gene activation, compared to accessible chromatin regions that are independent of SOX2.

The ability to rapidly deplete SOX2 protein expression provides a powerful system to examine the role of SOX2 in the regulation of chromatin accessibility and gene expression. One interesting finding is that SOX2 binding combined with chromatin accessibility is a better predictor of transcriptional regulation than SOX2 ChIP-seq data alone. However, the function of SOX2 as a pioneer factor has been established in several previous publications. Therefore, my major concern regards the general novelty and interest of their findings.

Specific comments:
1) Papers demonstrating the role of SOX2 as a pioneer factor should be properly referenced and discussed.

We thank for the suggestion of adding the references describing SOX2 as pioneer factor. We have now included the following references in the manuscript.

2) Like SOX2 depletion, OCT4 depletion leads to a loss of open chromatin regions. The authors should examine SOX2 expression in cells depleted for OCT4.

This is a good point. We have performed western blots to measure the expression of SOX2 in cells depleted of OCT4 after 6h and 24h. Compared to the non-treated (NT) condition, we observe a decrease in SOX2 levels only after 24h of OCT4 depletion. Given these data and the time it takes before an effect on SOX2 levels is observed we believe that the effects on open chromatin occur independently. This is also reflected in the fact that we find sites that seem to depend on SOX2 independent of OCT4 and which is now described in Figure 2.
We have also compared how SOX2 depletion affects OCT4 levels. We observe that there are no changes in OCT4 levels after SOX2 depletion.

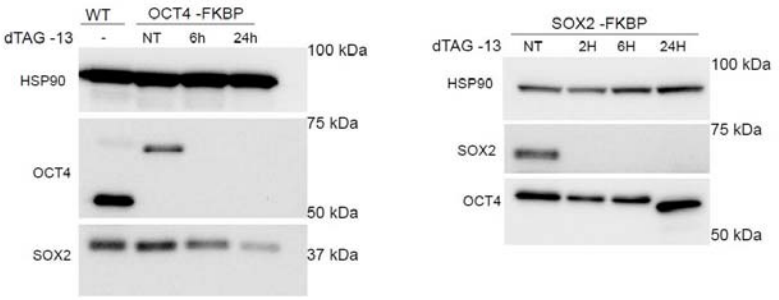

Reviewer figure 1: Left: western blot analysis showing levels of OCT4 and SOX2 in OCT4-FKBP line and WT, over a time course of dTAG-13 treatment. Right: Western blot analysis showing the levels of SOX2 and OCT4 in SOX2-FKBP cells at a time course of dTAG-13 treatment. HSP90 used as loading control.

3) As shown in Fig. 1, open chromatin regions are restored by washing-out the dTAG molecule after 2 hrs. The authors should examine how well SOX2 expression is restored following dTAG wash-out? The authors should determine if the same DNA-regions are "re-opened" after the dTAG wash-out as those closed after SOX2 depletion?

We thank the reviewer for this question, because this is an important point. We have thought about whether we can use the washout to study the dynamics of sites reopening. However, when we measured SOX2 protein levels at different timepoints following wash-off, we observe a gradual increase in SOX2 levels. SOX2 levels are comparable to non-depleted conditions only after 24h. Therefore, to assess the dynamics of chromatin site re-opening we need to factor in the dynamics of SOX2 re-constitution, which makes this very difficult to study. Alternatively, a two-component system in which SOX2 can be introduced rapidly in the nucleus with for instance an ERT domain following depletion can be used. However, we consider this beyond the scope of our manuscript at this point.
However, because we feel it is important to highlight the reversibility of the system, we restricted our analysis the 24 hour post washout time point. We provide the Western blot as Figure EV1 C and ATACseq in Figure 1F.

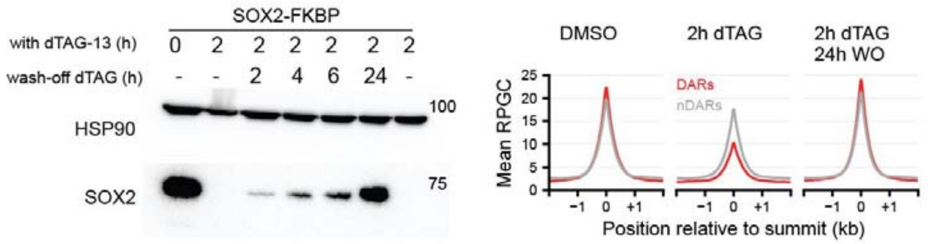

Left, Reviewer Figure 2: Left: Western blot showing SOX2 levels after 2h of Dtag-13 and its wash-out in a time course.
Right, Figure 1F: Average profile plots of ATACseq at SOX2 (n)DARs after dTAG treatment and 24h of wash out

4) In Fig. S1 the authors show that only full-length SOX2 can maintain open chromatin, and not the HMG or transactivation domain in isolation. The authors should discuss this finding in relation to previous publications showing that the HMG-domain of SOX2 has a pioneer function (e.g Dodonova et al., 2020).

We now provide the following text in the Discussion. “Previous CryoEM studies (Dodonova et al., 2020, Michael et al., 2020) of the DNA binding domains of OCT4 and SOX2 on nucleosomes showed that these proteins can bend the DNA, which may provide the initiating event that promotes the opening. However, our analysis in which we show that the DNA binding domain alone cannot rescue loss of chromatin accessibility following SOX2 depletion shows that additional activity is required for complete removal of the histone octamer. Our intersection with public ChIPseq data showed a very strong enrichment of SS18 at the sites that are lost following SOX2 depletion, suggesting that the mSWI/SNF complex could be instrumental in maintaining accessibility.”

5) In Fig. S2 the authors define SOX2 bound regions using ChIP-seq. To validate the quality of the ChIP the authors should present centrally enriched motifs within DNA regions suggested to be bound by SOX2. The authors should explain/discuss the strong enrichment of SOX2 motifs in unbound DARs compared to for instance bound OCRs (Fig. S2B).

This is a good point. We have repeated the ChIPseq of SOX2 using double cross-linking which resulted in a much larger set of binding sites. The amount of unbound DARs is now negligible (n=194). Therefore, we think it is no longer useful to discuss the unbound DARs. However, the suggestion by the reviewer to validate the quality of the ChIPseq samples is a good one. Below we show the alignment of the motif positions in the ChIPseq peaks relative to the summit of the peak. We have done this for both the SOX2 and the OCT4 ChIPseq data. We have also quantified the frequency of motifs per peak. These results show that all categories except the unbound OCRs have an above background frequency of motif frequency. We show these data in Figure EV2 B.

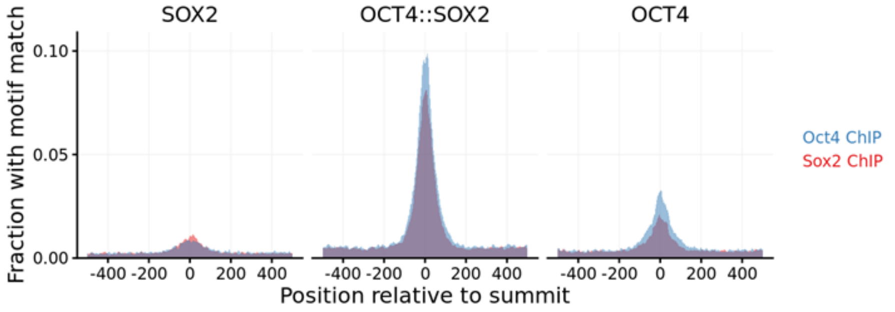

Reviewer figure 3: Coverage of motif position matches relative to summits of SOX2 and OCT4 ChIPseq peaks for the SOX2, OCT4::SOX2 and OCT4 motifs.

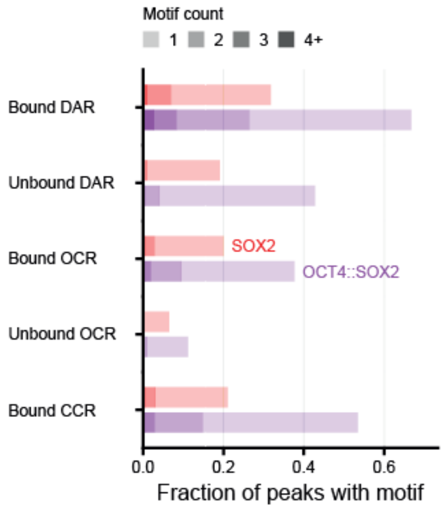

Figure EV2 B: Fraction of peaks containing 1 or more Oct4::Sox2 or Sox2 DNA binding motifs, stratified by whether they are accessible peaks as measured by ATACseq, SOX2 binding sites as measured by ChIPseq, differentially accessible after 2 hours of SOX2 depletion or combinations thereof.

6) In Fig. 2 the authors show that the chromatin accessibility is decreased more rapidly upon SOX2 depletion in DNA regions enriched for SOX motifs. Are regions in which the accessibility is decreased more slowly bound by SOX2, or is the decreased accessibility a secondary effect of SOX2 depletion? Is the expression of transcription factors proposed to bind slowly changing chromatin regions, e.g. KLF4, altered by SOX2 depletion?

This is a very interesting question. We have now performed SOX2 ChIPseq at 2 hours post depletion (hpd) and at 6 hpd. We do not find quantitative differences in the residual binding following depletion at 2 or 6 hours, therefore we consider it unlikely that this is the consequence of differences in the SOX2 binding levels. This is an interesting discussion, because one needs to define what constitutes a secondary effect.
One possible secondary mechanism is through the downregulation of a SOX2 target gene, such as *Klf4*, *Zic2* or *Zic3* (Reviewer Figure 4, below). Although *Klf4* and *Klf2* are decreased in expression, we consider it unlikely that this is of any consequence for the “slowly” changing sites, because we estimate the changes in the “slow sites” to occur within the sub hour time frame. It is unlikely that it is enough to change the mRNA levels and subsequently trickle down to the protein levels. To confirm this, we wanted to determine the protein levels of KLF2 and KLF4 on Western. Unfortunately, the KLF4 antibody we have does not detect KLF4 on Western. We also ordered a KLF2 antibody, but it did not arrive before the deadline of the resubmission.
An alternative scenario is that sites exist in the genome where SOX2 and, for instance, KLF4 bind together. This is what is suggested by our analysis of the dynamics of accessibility change following SOX2 depletion. Perhaps loss of SOX2 does not immediately lead to a loss of accessibility, because KLF4 is also bound there. We speculate that after loss of accessibility, KLF4 on its own cannot (re)create an open chromatin site. We will discuss this possibility in the Discussion. However, we have no data to mechanistically strengthen this hypothesis.

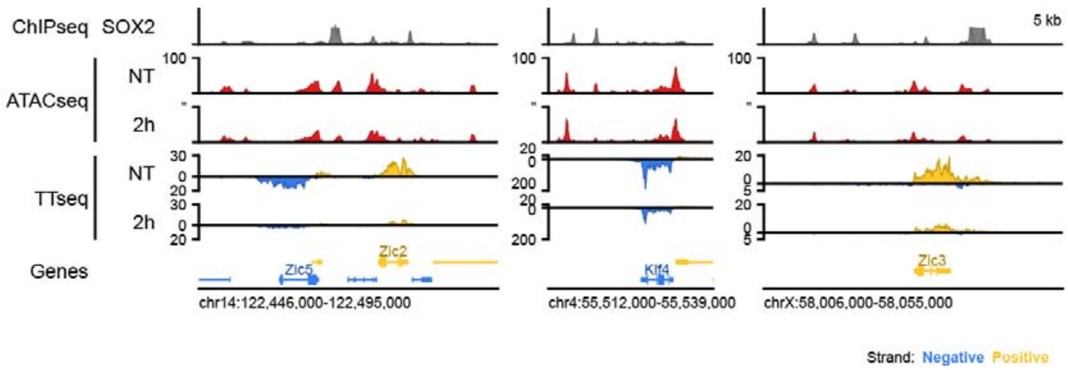

Reviewer figure 4: Klf4 and Zic2/3/5 are downregulated upon SOX2 depletion. Genomic tracks showing coverage profiles of SOX2 ChIPseq in NT condition and ATACseq and TTseq in NT and 2H depletion conditions, at the loci of *Zic2*/*Zic5*, *Klf4* and *Zic3*. Y-axes indicate RPGC.

Referee #3:

This is a very interesting study using acute protein degradation and multiple epigenomic assays to investigate the mechanisms that decide functionality of Sox2 binding sites in mouse embryonic stem cells. The authors found that Sox2 affects chromatin accessibility at a subset of its binding sites, which decides their gene regulation function. The manuscript is well written and the figures are of high quality. That being said, I have a few concerns/comments for the manuscript, particularly for the experimental validation part.

We thank the reviewer for their positive evaluation of our work. Below we address the concerns raised.

1. Figure 1F. It seems that DAR has stronger SOX2 binding than nDAR, which should be quantified. Does SOX2 binding intensity/frequency decides the pioneer activity and functionality, especially for the distal enhancer sites?

This is an good point. We have extensively tried to determine what chromatin features determine pioneer activity. We have redone the ChIPseq experiments and believe we now have more reliable set of SOX2 bound sites. There is definitely a correlation between the level of SOX2 binding and pioneer activity. Below we have ranked the SOX2 binding site based on the signal value determined from MACS2 peak calling in the untreated condition. What we find is that among the most strongly SOX2 bound sites DNA accessibility depends on SOX2 in ~75% of the cases. At lower SOX2 binding levels this percentage drops to below 10%. However, the binding level does not constitute a clear predictive feature for pioneer activity for an individual site, which is what we aimed for. We provide the figure showing the correlation of binding level with pioneer activity as Figure EV2 C.

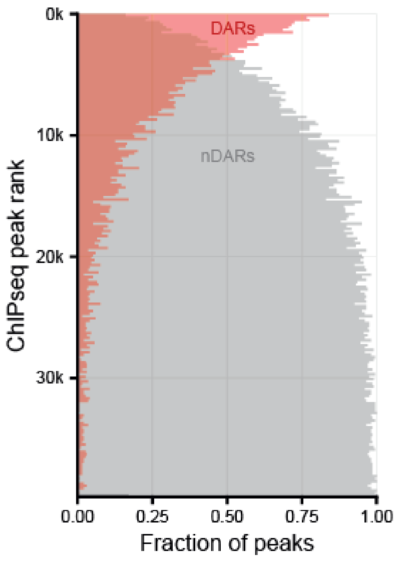

Figure EV2 C: Vertical histogram of SOX2 ChIPseq peaks ranked by signal intensity. The fraction overlapping with DARs and nDARs, regardless of OCR status, is displayed in 200 peak bins.

2. It would be interesting to compare the DAR sites regulated by Sox2 and Oct4. Do they overlap with each other? And what are the motifs enriched at the unique and shared DAR sites of Sox2 and Oct4?

We thank the reviewer for this question. We have compared the DARs from the SOX2-FKBP line and the OCT4-FKBP line following depletion. We find that 3168 sites are lost in both SOX2 and OCT4, showing that both OCT4 and SOX2 are required there. In addition, we find 3497 and 3205 sites that are specifically lost following SOX2 and OCT4, respectively. The SOX2 specific DARs are enriched for SOX-family motifs, but not POU-family motifs. The OCT4 specific DAR, on the other hand, are enriched for POU-family motifs, but not SOX-family motifs. The results from this analysis can be found in Figure 2. In the text we describe these results more extensively under the heading "SOX2 and OCT4 act both in partnership and independently to maintain accessibility".

3. For gene expression results, while SOX2 mainly activates its sites, the gene expression responses are seem in both directions (up and down) based on Figure 3A. It would be interesting to explore/discuss the mechanisms of how genes are upregulated in response to Sox2 degradation. Also, I would recommend compare the genes

expression changes in response to acute Sox2 degradation to those previously reported from traditional Sox2 knockdown assays in mESC and show how different they could be. In addition, it would be interesting to present pathways that these acute responsive genes are enriched in.

We have extensively scrutinized the upregulated genes, but have not so far been able to identify a unifying mechanism. Here we describe some of our observations.

1. Upregulation associated with the downregulation of an anti-sense non-coding transcript.
Below we show two examples of two genes that are upregulated following SOX2 depletion (*Top1* and *Suv39h1*). The TTseq shows overlap with a non-coding transcript that is transcribed from the opposite strand. These anti-sense transcription unit are associated with a open chromatin region that is lost upon SOX2 depletion. Although, this is an intriguing mode of regulation it is relatively rare. We provide the figure for Suv39h1 as Figure 4D in the paper. We also provide two examples of upregulated genes in Figure EV3 A-B.

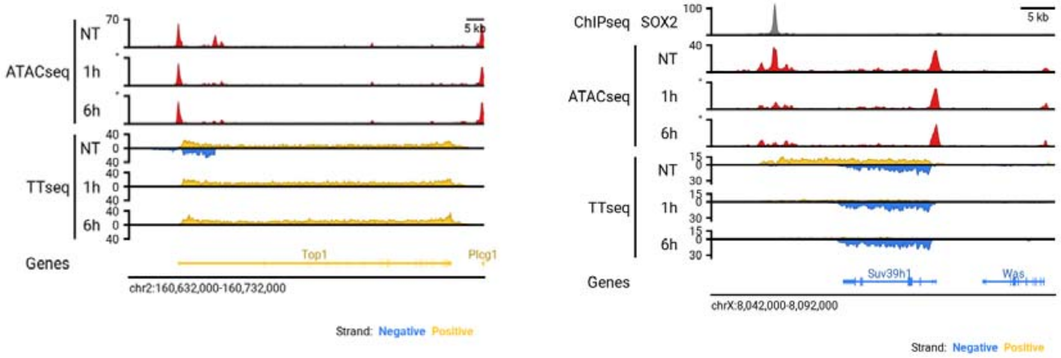

Left Figure EV3 A: example of *Top1* gene upregulation following SOX2 depletion. Red ATAC profile, Yellow TTseq tracks at the gene. Right Figure 4D: The same as in the left figure but for *Suv39h1* locus. In grey, SOX2 ChIPseq track.

2. Enrichment of nuclear receptor motifs in peaks flanking upregulated genes.
We selected the down- and upregulated genes at 2 hpd and selected the ATACseq peaks that were withing 20kb of the TSS of the gene (regardless of whether they changed accessibility). Next, we performed motif analysis on these peaks. As expected for the downregulated genes we find the compound OCT4::SOX2 motif significantly enriched, indicating that we can find potential regulators. When we repeat the same analysis for the upregulated genes we find enrichment of nuclear receptor motifs. Note that the indicated motifs are variations of the AGGTCA motif found in a.o. ESRRB. Why SOX2 depletion would mechanistically lead to upregulation specifically of these genes is unclear. The results from this analysis are now added in Figure EV3 C. We discuss these data as follows:

"Because there is a general lack of DARs that show an increase in accessibility, we perform motif analysis at all OCRs in the 40kb region centered at the TSS of downregulated genes and find a mild enrichment for OCT4::SOX2 and SOX family motif (Figure EV3 C). For upregulated genes, a similarly mild enrichment for nuclear receptor family motifs (e.g. ESRRB) is observed, but not SOX2. This indicates that upregulation cannot be explained a direct role of SOX2 as repressor."

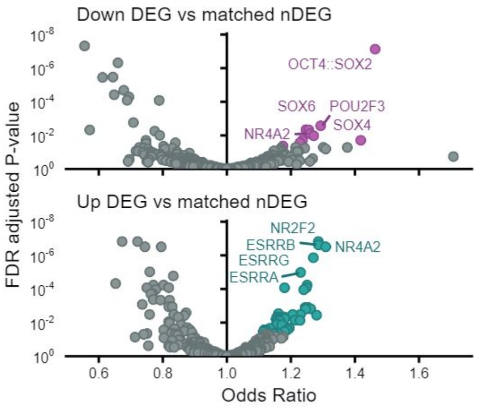

Figure EV3 C: Volcano plot showing the motifs found in OCRs in a 40kb window centered on the TSSs of downregulated DEGs and upregulated DEGs.

3. Transient upregulation following dTAG treatment.
Upon close inspection of the down- and upregulated genes we find that a much larger percentage of upregulated genes (28%) is transiently upregulated compared to the set of downregulated genes (11%).

We have performed overrepresentation analysis using the Reactome database. We find that across all timepoints there are 9 unique categories enriched among the differentially transcribed genes. Intriguingly, all 9 categories are found for the upregulated set of genes. This indicates that among the downregulated genes we cannot yet identify specific categories with for instance pluripotency. We provide a table with the significantly enriched categories as a Figure EV3 D.

Pathway	Foreground Ratio	Background Ratio	P-value FDR adjusted
**1H Up DEGs**			
Cholesterlos biosynthesis	20/320	25/5704	$1.4 \cdot 10^{-18}$
Metabolism of steroids	23/320	77/5704	$3.7 \cdot 10^{-9}$
Metabolism of lipids	49/320	392/5704	$1.0 \cdot 10^{-5}$
Hemostasis	39/320	321/5704	$4.3 \cdot 10^{-4}$
Cardiac conduction	12/320	57/5704	$7.2 \cdot 10^{-3}$
**2H Up DEGs**			
Cholesterol biosynthesis	20/602	25/5704	$5.7 \cdot 10^{-13}$
Metabolism of steroids	24/602	77/5704	$2.3 \cdot 10^{-4}$
Cardiac conduction	16/602	57/5704	$4.8 \cdot 10^{-2}$
**6H Up DEGs**			
Ion homeostasis	15/665	36/5704	$2.7 \cdot 10^{-3}$
RHO GTPase cycle	63/665	319/5704	$2.7 \cdot 10^{-3}$
Cardiac conduction	19/665	57/5704	$2.7 \cdot 10^{-3}$
RAC1 GTPase cycle	31/665	121/5704	$2.7 \cdot 10^{-3}$
Muscle contraction	23/665	86/5704	$1.4 \cdot 10^{-4}$

Figure EV3D: Table showing pathway overrepresentation analysis results on TT_{chem}-seq DEGs following SOX2 depletion. Table shows all significant hits at the FDR adjusted p-value threshold lower than 0.05, stratified by timepoint and direction. For 0.5h and downregulated DEGs, no significant pathway enrichments were found.

4. I am somehow confused of Figure 4A. If Sox2 DAR sites are mostly distal enhancers (based on ChromHMM results), how come they are enriched at TSS regions of DEG? Was this due to the 10kb bins that the authors used in the figure? Would a higher resolution plot in a smaller window centered at TSS work better?

There are two factors at play for this seeming contradiction. The first is that in Fig 5A (previously Fig 4A), we focused on the 618 TSSs with known transcripts that are downregulated out of the total 26.859 transcribed units we have considered. Globally, this means that TSSs are not enriched for DARs, as exemplified by the control nDEGs as representative of all other TSSs in Fig 5A. The second is that nDARs do have a mild enrichment near TSSs, whether DEG or nDEG, whereas this is absent for the DARs for nDEGs TSSs, which appears mostly flat at the chosen distance range of +/-250kb. If we make the bins 10 times smaller, we still observe the enrichment near the DEG TSSs, albeit with more noise.

5. The experimental validation needs more work in my opinion. First, for the klf2 locus, there are two additional SOX2 DAR sites upstream of Klf2, which should be included in the validation (the authors already discussed some hypothesis for them, which need to be tested). Second, deletion of the entire DAR site may affect many other bound transcription factors. How would we know the effects come from Sox2? Could the authors just cut (by one guide) or delete the SOX motif in the site? or there are multiple SOX motifs within it, making it difficult to choose where to cut? Finally, I think including additional example(s) for validation, especially for genes that have nDAR Sox2 sites, would strengthen the conclusion a lot.

We fully agree that validating the other regulatory elements in the *Klf2* locus would be very informative, specifically with regards to the cooperativity and superadditivity of the elements for *Klf2* expression. To this end we have previously tried to generate a deletion clone for the proximal upstream element. However, following two rounds of CRISPR-Cas9 targeting we only obtained heterozygous clones.

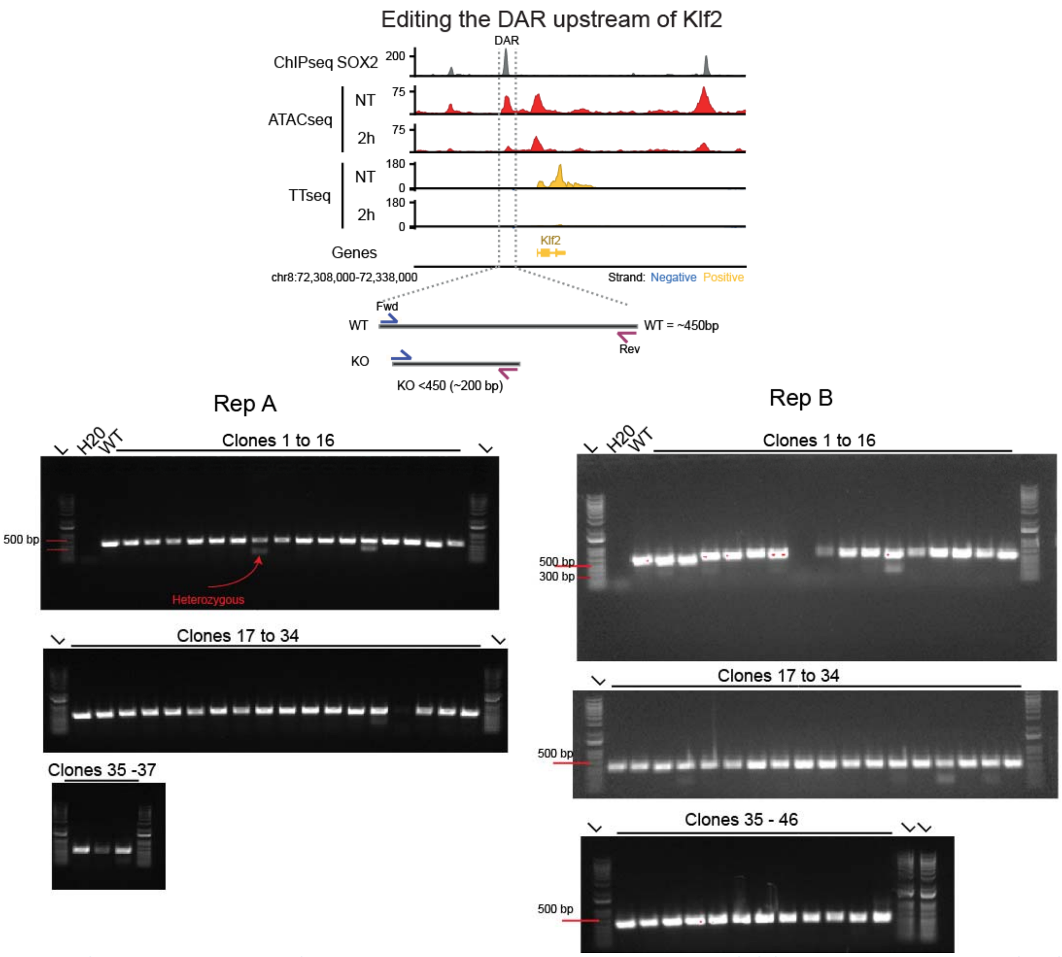

Reviewer figure 5: Example of the KLF2 locus during a time course of SOX2 depletion. Tracks for SOX2 ChIPseq. ATACseq changes, TTseq expression changes in the locus are shown. Upstream the Klf2 a DAR region targeted for depletion is highlighted. PCR primers were designed to validate genomic deletion at the DAR. Bottom: Gel electrophoresis of the PCR performed on clones that underwent editing. For genotyping primers spanning the depleted region were used. Unedited cells will show a band on gel corresponding to ~450 bp. Deletion in the region will generate an amplicon smaller than WT (<450). Only heterozygous or unedited clones were found for this deletion. L, ladder.

With regard to the regulatory element disruption, the reviewer makes an excellent point. To specifically target only the SOX2 binding site we have used CRISPR-Cas9 coupled to homology directed repair. We identified that SOX2 motif in the regulatory element and mutated the site. We found that on average *Klf2* was downregulated by 42% (Figure 6B). This is a less drastic loss of expression compared to the full disruption of the element, which indicates that there is additional signal in the sequence to drive expression. We have now added those results to Figure 6. We now describe these experiments in the Results section as follows: “We used CRISPR-Cas9 coupled with homology directed repair (HDR) to mutate the core SOX2 motif within the DAR region 15kb downstream of *Klf2*. The mutation is expected to impair SOX2 binding, while keeping the surrounding DNA sequence intact. We selected 5 clones homozygous for the motif mutant and measured *Klf2* expression levels by qPCR (Figure 6B, Figure EV5 A-B). We find that mutation in the SOX2 motif leads to a ~45% downregulation of the *Klf2* gene”

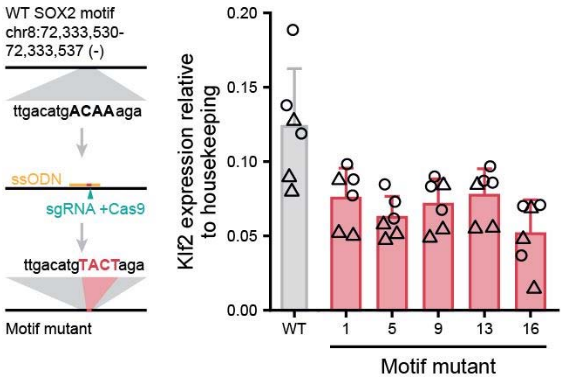

Figure 6B: Left; schematic of the approach for site directed mutagenesis of the SOX2 motif within the DAR in the Klf2 locus. Right, RT-qPCR of Klf2 expression measured in WT and SOX2 mutant clones. Error bars represent standard deviation of 6 replicates. Circle and triangle represent two set of primers used. Expression is normalized over the housekeeping *Rsp26* gene.

Some minor points:
- Figure S2E listed 5461 DAR and 5488 nDAR sites, which are different from the numbers shown in Figure 1F. What are the differences behind them?

We thank the reviewer for pointing out this discrepancy. We mistakenly took the wrong file for Figure 1F, now Figure 1E. In the revised version, we corrected this mistake and amended to selection procedure for the nDARs, so they are as abundant as the DARs. Moreover, the nDARs now have matched accessibility levels in NT condition and partially matched SOX2 levels in the new ChIPseq data, as these could not be perfectly matched due to high intensity SOX2 binding regions mostly being DARs (see also first figure in response to reviewer #3 point 1).

- It seems from Fig S2B that unbound DARs are also enriched with SOX2/OCT4:SOX2 as compared to unbound OCRs. Are these sites also enriched with Sox2 binding (based on ChIP-seq signal) but were not called as peaks?

The suggestion of the reviewer is correct. We have redone the ChIPseq using double crosslinking and higher sequencing depth yielding many more detectable peaks. Indeed, we detected SOX2 peaks at nearly all DARs with the improved ChIPseq data.

- Sox2 ChIP-seq tracks should be added to Figures 1D, 2A, 3C, S1A. S1A also needs Oct4 and Nanog tracks.

As per the reviewers suggestion we have added ChIP tracks to the relevant depletion dataset (i.e. SOX2 to SOX2 depletion, OCT4 to OCT4 depletion, etc.). This has been added to Figures 1D, 3A, 4C and Figure EV1 A. Please note that the ChIPseq data used for NANOG was taken from publicly available data from GEO accession GSM2123560.

Referee #4:

Maresca and colleagues describes the effect on chromatin accessibility and nascent transcription after acute SOX2 degradation in murine ES cells. The short time-course reveals distinct SOX2 regulomes divided into SOX2 pioneering binding sites where chromatin is open and SOX2 binding sites where chromatin accessibility is not affected. To further elucidate the effect of SOX2 pioneering activity on chromatin opening, the authors focused on two SOX2 bound regulatory sequences in the Klf2 locus that they had showed interacted with the Klf2 promoter. CRISPR edited mutations of the SOX2 binding site in the downstream regulatory region reduced chromatin accessibility and abrogated Klf2 expression.

In general, this is a valuable piece of work as it provides detailed characterisation of SOX2 and its pioneering activity in mES cells. The manuscript is well written, and the figures are nice and clear. The study addresses some of the limitations of the Friman et al., Elife 2019 study, where time for reduction of SOX2 was much longer (towards 40 hours). The acute SOX2 protein degradation observed here has improved the elucidation of the pioneering function of this TF. However, I have some concerns that I wish that the authors should address which are listed below.

We thank the reviewer for their positive comments on our manuscript. Below we address the comments from the reviewer.

Major comments:

1. As SOX2 protein levels have been shown to affect OCT4 levels (Strebinger et al., MolSystBiol 2019), a western representing the OCT4 protein during the time-course of SOX2-FKBP degradation should be shown (Figure 1B). If OCT4 protein levels are affected this should be discussed. SOX2 and OCT4 binds as heterodimers at closed chromatin (Soufi et al., Cell 2015 and Echigoya et al., SciRep 2020). It is therefore pertinent that the authors show and discuss what effect acute SOX2 depletion have on global OCT4 enrichment. A ChIP-seq of OCT4 should be conducted upon acute SOX2 kd time course incubation with dTag-13. With the acute knockdown of SOX2 and OCT4, can the authors re-examine the cooperational relationship between SOX2 and OCT4 on chromatin accessibility as has been addressed in Friman et al., Elife 2019?

We thank the reviewer for this suggestion. We performed western blots of SOX2 and OCT4 following SOX2 depletion and do not detect changes in global OCT4 protein levels (see Reviewer figure 6). We have now additionally performed ChIPseq for OCT4 in the SOX2-FKBP line, and for SOX2 and OCT4 in the OCT4-FKBP line. We observe that DARs, both specific and common ones, are co-occupied by both SOX2 and OCT4. After either depletion of SOX2 or OCT4, we generally find strongly reduced binding of the cooperator, suggesting that at most detected DARs, cooperative binding is the major mode of binding. A notable exception to this is that after OCT4 depletion, SOX2 remains largely bound at SOX2 specific DARs, indicating that OCT4 is present but not necessary for accessibility at these sites. These results are discussed as follows in the text:

“To determine the contribution of SOX2 and OCT4 binding to accessibility, we performed ChIPseq of OCT4 in the SOX2-FKBP line and of SOX2 in the OCT4-FKBP line (Fig 2D). We find that all DARs we identified, regardless of their specificity to SOX2 and OCT4 depletion, are bound by both SOX2 and OCT4 together. Upon depletion of the tagged TF, the ChIPseq signal of the tagged TF disappears. Generally, we also find that the signal of the partner TF at DARs is greatly reduced by, suggesting that OCT4 and SOX2 stabilize each other’s binding affinity to chromatin. The exception to the general case is that the SOX2 DARs after OCT4 depletion only mildly decreased in SOX2 binding intensity (27%), indicating that SOX2 is able to maintain accessibility at these sites independent of OCT4. Conversely, OCT4 binding intensity is reduced by 66% at OCT4 DARs after SOX2 depletion, hinting that accessibility is maintained here through alternative means. These results demonstrate that despite the co-occupation of SOX2 and OCT4 proteins at most DARs, accessibility changes upon pioneer TF depletion are not solely encoded by binding patterns in non-treated conditions.”

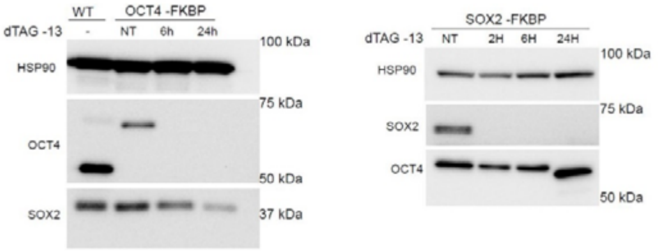

Reviewer figure 6: Left: western blot showing levels of OCT4 and SOX2 in OCT4-FKBP line and WT, over a time course of dTAG-13 treatment. Right: Western blot analysis showing the levels of SOX2 and OCT4 in SOX2-FKBP cells at a time course of dTAG-13 treatment. HSP90 used as loading control.

2. Furthermore, to follow up on the point above, for Figure 1E, washout and restoration of chromatin opening should be complemented with a western blot to show SOX2, OCT4 and loading control protein levels.

This is a good point we have added the Western blot for SOX2. Since OCT4 levels do not change following SOX2 depletion, we feel the Western of the OCT4 will probably not add a lot of information.

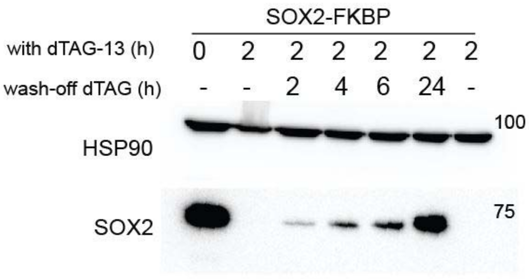

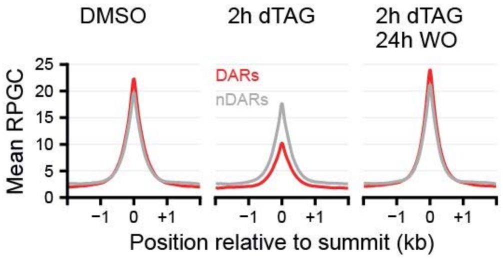

Left, Reviewer figure 7: Western blot showing SOX2 levels after 2h of dTAG-13 and its wash-out in a time course.
Right, Figure 1E: Average plot of ATACseq profile plotted over the SOX2 n(DARs) in SOX2 depletion in DMSO, 2h dTAG-13 SOX2 depletion and after 24h of dTAG-13 Washout (SOX2 protein restoration).

3. Cistrome scoring analysis: Figure S2D is not referred to in the text, please revise . It is interesting that OCT4 and NANOG are relatively high up on the list as "Enriched" and that these are also known to interact with SOX2. SALL4 has also been shown to interact with SOX2 and OCT4 (Tanimura et al., JBC 2013) and should be discussed. Can Maresca and colleagues identify other SOX2 interacting proteins among the "Enriched" versus "Depleted" factors?

We apologize for this omission. We now refer to Figure EV2 D under the paragraph entitled: “*Continuous pioneering activity of transcription factors is necessary for maintenance of accessible chromatin”.*

Specifically: “Among the top scoring factors we find OCT4 and NANOG, but also ZFP57 and the BAF (a.k.a. mSWI/SNF) complex member SS18 (King and Klose, 2017), which showed specific binding at DARs (Figure EV2 D).”

4. As you have described for Figure 1F and S2E, the nDARs do not loose chromatin accessibility. However, for the SOX2 ChIP heatmaps, I wonder whether the potential residency time or dynamics for SOX2 binding is different for DAR versus nDAR after two hours dTAG-13 treatment? Or is this a consequence of slower depletion of some SOX2 proteins as was observed for SOX2 reduced expression (using dox/TET system) in Friman et al., Elife 2019? Please comment on the presence of SOX2 at 2h nDAR in the text. I suggest that you perform SOX2 ChIP at 6 hours dTAG-13 knockdown of SOX2 to show that the SOX2 enrichment at nDAR is lost at this later timepoint. This can be correlated with the chromatin opening at these SOX2 binding sites.

We thank the reviewer for raising this point. We understand how the reviewer might come to the conclusion that these are perhaps slow sites or that there may be residual binding. To alleviate these concerns, we have performed two analyses. First, as suggested by the reviewer we have performed ChIPseq of SOX2 following 6 hours of SOX2 depletion. As can be seen from the figure below the level of SOX2 binding seems to be the same as for 2 hpd, suggesting that this is not due to residual SOX2 binding at 2 hpd. Second, we now also show the ATACseq data at 6 hpd, which also seems very similar to the 2hpd data. From these data we conclude that accessibility at the nDARs bound by SOX2 are not critically dependent on SOX2. We describe these results in the text as follows:

“To determine how DARs relate to SOX2 binding, we performed ChIPseq with an antibody to the HA-tag in the SOX2 fusion protein in absence and presence of the dTAG-13 degrader. To our surprise, a sizable fraction of SOX2 bound regions did not lose accessibility (non-DAR: nDAR) (Fig 1E, Figure EV2 A). We performed ChIPseq of HA-tagged SOX2 at 2 and 6 hpd to show that the nDARs are not a consequence of residual binding of SOX2 (Fig 1E)”.

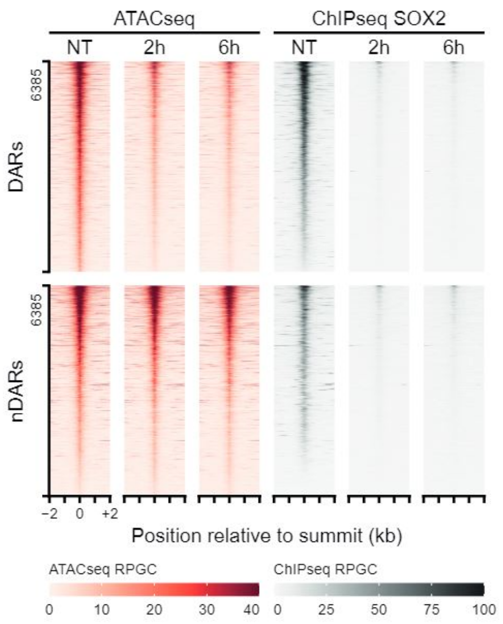

Figure 1E: Heatmap showing accessibility and SOX2 ChIPseq before and after dTAG treatment in SOX2-FKBP cell line at SOX2 peaks that are differentially accessible regions (DAR) or where no differentially accessible region is detected (nDAR) partially matched for SOX2 binding. RPGC: reads per genomic content.

5. Related to the point above, it would be useful to compare differences in transcriptional activity between some DAR and nDARs in a reporter assay. The authors should clone a few representative nDAR and DAR sequences and perform SOX2 luciferase reporter assay in mESCs. Such reporter assays could also complement the comparison of SOX HMG and TAD domain activity in Figure S1 C-E.

The reviewer raises an interesting point. However, we are somewhat worried by the anecdotal nature of choosing a set of DARs and nDARs for a targeted reporter assay. To counter this concern, we have used publicly available STARRseq data from Peng et al. (2020) (a massively parallel reporter assay or MPRA) to determine how DARs and nDARs behave in a reporter setting. The figure below (Reviewer figure 8) shows the result of our analyses. The DARs show a significantly higher propensity to drive expression compared to bound nDARs.

Although interesting, we feel that these results may act as a distraction for the message of our manuscript. It should be noted that it is unclear whether in plasmid-based reporter assays the regulatory sequences are found in a chromatinized state. Our manuscript is precisely about the role of chromatin and chromatin remodeling on gene expression in a native context, which we believe our assays can faithfully measure. Therefore, it would be our preference to leave these results out of the manuscript. However, if the reviewer feels these results are important for the manuscript we would be happy to include them.

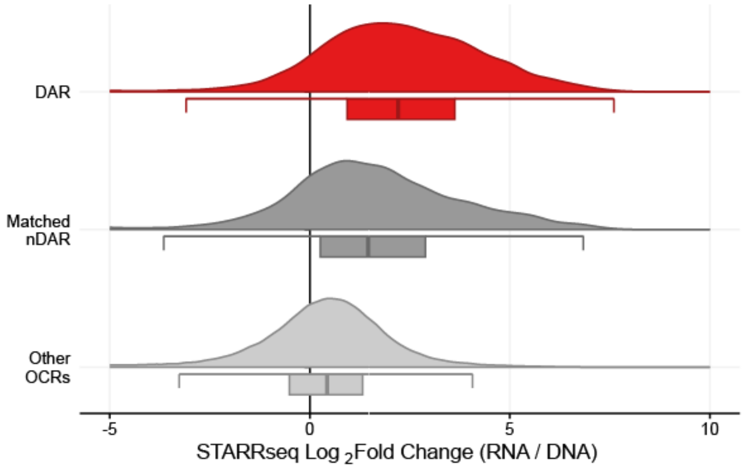

Reviewer figure 8. DARs tend to have higher MPRA enrichment over other OCRs. Quantification of STARRseq signal in 2i/LiF conditions at all OCRs, as measured by the $\log_2$ fold change of the RNA reads over the DNA reads. OCRs are subdivided for downregulated DARs, nDARs that are partially matched for SOX2 levels, and all other OCRs, shown as a kernel density estimate and boxplot..

6. For Figure S2F both DARs and nDARs show a relatively high enrichment on Strong Enhancer state but this has to my knowledge not been discussed in the manuscript. Can this be correlated with histone modifications described in Figure S2D?

We thank the reviewer for raising the issue. We have collected better ChIPseq data for SOX2 and consequently re-categorised the matched nDARs that we use in this comparison, based on accessibility in the NT condition and partially SOX2 levels. The relative enrichment of the strong enhancer state is now comparable between DARs and their matched equivalents. We show this in the updated Figure EV2 F.

7. Nascent transcriptional analysis shows both up and downregulated genes (Figure 3A) whereas ATACseq analysis shows most loss of chromatin accessibility vs gain of chromatin accessibility upon a time-course loss of SOX2 (Figure 2B/D).
In Figure 4, the focus has been on overlap of DEGs with downregulated genes. The role of transcription factors on repression of gene expression is often overlooked. Can the authors identify any overlap of DARs with upregulated genes and SOX2 binding sites? Can the authors identify the overlap here?

This is an interesting point. For completeness, we've added the analysis of Fig 5A (previously Fig 4A) for different directions of DARs and DEGs as Fig EV4 A. We have checked at 2hpd how many DARs are found within 40kb of an upregulated gene. We find 79 and for genes with a 2-fold upregulation, this number shrinks to 10. Below we have shown an example of the *Fgfbp1* gene, in which we find a DAR close to the TSS (Reviewer figure 9). Note that this is the best example that we could find among the list of 10 upregulated transcripts. Given the low number of sites that we find and the fact that we find a depletion, see figure below, rather than an enrichment of DARs around the upregulated genes, we are reluctant to include these results into the final manuscript.

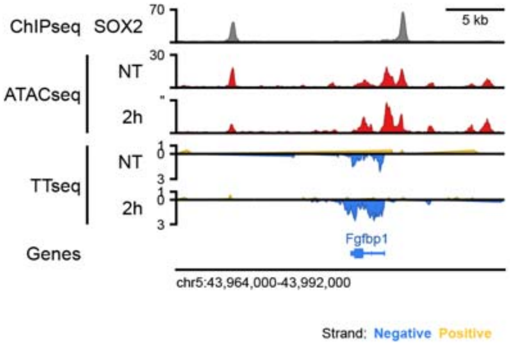

Reviewer figure 9: Genomic tracks showing the *Fgfbp1* locus showing SOX2 ChIPseq in NT condition, ATACseq and TTseq at NT and 2 hours post SOX2 depletion conditions.

An interesting example of DARs associated with upregulated comes from the genes *Top1* and *Suv39h1*. For these genes there is a DAR, but it is actually associated with an upregulated non-coding anti-sense transcript (see figures below). This could point to a mechanism in which transcription of the anti-sense transcript inhibits expression of the protein coding gene. Using our high-resolution time-series we can distinguish the order of events. This could indicate a mechanism for repression by SOX2, albeit through an indirect mechanism. We describe these examples in the Results section as follows:
"An example of an upregulated gene is *Suv39h1*, which is associated with the downregulation of an anti-sense non-coding transcript coming from the opposite strand. The TSS of this antisense transcript is associated with an open chromatin region that is lost upon SOX2 depletion (Fig 4D). A similar trend is observed for *Top1* (Figure

EV3A), suggesting that SOX2 regulating an anti-sense transcripts may be a mechanism for repressing protein coding genes.”

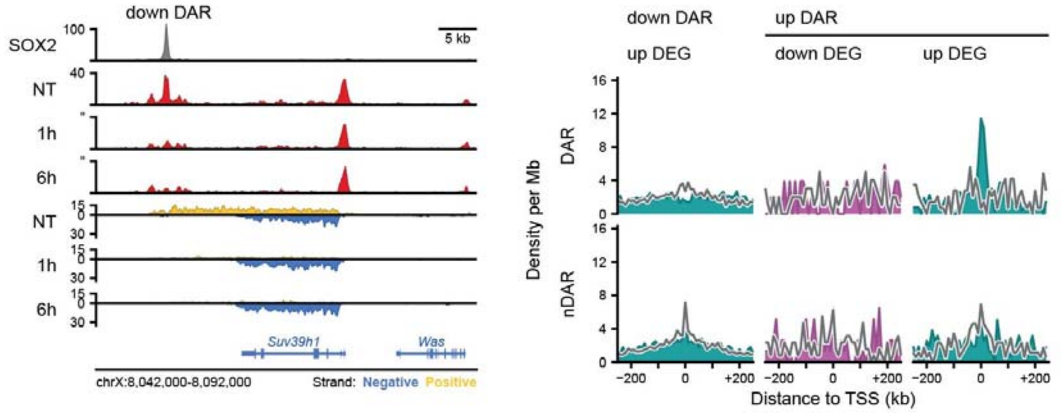

Left, Figure 4D: Genomic tracks showing SOX2 ChIPseq (top), ATACseq data (middle) and nascent transcription measured with TT_{chem}seq (bottom) at the *Suv39h1* locus in untreated (NT) 0.5 and 1h dTAG-13 treated SOX2-FKBP cells.

Right, Figure EV4 A: Density of peaks in 10kb bins nearby transcription start sites (TSS) of upregulated (light blue) and downregulated (purple) genes and their expression-matched stable control (grey) genes. Panels indicate the set of peaks that were aligned to the TSS: down DARs (n = 6,385) and up DARs (n = 408). Bottom row indicates the density for an equal number of stable nDARs.

8. It is very interesting that Maresca et al., can distinguish sites for SOX2 pioneer function (defined bound with chromatin opening and gene activity) versus (bound without chromatin opening and low gene activity) using DEGs/nDEGs and nascent transcription within a two-hour window of SOX2 depletion (Figure 4D). I wonder whether there is any correlation of SOX2 binding with enrichment of OCT4 PWM and protein at these pioneering sites (? In other words, is SOX2 operating alone as a pioneer factor or (more often) as a heterodimer with OCT4 (as also addressed in Friman et al., Elife 2019)?

This is indeed an important point. We have now included a more systematic analysis of DARs that were identified following SOX2 and OCT4 depletion. We find that the number of sites that are lost uniquely following SOX2 or OCT4 depletion and the sites that are dependent both on OCT4 and SOX2 are split roughly equally (>3000 sites each). We find the strongest enrichment for the compound motif OCT4::SOX2 for the sites that are dependent on both OCT4 and SOX2. For the sites that are not dependent on OCT4, but are lost only after SOX2 depletion we find that these are not enriched for the POU-family motifs (i.e. OCT). Conversely, for the sites that are lost after OCT4 depletion, but not SOX2 depletion we find no enrichment of SOX-family motifs. Those results are now described under the paragraph entitled: ‘’*SOX2 and OCT4 act both in partnership and independently to maintain accessibility‘’* and related Figure 2 and Appendix Figure 1.

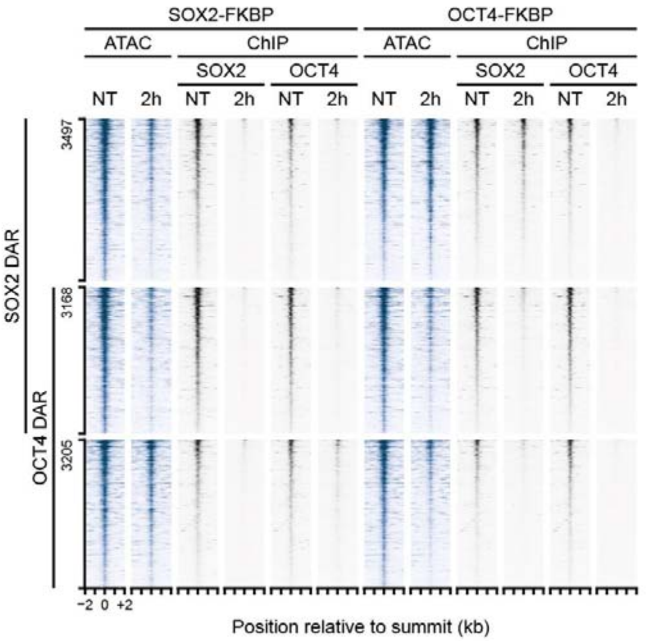

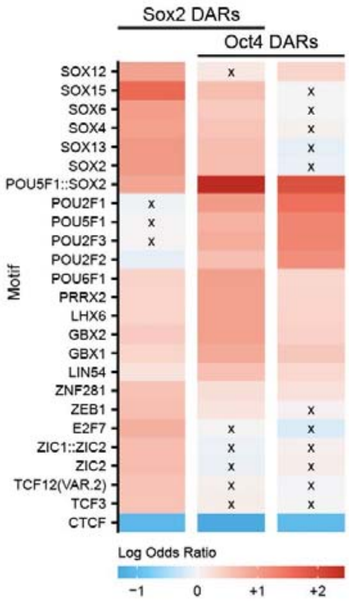

Left, Figure 2D: Tornado plots showing ATACseq (blue) after SOX2 and OCT4 depletion. Regions are divided by SOX2, Common and OCT4 DARs. In grey, ChIPseq signal of SOX2 and OCT4 in SOX2 depletion and OCT4 depletion. RPGC, reads per genomic content.
Right, Figure 2C: Heatmap showing the top 25 motifs at SOX2, common and OCT4 DARs. Color-bar indicates odds of finding the motif in the DAR set relative to all other OCRs. Crosses denote non-significant odds.

This observation may suggest that depletion of pioneer transcription factor SOX2 affect chromatin remodeling within two hours. However, according to Figure 1E, the washout and restoration of the chromatin opening after SOX2 depletion is slow, as there was only a limited restoration of chromatin opening after 2 hours. Complete restoration was observed after 24 hours. Is there a delay in reestablishment of SOX2 protein levels upon washout? A western should be performed to show this. Are there differential kinetics of active chromatin opening by SOX2 versus chromatin compaction upon loss of SOX2? The chromatin opening of SOX2 pioneering sites versus non-pioneering sites can be distinguished in ATACseq data in a washout time course to address this. How does this correlate with gene expression?

We thank the reviewer for this question. We have now performed a Western blot following dTAG-13 washout. As suggested by the reviewer restoration of the SOX2 expression to pre-depletion levels is difficult and relatively slow (compared to the rate of depletion). Because the level of SOX2 following washout is still very low, our previous results are likely a consequence of incomplete SOX2 restoration. Because of this we have decided to leave out the 2 hour washout dataset and instead decided to focus on the 24hour washout dataset. We believe that washout unfortunately cannot be used to determine dynamics of gains in chromatin accessibility (see also answer to reviewer 1). However, we believe it is important to show that washout can lead to reversibility of the chromatin accessibility phenotype.

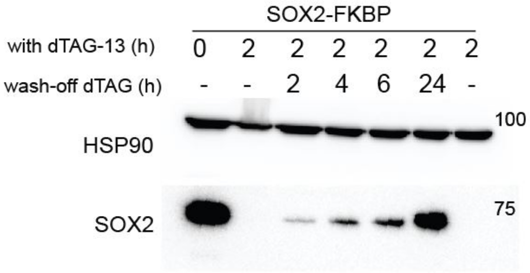

Reviewer figure 10: Western blot analysis showing SOX2 levels after 2h of dTAG-13 and its wash-out in a time course.

The authors could explore the overexpression S1E dataset with WT and TAD/HMG can be used to distinguish an effect on active chrom]atin opening by SOX2 at the pioneering sites versus non-pioneering binding sites.

We thank the reviewer for this suggestion. We'd like to highlight that the nDARs chosen in figure 1F and Figure EV1 E are also enriched for SOX2 binding sites because the levels of SOX2 binding are partially matched and hence qualify as non-pioneering binding sites. We do not find any specific change at these non-pioneering sites.

9. The Klf2 locus mutant clones (Figure 5 and S4) are very interesting and show a marked Klf2 reduction in expression by qRT-PCR. A third replicate would be good to include as there are some discrepancies between some of the datapoints. I wonder how the KLF2 protein levels are affected? Can the authors show a western blot probing for KLF2 and SOX2 and loading control on the WT vs Klf2 locus mutant clones?

We have now added a third biological replicate for qRT-PCR and measured *Klf2* expression. This can be found in Figure 6C and Figure EV5 E. Probing KLF2 protein levels indeed is an interesting question. To this end we ordered a KLF2 antibody. Unfortunately, at the time of writing, this antibody has not yet come in, precluding us from performing Western blot analysis for KLF2.

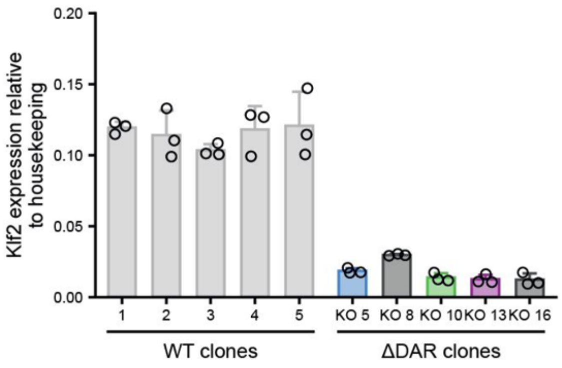

Figure 6C: Expression of *Klf2* gene measured with RT-qPCR in 5 biological clones of WT and 5 clones of DAR KO. Expression is normalized over housekeeping gene *Rsp26* (n=3).

10. The mutant clones C5, C10 and C13 shows differential loss of ATAC-seq signal at the down-stream regulatory site but the chromatin accessibility upstream regulatory were not affected. Could the authors use available Hi-C data and CTCF ChIP to project the TAD structure and CTCF boundary regions of the Klf2 locus. What is the effect on chromatin looping of the downstream regulatory element with the promoter in the C13 clone? Will loss of SOX2 binding and chromatin accessibility of this regulatory region affect the interaction with the promoter? MCC in clone 13 could nicely address this question and shed some light on what impact pioneer transcription factors can have on higher order chromatin looping.

This is an interesting question. While we can definitely add the TAD structure around the Klf2 locus, we are a bit hesitant to do so. We feel that putting more focus on the 3D would distract too much from the main message of paper, which is not necessarily about the 3D organization of the locus around *Klf2*.

Minor concerns:
11. The title would benefit from the addition of "in embryonic stem cells" after "binding sites".

We thank the reviewer for their thoughts on the completeness of the title, though we prefer the brevity of the current title.

12. The introduction lacks references to methodology and literature. For example, electrophoretic mobility shift assay (EMSA) is a methodology extensively used to define protein (TF) - DNA interactions and should be included. For ChIP, a method originally described by Varshavsky and colleagues (Solomon et al., Cell 1988) and Crane-Robinson and colleagues (Hebbes et al., EMBOJ 1988), for whole genome read outs references given here are to pre-sequencing era. ChIP-seq was originally described in Barski et al., Cell 2007 (histones), and for DNA binding factors e.g., in Johnson et al., Science 2007 (NRSF) and Robertson et al., NatMet 2007 (Stat1).

We thank the reviewer for providing these useful suggestions for citations. We have added them to the Introduction.

13. In the introduction, reference to acute protein depletion method review is misspelled (Verma et al., 2020).

We that the reviewer for spotting this mistake, we have adapted the reference.

14. In results, line 4, please define which mESCs used (E14).

Good point, we use E14Tg2A (129/Ola). This has been added to the main text.

15. Add reference to the ATACseq method (Buenostro et al., Current protocols in molecular biology 2015) when first described in results.

This has been added.

16. Define "AUC" in the results.

This has been done.

17. Indicate molecular weight marker size on westerns (Figure 1B). What is the lower molecular band in SOX2 western? (It is also depleted upon addition of dTag). The figure legend describes western for NANOG; however, this is not shown?

We have added the molecular weight marker to western blot. We apologize for the mentioning of NANOG in the description. This present in an earlier version, but was later removed (See Liu et al, 2021 for the generation of the NANOG line).

18. For H3K64ac, a reference to Pradeepa et al., Nat Gen 2016 would be useful to include as it has been shown to be a histone mark enriched at active promoters and different types of active enhancers.

Good point we have added the reference.

19. Add a scale bar (base pair) to better describe the size of the mutations in Figure S4B. Could the site for SOX2 binding be indicated? This is also to better understand that clone 13 has the complete binding site mutated compared to the other clones.

We have now included a scale bar in the figure. This figure is now in Figure S6F.

20. In Figure S4C legends the authors should describe number of replicates and error bars.

This has been added.

21. Genes when described should be written in italic. This applies to Figure 1D, 3C, 5A, S1, all S5.

This has been added.

22. Figure 4B is not described in the main text.
We apologize for the mistake. We have now added the description of the figure in the text. This is now Figure 5B.

23. Please refer to Figure 5A when the MCC experiment is described in the results.
We have now added the reference to the figure. Note that now this refers to Figure 6A.

24. Please revise this sentence in the results: "We could detect hardly any change in accessibility of the upstream elements in the clones in which the downstream elements were disrupted."

We thank for this point. We have now rephrased the sentence to “To test this, we performed ATACseq in three of the clones (Fig 6D) and could detect hardly any changes in accessibility of the upstream elements.”

25. Figure S4D is not appropriately referred to in the manuscript.
We have now referenced it in the paper.

26. What is the impact of acute knockdown of OCT4 and NANOG on chromatin accessibility in the Klf2 locus?

We provide the figure below for consideration by the reviewer, but we do not see a great fit for this figure in the manuscript.

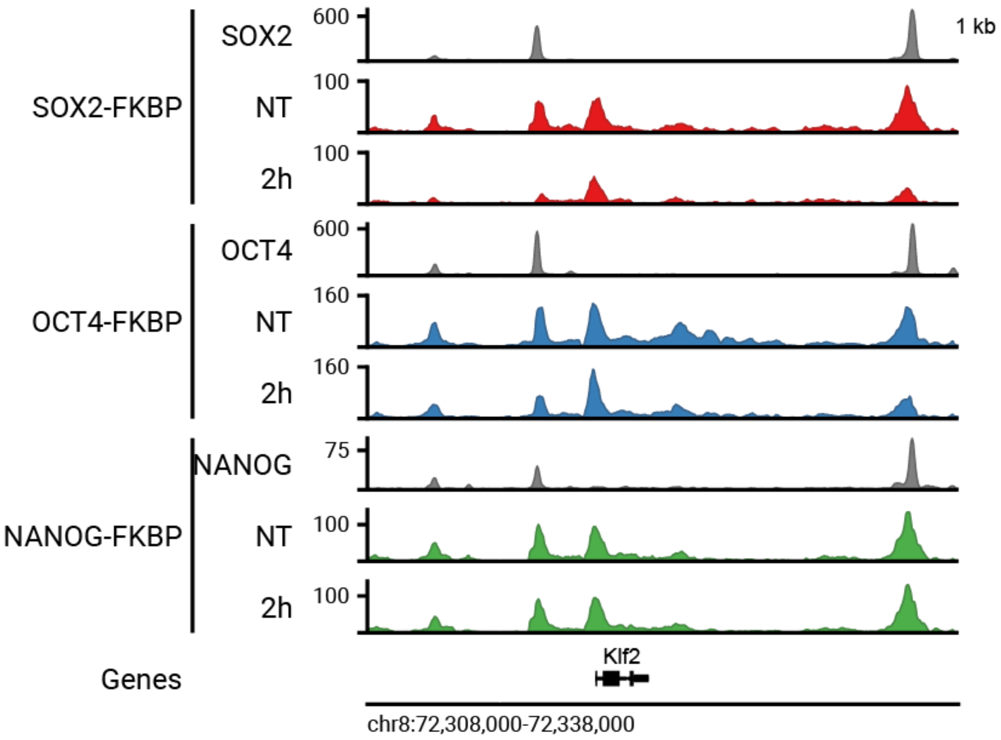

Reviewer figure 11: Example region for the Klf2 accessibility changes as measured by ATACseq after SOX2, OCT4 and NANOG depletion after 2h of dTAG-13 treatment compared to NT. In grey, ChIPseq tracks for SOX2, OCT4 and NANOG. Y-axes represent Reads per genomic content RPGC.

27. In the methods, was there any media change after the addition of dTAG-13 before collection? Please clarify. The sentence of "DMSO was added at the same time as the latest dTAG-13 treatment" is confusing.

We have now added more details in the manuscript about the treatment conditions.

28. ChIP-seq methods needs some textual revisions. Also indicate the sonication time used and what type of beads used should be described.

We have now expanded the ChIPseq protocol adding relevant information. Note that we have now generated and included throughout the manuscript the ChIPseq data obtained using double cross-linked material and have optimized for the background of the antibody.

29. Oligonucleotide table has empty rows, please revise.
We have now updated the oligonucleotide table and have included it in the manuscript as supplementary material.

30. List public datasets used in this study in a supplemental table.
This has been added.

1st Revision - Editorial Decision 8th Jul 2023

Dear Elzo,

Congratulations on a great revision! Overall, the referees have been positive. However there remain some editorial items to attend to in a newly revised version, please update and add this to your point-by-point response:

1. Please including funding information for the following on eJP online: Dutch Cancer Society and of the Dutch Ministry of Health, Welfare and Sport

2. Please include up to five keywords, which may or may not appear in the title, should be given in alphabetical order, below the abstract, each separated by a slash (/).

3. Please rename the data availability section to: Data Availability

4. Please review our new policy on conflict of interests on the EMBO author guide website and update the title of this section to: Disclosure and competing interests statement

5. Please remove the author contribution section from the main manuscript

6. All figures should be referred to in the main text and in chronological order. Please correct the following: Fig. EV2E should be after EV2D; correct this callout Fig EV3 4B to Fig EV3 B

7. Please update the dataset EV legends so they are included in the Excel file as a separate sheet

8. Appendix figure 1 should be included in Appendix PDF file, with the ToC with page numbers. The nomenclature should be Appendix Figure S1 with the corresponding callout

9. We require the publication of source data, particularly for electrophoretic gels and blots and graphs, with the aim of making primary data more accessible and transparent to the reader. It would be great if you could provide me with a PDF file per figure that contains the original, uncropped and unprocessed scans of all or key gels used in the figure or for graphs, an Excel spreadsheet with the original data used to generate the graphs. The PDF files should be labeled with the appropriate figure/panel number, and should have molecular weight marker; further annotation could be useful but is not essential. The PDF files will be published online with the article as supplementary "Source Data" files.

10. We include a synopsis of the paper (see http://emboj.embopress.org/). Please provide me with a general summary statement and 3-5 bullet points that capture the key findings of the paper.

11. We also need a summary figure for the synopsis. The size should be 550 wide by 200-440 high (pixels). You can also use something from the figures if that is easier

12. Attached you will find a word document containing the pre-publication checks performed by our publisher. Please take a look at the file and the comments regarding the figure legends and respond to the issues.

13. For data access, please ensure the data is not private and tokens are provided.

14. Please review our policy online and ensure that the structured methods are formatted correctly

15. We notice a "Reagent Table" has been uploaded in the system separately but it is not a Reagent and tools table as it shows only primer and oligo sequences.

16. Please review our online author guide and correct the order of sections.

Thank you for the opportunity to consider your work for publication. I look forward to your revision.

Kind regards,
Kelly

Kelly M Anderson, PhD
Editor
The EMBO Journal
k.anderson@embojournal.org

Further information is available in our Guide For Authors: https://www.embopress.org/page/journal/14602075/authorguide

----

Referee #2:

The authors have thoroughly revised the manuscript and responded well to all my comments. I have no further concerns and think that the paper could now be a valuable contribution to The EMBO journal.

Referee #3:

The revised manuscript is much improved. The authors have also largely addressed my concerns. I think the manuscript can be accepted for publication at EMBO now.

Editorial items:

1. Please including funding information for the following on eJP online: Dutch Cancer Society and of the Dutch Ministry of Health, Welfare and Sport

Done

2. Please include up to five keywords, which may or may not appear in the title, should be given in alphabetical order, below the abstract, each separated by a slash (/).

Acute protein depletion / Chromatin accessibility / Gene regulation / Transcription factors / Pioneer activity.

3. Please rename the data availability section to: Data Availability

Done

4. Please review our new policy on conflict of interests on the EMBO author guide website and update the title of this section to: Disclosure and competing interests statement

Done

5. Please remove the author contribution section from the main manuscript

Done

6. All figures should be referred to in the main text and in chronological order. Please correct the following: Fig. EV2E should be after EV2D; correct this callout Fig EV3 4B to Fig EV3 B

Done

7. Please update the dataset EV legends so they are included in the Excel file as a separate sheet

We have uploaded the excels sheet of the public datasets used for EV figures and analysis as: public_datasets_ev_used.xlsx

8. Appendix figure 1 should be included in Appendix PDF file, with the ToC with page numbers. The nomenclature should be Appendix Figure S1 with the corresponding callout

Done

9. We require the publication of source data, particularly for electrophoretic gels and blots and graphs, with the aim of making primary data more accessible and transparent to the reader. It would be great if you could provide me with a PDF file per figure that contains the original, uncropped and unprocessed scans of all or key gels used in the figure or for graphs, an Excel spreadsheet with the original data used to generate the graphs. The PDF files should be labeled with the appropriate figure/panel number, and

should have molecular weight marker; further annotation could be useful but is not essential. The PDF files will be published online with the article as supplementary "Source Data" files.

The source data has been uploaded.

10. We include a synopsis of the paper (see http://emboj.embopress.org/). Please provide me with a general summary statement and 3-5 bullet points that capture the key findings of the paper.

General summary: Acute depletion of SOX2 shows that maintaining chromatin accessibility, rather than DNA binding alone, is important for gene regulation in mouse embryonic stem cells.

Bullet points:

- Acute depletion of SOX2 leads to loss of accessibility with a sub-hour time resolution
- Pioneer activity of SOX2 is constantly required to maintain chromatin accessibility
- SOX2 maintains accessible chromatin at a subset of SOX2 binding sites
- SOX2 pioneer sites are a better predictor for transcription changes than SOX2 binding sites

11. We also need a summary figure for the synopsis. The size should be 550 wide by 200-440 high (pixels). You can also use something from the figures if that is easier
This has been uploaded in the manuscript submission system.

12. Attached you will find a word document containing the pre-publication checks performed by our publisher. Please take a look at the file and the comments regarding the figure legends and respond to the issues.
We have looked at the comments and responded to them. Please note that for the replicates regarding the figure EV2, we use public data and for each replicate deposited we run the sampling 100 times.

13. For data access, please ensure the data is not private and tokens are provided.
Data repositories accession numbers have been included.

14. Please review our policy online and ensure that the structured methods are formatted correctly

Done

15. We notice a "Reagent Table" has been uploaded in the system separately but it is not a Reagent and tools table as it shows only primer and oligo sequences.

The Reagent Table has been removed.

16. Please review our online author guide and correct the order of sections.
Done

2nd Revision - Editorial Decision 22nd Jul 2023

Dear Elzo,

Congratulations on an excellent manuscript, I'm pleased to inform you that your manuscript has been accepted for publication in the EMBO Journal. Thank you for your comprehensive response to the referee concerns and for providing source data. It has been a pleasure to work with you to get this to the acceptance stage.

I will begin the final checks on your manuscript before submitting to the publisher next week. Once at the publisher, it will take about three weeks for your manuscript to be published online. As a reminder, the entire review process, including referee concerns and your point-by-point response, will be available to readers.

I will be in touch throughout the final editorial process until publication. In the meantime, I hope you find time to celebrate!

Kind regards,
Kelly

Kelly M Anderson, PhD
Editor, The EMBO Journal
k.anderson@embojournal.org

Please note that it is EMBO Journal policy for the transcript of the editorial process (containing referee reports and your response letter) to be published as an online supplement to each paper. If you do NOT want this, you will need to inform the Editorial Office via email immediately. More information is available here:
https://www.embopress.org/page/journal/14602075/authorguide#transparentprocess

Your manuscript will be processed for publication in the journal by EMBO Press. Manuscripts in the PDF and electronic editions of The EMBO Journal will be copy edited, and you will be provided with page proofs prior to publication. Please note that supplementary information is not included in the proofs.

You will be contacted by Wiley Author Services to complete licensing and payment information. The required 'Page Charges Authorization Form' is available here: https://www.embopress.org/pb-assets/embo-site/tej_apc.pdf - please download and complete the form and return to embopressproduction@wiley.com

EMBO Press participates in many Publish and Read agreements that allow authors to publish Open Access with reduced/no publication charges. Check your eligibility: https://authorservices.wiley.com/author-resources/Journal-Authors/open-access/affiliation-policies-payments/index.html

Should you be planning a Press Release on your article, please get in contact with embojournal@wiley.com as early as possible, in order to coordinate publication and release dates.

EMBO Press Author Checklist

Corresponding Author Name: Elzo de Wit
Journal Submitted to: EMBO Journal
Manuscript Number: #EMBOJ-2022-113150

USEFUL LINKS FOR COMPLETING THIS FORM
The EMBO Journal - Author Guidelines
EMBO Reports - Author Guidelines
Molecular Systems Biology - Author Guidelines
EMBO Molecular Medicine - Author Guidelines

Reporting Checklist for Life Science Articles (updated January

This checklist is adapted from Materials Design Analysis Reporting (MDAR) Checklist for Authors. MDAR establishes a minimum set of requirements in transparent reporting in the life sciences (see Statement of Task: 10.31222/osf.io/9sm4x). Please follow the journal's guidelines in preparing your manuscript.

Please note that a copy of this checklist will be published alongside your article.

Abridged guidelines for figures

1. Data

The data shown in figures should satisfy the following conditions:
- the data were obtained and processed according to the field's best practice and are presented to reflect the results of the experiments in an accurate and unbiased manner.
- ideally, figure panels should include only measurements that are directly comparable to each other and obtained with the same assay.
- plots include clearly labeled error bars for independent experiments and sample sizes. Unless justified, error bars should not be shown for technical
- if n<5, the individual data points from each experiment should be plotted. Any statistical test employed should be justified.
- Source Data should be included to report the data underlying figures according to the guidelines set out in the authorship guidelines on Data

2. Captions

Each figure caption should contain the following information, for each panel where they are relevant:
- a specification of the experimental system investigated (eg cell line, species name).
- the assay(s) and method(s) used to carry out the reported observations and measurements.
- an explicit mention of the biological and chemical entity(ies) that are being measured.
- an explicit mention of the biological and chemical entity(ies) that are altered/varied/perturbed in a controlled manner.
- the exact sample size (n) for each experimental group/condition, given as a number, not a range;
- a description of the sample collection allowing the reader to understand whether the samples represent technical or biological replicates (including how many animals, litters, cultures, etc.).
- a statement of how many times the experiment shown was independently replicated in the laboratory.
- definitions of statistical methods and measures:
  - common tests, such as t-test (please specify whether paired vs. unpaired), simple χ2 tests, Wilcoxon and Mann-Whitney tests, can be unambiguously identified by name only, but more complex techniques should be described in the methods section;
  - are tests one-sided or two-sided?
  - are there adjustments for multiple comparisons?
  - exact statistical test results, e.g., P values = x but not P values < x;
  - definition of 'center values' as median or average;
  - definition of error bars as s.d. or s.e.m.

Please complete ALL of the questions below.
Select "Not Applicable" only when the requested information is not relevant for your study.

Materials

Newly Created Materials	Information included in the manuscript?	In which section is the information available? (Reagents and Tools Table, Materials and Methods, Figures, Data Availability Section)
New materials and reagents need to be available; do any restrictions apply?	Not Applicable	

Antibodies	Information included in the manuscript?	In which section is the information available? (Reagents and Tools Table, Materials and Methods, Figures, Data Availability Section)
For **antibodies** provide the following information:<br>- Commercial antibodies: RRID (if possible) or supplier name, catalogue number and or/clone number<br>- Non-commercial: RRID or citation	Yes	Materials and Methods

DNA and RNA sequences	Information included in the manuscript?	In which section is the information available? (Reagents and Tools Table, Materials and Methods, Figures, Data Availability Section)
**Short novel DNA or RNA including primers, probes**: provide the sequences.	Yes	Reagents and Tool Table

Cell materials	Information included in the manuscript?	In which section is the information available? (Reagents and Tools Table, Materials and Methods, Figures, Data Availability Section)
**Cell lines:** Provide species information, strain. Provide accession number in repository **OR** supplier name, catalog number, clone number, and/**OR** RRID.	Yes	Materials and Methods
**Primary cultures:** Provide species, strain, sex of origin, genetic modification status.	Not Applicable	
Report if the cell lines were recently **authenticated** (e.g., by STR profiling) and tested for mycoplasma contamination.	Not Applicable	

Experimental animals	Information included in the manuscript?	In which section is the information available? (Reagents and Tools Table, Materials and Methods, Figures, Data Availability Section)
**Laboratory animals or Model organisms:** Provide species, strain, sex, age, genetic modification status. Provide accession number in repository **OR** supplier name, catalog number, clone number, **OR** RRID.	Not Applicable	
**Animal observed in or captured from the field:** Provide species, sex, and age where possible.	Not Applicable	
Please detail **housing and husbandry conditions**.	Not Applicable	

Plants and microbes	Information included in the manuscript?	In which section is the information available? (Reagents and Tools Table, Materials and Methods, Figures, Data Availability Section)
**Plants:** provide species and strain, ecotype and cultivar where relevant, unique accession number if available, and source (including location for collected wild specimens).	Not Applicable	
**Microbes:** provide species and strain, unique accession number if available, and source.	Not Applicable	

Human research participants	Information included in the manuscript?	In which section is the information available? (Reagents and Tools Table, Materials and Methods, Figures, Data Availability Section)
If collected and within the bounds of privacy constraints report on age, sex and gender or ethnicity for all study participants.	Not Applicable	

Core facilities	Information included in the manuscript?	In which section is the information available? (Reagents and Tools Table, Materials and Methods, Figures, Data Availability Section)
If your work benefited from core facilities, was their service mentioned in the acknowledgments section?	Yes	Acknowledgment Section

Design

Study protocol	Information included in the manuscript?	In which section is the information available? (Reagents and Tools Table, Materials and Methods, Figures, Data Availability Section)
If study protocol has been **pre-registered, provide DOI in the manuscript**. For clinical trials, provide the trial registration number **OR** cite DOI.	Not Applicable	
Report the **clinical trial registration number** (at ClinicalTrials.gov or equivalent), where applicable.	Not Applicable	

Laboratory protocol	Information included in the manuscript?	In which section is the information available? (Reagents and Tools Table, Materials and Methods, Figures, Data Availability Section)
Provide DOI OR other citation details if **external detailed step-by-step protocols** are available.	Yes	Materials and Methods

Experimental study design and statistics	Information included in the manuscript?	In which section is the information available? (Reagents and Tools Table, Materials and Methods, Figures, Data Availability Section)
Include a statement about **sample size** estimate even if no statistical methods were used.	Not Applicable	
Were any steps taken to minimize the effects of subjective bias when allocating animals/samples to treatment (e.g. **randomization procedure**)? If yes, have they been described?	Not Applicable	
Include a statement about **blinding** even if no blinding was done.	Not Applicable	
Describe **inclusion/exclusion criteria** if samples or animals were excluded from the analysis. Were the criteria pre-established?<br><br>If sample or data points were omitted from analysis, report if this was due to attrition or intentional exclusion and provide justification.	Not Applicable	
For every figure, are **statistical tests** justified as appropriate? Do the data meet the assumptions of the tests (e.g., normal distribution)? Describe any methods used to assess it. Is there an estimate of variation within each group of data? Is the variance similar between the groups that are being statistically compared?	Yes	Materials and Methods

Sample definition and in-laboratory replication	Information included in the manuscript?	In which section is the information available? (Reagents and Tools Table, Materials and Methods, Figures, Data Availability Section)
In the figure legends: state number of times the experiment was **replicated** in laboratory.	Yes	Figures
In the figure legends: define whether data describe **technical or biological replicates**.	Yes	Figures

Ethics

Ethics	Information included in the manuscript?	In which section is the information available? (Reagents and Tools Table, Materials and Methods, Figures, Data Availability Section)
Studies involving **human participants**: State details of **authority granting ethics approval** (IRB or equivalent committee(s), provide reference number for approval.	Not Applicable	
Studies involving **human participants**: Include a statement confirming that **informed consent** was obtained from all subjects and that the experiments conformed to the principles set out in the WMA Declaration of Helsinki and the Department of Health and Human Services Belmont Report.	Not Applicable	
Studies involving **human participants:** For publication of **patient photos**, include a statement confirming that consent to publish was obtained.	Not Applicable	
Studies involving experimental **animals**: State details of **authority granting ethics approval** (IRB or equivalent committee(s), provide reference number for approval. Include a statement of compliance with ethical regulations.	Not Applicable	
Studies involving **specimen and field samples:** State if relevant **permits** obtained, provide details of authority approving study; if none were required, explain why.	Not Applicable	

Dual Use Research of Concern (DURC)	Information included in the manuscript?	In which section is the information available? (Reagents and Tools Table, Materials and Methods, Figures, Data Availability Section)
Could your study fall under dual use research restrictions? Please check biosecurity documents and list of select agents and toxins (CDC): https://www.selectagents.gov/sat/list.htm	Not Applicable	
If you used a select agent, is the security level of the lab appropriate and reported in the manuscript?	Not Applicable	
If a study is subject to dual use research of concern regulations, is the name of the **authority granting approval and reference number** for the regulatory approval provided in the manuscript?	Not Applicable	

Reporting

The MDAR framework recommends adoption of discipline-specific guidelines, established and endorsed through community initiatives. Journals have their own policy about requiring specific guidelines and recommendations to complement MDAR.

Adherence to community standards	Information included in the manuscript?	In which section is the information available? (Reagents and Tools Table, Materials and Methods, Figures, Data Availability Section)
State if relevant guidelines or checklists (e.g., **ICMJE, MIBBI, ARRIVE, PRISMA**) have been followed or provided.	Not Applicable	
For **tumor marker prognostic studies,** we recommend that you follow the **REMARK** reporting guidelines (see link list at top right). See author guidelines, under 'Reporting Guidelines'. Please confirm you have followed these guidelines.	Not Applicable	
For **phase II and III randomized controlled trials**, please refer to the **CONSORT** flow diagram (see link list at top right) and submit the CONSORT checklist (see link list at top right) with your submission. See author guidelines, under 'Reporting Guidelines'. Please confirm you have submitted this list.	Not Applicable	

Data Availability

Data availability	Information included in the manuscript?	In which section is the information available? (Reagents and Tools Table, Materials and Methods, Figures, Data Availability Section)
Have **primary datasets** been deposited according to the journal's guidelines (see 'Data Deposition' section) and the respective accession numbers provided in the Data Availability Section?	Yes	Data Availability Section
Were **human clinical and genomic datasets** deposited in a public access-controlled repository in accordance to ethical obligations to the patients and to the applicable consent agreement?	Not Applicable	
Are **computational models** that are central and integral to a study available without restrictions in a machine-readable form? Were the relevant accession numbers or links provided?	Not Applicable	
If publicly available data were reused, provide the respective **data citations in the reference list**.	Yes	Materials and Methods
